# Systematic verification of bladder cancer-associated tissue protein biomarker candidates in clinical urine specimens

**DOI:** 10.18632/oncotarget.24578

**Published:** 2018-07-20

**Authors:** Cheng-Han Tsai, Yi-Ting Chen, Ying-Hsu Chang, Chuen Hsueh, Chung-Yi Liu, Yu-Sun Chang, Chien-Lun Chen, Jau-Song Yu

**Affiliations:** ^1^ Graduate Institute of Biomedical Sciences, College of Medicine, Chang Gung University, Taoyuan, Taiwan; ^2^ Molecular Medicine Research Center, College of Medicine, Chang Gung University, Taoyuan, Taiwan; ^3^ Department of Biomedical Sciences, College of Medicine, Chang Gung University, Taoyuan, Taiwan; ^4^ Department of Nephrology, Chang Gung Memorial Hospital, Linkou Medical Center, Taoyuan, Taiwan; ^5^ Division of Urology, Department of Surgery, LinKou Chang Gung Memorial Hospital, Taoyuan, Taiwan; ^6^ Graduate Institute of Clinical Medical Sciences, College of Medicine, Chang Gung University, Taoyuan, Taiwan; ^7^ Department of Pathology, Chang Gung Memorial Hospital, Linkou, Chang Gung University, College of Medicine, Taoyuan, Taiwan; ^8^ Department of Otolaryngology - Head & Neck Surgery, Chang Gung Memorial Hospital, Linkou, Taiwan; ^9^ Department of Urology, Chang Gung Memorial Hospital, Taoyuan, Taiwan; ^10^ College of Medicine, Chang Gung University, Taoyuan, Taiwan; ^11^ Liver Research Center, Chang Gung Memorial Hospital, Linkou, Taiwan; ^12^ Research Center for Food and Cosmetic Safety, Research Center for Chinese Herbal Medicine, College of Human Ecology, Chang Gung University of Science and Technology, Taoyuan, Taiwan

**Keywords:** bladder cancer, biomarker verification, MRM, targeted proteomics, protein quantification

## Abstract

Bladder cancer biomarkers currently approved by the Food and Drug Administration are insufficiently reliable for use in non-invasive clinical diagnosis. Verification/validation of numerous biomarker candidates for BC detection is a crucial bottleneck for novel biomarker development. A multiplexed liquid chromatography multiple-reaction-monitoring mass spectrometry assay of 122 proteins, including 118 up-regulated tissue proteins, two known bladder cancer biomarkers and two housekeeping gene products, was successfully established for protein quantification in clinical urine specimens. Quantification of 122 proteins was performed on a large cohort of urine specimens representing a variety of conditions, including 142 hernia, 126 bladder cancer, 67 hematuria, and 59 urinary tract infection samples. ANXA3 (annexin A3) and HSPE1 (heat shock protein family E member 1), which showed the highest detection frequency in bladder cancer samples, were selected for further validation. Western blotting showed that urinary ANXA3 and HSPE1 protein levels were higher in bladder cancer samples than in hernia samples, and enzyme-linked immunosorbent assays confirmed a higher urinary concentration of HSPE1 in bladder cancer than in hernia, hematuria and urinary tract infection. Immunohistochemical analyses showed significantly elevated levels of HSPE1 in tumor cells compared with non-cancerous bladder epithelial cells, suggesting that HSPE1 could be a useful tumor tissue marker for the specific detection of bladder cancer. Collectively, our findings provide valuable information for future validation of potential biomarkers for bladder cancer diagnosis.

## INTRODUCTION

Bladder cancer (BC) is one of the most common urinary tract carcinomas. On the basis of global cancer statistics, it is estimated that BC accounted for 330,400 new cases of cancer and 123,100 cancer-related deaths worldwide during 2012, making it the sixth-most common cancer and the ninth-leading cause of death [1]. The incidence of BC increases with age, peaking between age 50 and 70, and is three times more common in men than women [2]. In general, more than 90% of BC patients are diagnosed with transitional cell carcinoma, 5% with squamous cell carcinoma, and less than 2% with adenocarcinoma [3]. Of diagnosed BC cases, approximately 70%–80% will present with non-muscle–invasive carcinoma, 50%–70% will recur, and 10%–30% will progress to muscle-invasive disease [4]. In most cases, recurrence takes place within 5 years, and patients with higher-grade lesions are at greater risk for tumor progression [5]. Clinical statistical results cited above also highlight the importance of early detection in the management of BC.

The diagnosis of BC mainly relies on urethroscopy and cytology. Cystoscopy detects most papillary and solid lesions, but is invasive [6], whereas urine cytology has reasonable sensitivity and specificity for the detection of high-grade BC, but its sensitivity for detecting low-grade tumors only ranges from 4% to 31% [7]. This limitation of cytology and the invasiveness of urethroscopy for clinical detection have generated interest in the development of novel non-invasive urinary markers for the diagnosis of BC. A biomarker is a molecular correlate that provides an indication of disease progression and prognosis status of a patient. Two urinary protein biomarkers of BC approved by the US Food and Drug Administration (FDA) are commercially available to complement urethroscopy in the diagnosis of BC. One is nuclear matrix protein 22 (NMP 22), a nuclear matrix protein that is an important regulator of mitosis. It has been demonstrated that the urinary concentration of this protein is 5–25-fold higher in BC cell lines than in normal urothelium [8, 9]. Grossman *et al.* compared NMP22 assessment to cytology in the detection of BC in 1331 patients [10], demonstrating that the sensitivity and specificity of NMP22 for BC detection was 55.7% and 85.7%, respectively. By comparison, the sensitivity and specificity of cytology was 15.8% and 99.2%, respectively [10]. However, the concentration of NMP22 may also be elevated in patients with pyuria, urolithiasis, hematuria, or cystitis [11]. A recent analysis of validation results [12] suggests that NMP22 cannot be considered a replacement for urethroscopy in detecting BC. The alternative urinary biomarker, bladder tumor antigen (BTA), is a human complement factor H-related protein that cell culture studies have shown is only expressed in cancer cells [13]. BTA-TRAK assays measure factor H-related protein using a standard, quantitative, enzyme-linked immunosorbent assay (ELISA) [11], and BTA-stat is a rapid, qualitative, immunochromatographic assay that is approved by the FDA for monitoring, but not for diagnosis. The sensitivity and specificity of BTA-stat were reported to be 70% and 75%, respectively, whereas the corresponding values for BTA-TRAK are 66% and 65% [14]. Compared with healthy individual controls, the reported specificity of BTA-stat is 97%, but the specificity of BC diagnosis drops to 46% in comparisons with patients with benign genitourinary conditions [15]. Hematuria and benign genitourinary conditions may lead to false-positive results in both tests [16, 17]. Because of such high false-positive rates, these two protein biomarkers were recommended for use only in combination with urethroscopy for the detection of BC. Thus, there is a need for the development of more reliable bladder tumor markers.

In the last decade, technological advancements in biomarker discovery have resulted in reports of a large number of potential protein biomarkers [18–22]. Although increasing numbers of protein biomarkers have been reported, only a few proteins have been approved by the FDA [23]. The bottleneck for approval is biomarker verification and validation-the actual sensitivity and specificity of biomarker candidates must be validated in a large number of clinical samples before clinical application [24]. The ELISA is one method that is widely used in biomarker validation, but development of individual ELISAs for large numbers of potential biomarkers is very time consuming and expensive for novel proteins [25]. Therefore, the purpose of the initial verification phase of biomarker development is to prioritize better-performing biomarker candidates, which are then passed on to a final validation phase. Mass spectrometry (MS) plays an important role not only in the discovery phase, but also in the methodological verification phase. Targeted MS is becoming widely used in quantitative proteomics approaches for biomarker verification methodologically. In recent years, multiple reaction monitoring (MRM)-MS for quantification of peptides derived from protein candidates has been applied to biomarker verification [26]. MRM-MS can detect multiplex peptides in a single MS run using stable isotope-labeled standards to precisely quantify peptides [27, 28]. Because of this advantage, MRM-MS can perform high-throughput, multiplexed verifications of biomarker candidates in a short period of time.

In our previous work [18], we discovered 130 up-regulated BC tissue proteins using a strategy that combined laser microdissection, isobaric tags for relative and absolute quantitation labeling (iTRAQ), and liquid chromatography-tandem MS (LC-MS/MS) analysis to profile proteomic changes in fresh-frozen bladder tumor specimens. Among these BC biomarker candidates, many have not been verified systematically in additional clinical specimens. In the current study, we extended the methodological verification of these 130 biomarker candidates by establishing a detection workflow using isotopic dimethyl labeling coupled with MRM-MS. The proteins that showed elevated concentrations in BC urine, representing potential non-invasive biomarkers, were further verified in additional urine specimens using ELISAs. Expression of the verified protein biomarker candidates in tissue specimens was also tested by immunohistochemistry (IHC) to confirm their bladder cell origin.

## RESULTS

### Selection of protein biomarker candidates from the BC tissue proteome for MRM-MS assay development

The main function of the bladder is the collection, temporary storage, and expulsion of urine. Bladder tumor frequently occurs in the urothelium layer of urinary bladder. Urine is a proximal fluid for the bladder, thus we expect that the secretory proteins of bladder tumor tissue may secrete or release to urine, resulting in the elevated concentrations in urine. In our previous study of the bladder tissue proteome [18], we isolated tumor tissue and adjacent non-tumor tissue cells from four individual BC patients for comparison by a strategy combining laser capture microdissection (LCM), iTRAQ, and LC-MS/MS. These proteomic analyses [18] revealed that 130 candidates were up-regulated (tumor/non-tumor [T/N] ≥ 1.5) in at least 50% of BC patients. In this study, we planned to perform the methodological verification of these novel tissue biomarkers in a medium-scale of clinical urine and tissue specimens. The verified protein biomarker candidate will be used for large-scale validation work in the future.

These 130 candidates were selected for MRM-MS assay development and methodological verification (Supplementary Table 1). Additionally, two known potential BC markers, NUMA1 (nuclear mitotic apparatus protein 1) and CFHR1 (complement factor H related 1), and two housekeeping genes, ACTB (β-actin) and TUBB (tubulin-β), reported in previous studies [8, 29–32], were also included as controls for evaluation of the MRM-MS assay.

### Establishment of the MRM assay: prescreening dimethyl-labeled signature peptide candidates in BC cell extracts

A total of 130 proteins were selected for development of the MRM-MS method and multiplex quantification in urine for methodological verification of future non-invasive biomarkers. As shown in the strategy depicted in Supplementary Figure 1, protein sequences were *in silico*-digested to generate a dimethylated peptide list using MRMPilot software. This bioinformatics analysis resulted in 4400 Q1/Q3 ion transitions for 1459 peptides corresponding to 130 proteins (Supplementary Figure 1 and Supplementary Table 2). To select the most sensitive peptide sequences for subsequent MRM quantification, we included all the predicted/theoretical qualified peptide sequences (4400 Q1/Q3 ion transitions) for MRM-MS detection in the first stage of method development using BC cell lysates. These 4400 transitions for 1459 signature peptides were divided into 44 MRM-MS methods and analyzed in MRM-initiated detection and sequencing (MIDAS) mode to confirm their detectability in BC cells and urine [33]. BC cell extracts were used because their proteomic composition is more similar to tissue than urine samples, and they are easier to obtain than clinical tissue. The cell line data were then used for selection of top-3 Q1/Q3 transitions with strong intensities and also for evaluation of background interference for each tryptic peptide in MRM-MS spectra. Accordingly, extracts of the BC cell lines, 5637, BFTC 905 and TSGH 8301, were used for evaluating the detectability of *in silico* tryptic-digested peptides. Proteins from the three BC cell lines were digested and mixed in equal amounts. MS/MS spectra, triggered by detectable Q3 signals in the MRM mode, of the dimethyl labeled peptide mixtures were analyzed using a QTRAP 5500 MS in MIDAS mode. Acquired MS/MS spectra of target peptides were identified using a Mascot Daemon database search, which confirmed the correct peptide sequence. Identified signature peptides were further confirmed by elution time, ion score, multiple transitions, and protein assignment in Mascot Daemon results. Co-elution of multiple Q1/Q3 transitions from a single peptide at the same retention time and correctness of MS/MS spectra in MS raw files were also verified using Analyst software (Supplementary Figure 2) and manually. As shown in the example in Supplementary Figure 2A, the ion score, detected product ions, intensity ranking, and co-elution of multiple transitions of ANXA3 peptide (GIGTDEFTLNR) were matched with the protein sequence database using Mascot Daemon and confirmed with Analyst software. A total of 30 candidate proteins (48 signature peptides) without detectable predicted Q1/Q3 transitions in BC cell extracts were quantified using detected peptide sequences identified in the LCM tissue proteome [18] (Supplementary Figure 1). The uniqueness of signature peptides was confirmed using the Basic Local Alignment Search Tool (BLAST) on the UniProt website (http://www.uniprot.org/blast/), eliminating interferences from other proteins with shared sequences. The signature peptides and their top three Q1/Q3 transitions with high intensities were used for subsequent quantification in the final MRM assay (Supplementary Figures 1 and 2B and Supplementary Table 3). Measurement of a protein was based on one tryptic dimethylated peptide. Among the original 130 target proteins, 119 peptides corresponding to 119 proteins were determined successfully. No suitable tryptic peptides were available for the remaining eleven proteins (Supplementary Table 1), which could thus not be quantified using MRM-MS in this study. We also combined two known BC markers (NUMA1, CFHR1) and two housekeeping genes (ACTB, TUBB) that were reported in previous studies [8, 29–32] (Supplementary Figure 1 and Supplementary Table 3). Only one peptide (TPQGPGR, derived from SLC4A2) failed to yield a quantifiable MRM signal because of its high hydrophilicity, which resulted in an unstable MRM signal intensity or poor LC column retainability in samples prepared in the same manner from different individual specimens. In all, 122 peptides, which were subsequently obtained as commercially synthesized peptides, were selected for establishment of a multiplexed MRM MS assay.

### Establishment of the MRM assay: selection of the top three transitions and collision energy (CE) optimization

As described above, we selected a signature peptide for each protein for development of the final MRM-MS assay. MRM hardware parameters were then optimized using 122 dimethylated synthetic peptides as materials. Three detected transitions and the corresponding MRM parameters of each peptide are listed in Supplementary Table 3. Collision energy (CE), the most influential parameter, was optimized using the CE ramping function by infusing dimethylated synthetic peptides into a QTRAP mass spectrometer to generate strong Q3 signals. CE ramping experiments tested five levels with CE value differences of 3 V, using the default CE value predicted by MRMPilot software as the middle value. Two types of CE optimization results were obtained. In the first, the default CE predicted by MRMPilot is the best value resulting from CE ramping (Supplementary Figure 3A). For example, the best CE for the IGFBP7 (insulin like growth factor binding protein 7) peptide, DNLAIQTR (Q1/Q3:482.8/517.3), was 26 V, which is the default setting of MRMPilot software; this was then used to generate the highest Q3 ion intensity (517.3 m/z). Alternatively, the best CE could be higher or lower than the default setting (Supplementary Figure 3B, C and D). These transitions were then further evaluated using an extensive range of CE ramping. As another example, the better signal for TSN protein (Q1/Q3 as582.8/475.3) was 37 V; this value is higher than predicted default value of 31 V and was detected at a higher CE after CE re-ramping (Supplementary Figure 3E). After a second round of CE ramping, the optimized CE was determined to be 40 V, resulting in a 31.4% higher MRM peak intensity compared with the default CE value. The ΔCE values (optimized CE – default CE) for all transitions are summarized in Supplementary Figure 4. These data indicate the importance of CE optimization: although, in theory, the software calculates the default CE, the best CE may require adjustment for different types of instruments and peptide sequences. The CE-optimized multiplexed MRM-MS assay was established for quantification of 122 peptides corresponding to the 122 protein biomarker candidates for BC using two MS methods (Supplementary Table 3).

### Methodological verification of 122 protein candidates in clinical urine samples by MRM-MS

**Figure 1:**
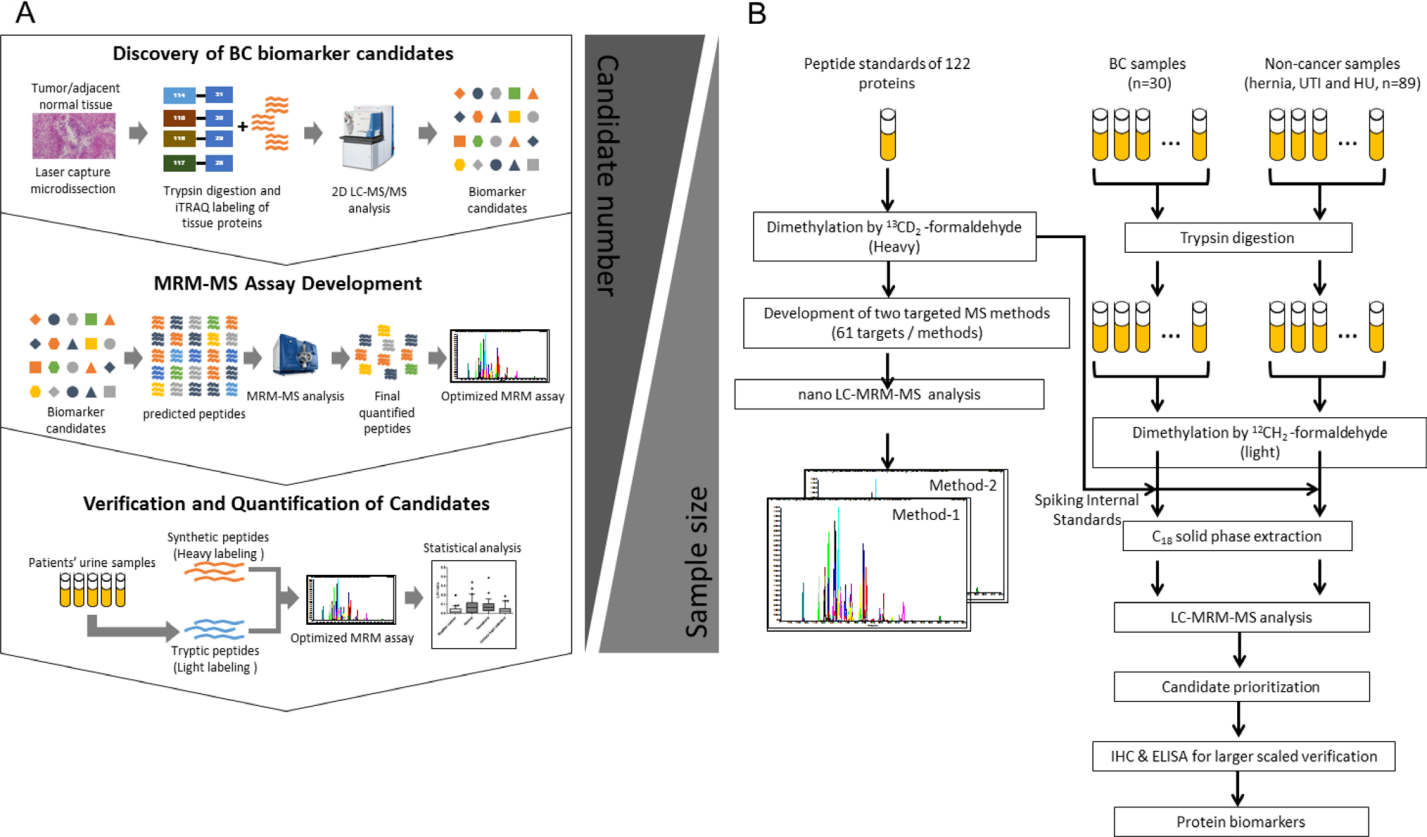
Workflow for the methodological verification of protein biomarker candidates in clinical urine samples (**A**) Strategy for biomarker discovery and methodological verification. I. Discovery phase: Up-regulated candidates for MRM-MS assay development were identified by an analysis of the BC tissue proteome. II. MRM-MS assay development: 130 up-regulated candidates (T/N ≥ 1.5) were selected for assay development. Dimethylated peptides and transitions of each protein candidate were predicted by Skyline software and used for the development of the MRM-MS method. III. Candidate verification methodologically: Protein targets in clinical urine samples were quantified using the MRM-MS assay. A total of 119 urine samples from BC, hernia, hematuria, and UTI patients were tested. All candidates were quantified and verified using MRM-MS based on the fixed amount of heavy-labeled dimethylated synthetic peptides. The diagnostic power of potential biomarkers was estimated by statistical analyses of multiplexed MRM-MS data. (**B**) Proteins in urine samples from individual BC patients (*n* = 30) and non-cancer patients (*n* = 89) were extracted for tryptic digestion and light isotopic dimethylation. Each individual sample was spiked with 122 heavy dimethyl-labeled peptide standards, which were subsequently detected by LC-MRM-MS.

Clinical urine samples from individual patients in different clinical groups, including BC, hernia, hematuria and UTI, were collected for methodological verification. The sample size and patient age and sex for four clinical groups are shown in Table 1. The strategy for methodological verification of BC-associated up-regulated tissue proteins in urine and the workflow scheme are shown in Figure 1A and 1B. Triplicate independent tryptic digestion experiments for all individual samples were processed randomly. MRM-MS results (Table 2) showed that 29 proteins targets could be detected in at least one clinical urine samples. Among these 29 proteins, eight were detected in more than 50% of all samples. The mean, median and standard deviation of the coefficient of variation (CV) obtained from quantitative analyses of the 29 peptides are shown in Supplementary Table 4. The CV values of quantified results for 18 proteins were less than 30% in all samples. Concentration differences in the 12 proteins that were detectable in more than 15% of all individual urine samples were further evaluated according to *p*-value and fold changes (Supplementary Table 5). However, preliminary tests of clinical samples revealed no promising proteins worthy of further verification, probably owing to the limited MRM-MS sample size (data not shown). To select proteins with better specificity in BC, we then selected the proteins that were only detectable in BC for further verification by antibody-based methods. Twelve urinary proteins-ANXA3 (annexin a3), CA2 (carbonic anhydrase 2), CLTA (clathrin light chain A), GLO1 (glyoxalase I), HSPE1, SERPINB5 (serpin family B member 5), SFN (stratifin), SLC3A2 (solute carrier family 3 member 2), TAGLN2 (transgelin 2), TIMM13 (translocase of inner mitochondrial membrane 13), VPS29 (Isoform 1 of Vacuolar protein sorting-associated protein 29) and YWHAQ (tyrosine 3-monooxygenase/tryptophan 5-monooxygenase activation protein theta)-were only detected in BC urine specimens, and not in samples from hernia patients (Table 2).

**Table 1:** Clinical information of the clinical specimens used for MRM-MS

	Classification	Diagnosis status	Sex	Number	Age
Non-tumor control	Hernia	Hernia	male	30	63.4 ± 10.2
			female	1	67.0
	Hematuria (HU)	Hematuria (stone), RBC>20(HU)	male	27	57.0 ± 12.9
			female	3	69.3 ± 7.4
	Urinary tract infection (UTI)	Urinary tract infection, WBC>30(UTI)	male	16	58.1 ± 8.5
			female	12	59.7 ± 9.6
Bladder carcinoma	Low grade/Early stage (LgEs)	Low grade, pTa, pT1	male	9	62.0 ± 8.5
			female	1	72.0
	High grade/Early stage (HgEs)	High grade, pTa, pT1	male	7	62.1 ± 11.3
			female	3	73.7 ± 16.6
	High grade/Advanced stage (HgAs)	High grade, pT2, pT3, pT3b, pT3bN1	male	8	72.4 ± 15.4
			female	2	62.0 ± 7.1

**Table 2:** 29 detectable protein targets in cancer and non-cancer urine samples and their possible secretory pathways

Genes	Hernia (*n* = 31)	Bladder cancer (*n* = 30)	Bladder cancer (*n* = 30)			Hematuria (*n* = 30)	UTI (*n* = 28)	All samples (*n* = 119)		Classically secreted proteins by SignalP	Non-classically secreted proteins by SecretomeP	Bladder cancer urinary microparticle proteome [39]	Secretome of BC cell lines [35]
			LgEs	HgEs	HgAs			Number	%				
SERPINA3	31	28	9	9	10	30	25	114	95.80%	●	●	●	
ACTB	24	27	10	7	10	24	24	99	83.19%				
LAMP2	24	23	9	4	10	24	21	92	77.31%		●	●	
TPI1	24	27	10	7	10	20	18	89	74.79%			●	●
VTN	22	23	10	3	10	20	23	88	73.95%	●	●	●	●
RNASET2	25	15	2	7	6	28	28	96	80.67%	●	●	●	●
DPP7	27	19	6	6	7	17	24	87	73.11%	●	●	●	●
GAA	21	25	10	8	7	18	13	77	64.71%	●	●	●	
HSP90AB1	10	16	4	3	9	13	17	56	47.06%			●	
IGFBP7	16	13	4	2	7	11	6	46	38.66%	●		●	●
ENO1	0	4	1	0	3	7	9	20	16.81%			●	●
RAB11B	6	7	4	2	1	4	4	21	17.65%			●	●
PRPF3	1	4	1	1	2	4	3	12	10.08%		●		
PSPH	5	8	3	5	0	0	1	14	11.76%			●	●
ANXA3	0	3	0	0	3	0	2	5	4.20%			●	●
TAGLN2	0	4	0	1	3	0	0	4	3.36%		●	●	●
HSPE1	0	3	0	0	3	0	1	4	3.36%				●
TIMM13	0	2	0	0	2	1	1	4	3.36%		●		●
SLC3A2	0	2	0	2	0	0	0	2	1.68%		●	●	●
YWHAQ	0	3	1	0	2	0	0	3	2.52%			●	●
CLTA	0	2	2	0	0	0	0	2	1.68%		●		●
BAIAP2	1	1	1	0	0	0	0	2	1.68%			●	●
GLO1	0	2	2	0	0	0	0	2	1.68%			●	●
CA2	0	2	0	0	2	0	0	2	1.68%			●	●
SERPINB5_maspin	0	1	0	0	1	0	0	1	0.84%			●	●
SFN	0	1	0	0	1	0	0	1	0.84%			●	●
LOC643576	0	0	0	0	0	1	0	1	0.84%				●
VPS29	0	1	0	1	0	0	0	1	0.84%			●	●
WASF2	0	0	0	0	0	0	1	1	0.84%			●	●

### Validation of prioritized proteins in clinical urine and tissue samples by Western blotting

**Figure 2:**
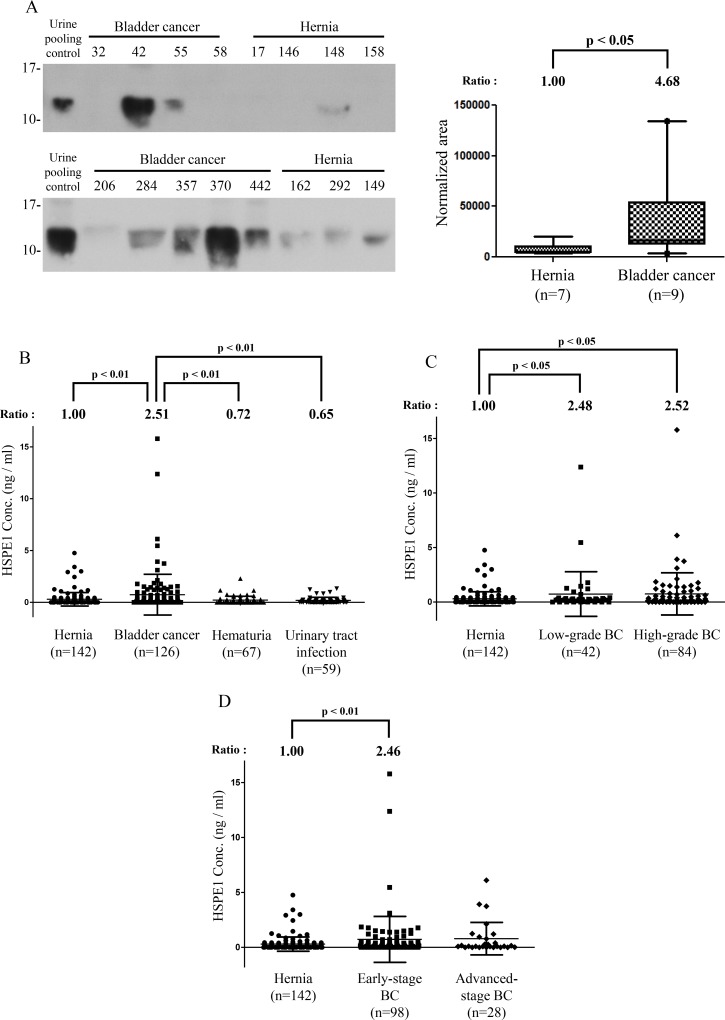
Methodological verification of HSPE1 in individual urine specimens from BC and hernia patients by antibody-based analysis (**A**) Expression of HSPE1 in individual clinical urine specimens from BC and hernia patients, detected by Western blot analysis using an anti-HSPE1 antibody. A pooled urine sample prepared from BC and hernia patients was used as control for comparison of multiple gels. Data were quantified using Image J software. (**B**–**D**) Results of statistical analyses of HSPE1 expression in urine from hernia and BC patients by ELISA. (B) Differences in HSPE1 concentrations in urine specimens from hernia (*n* = 142), BC (*n* = 59), hematuria (*n* = 67) and UTI (*n* = 59) patients. (C) Difference in HSPE1 concentration in urine specimens from hernia (*n* = 142), low-grade BC (*n* = 42) and high-grade BC (*n* = 84) patients. (D) Difference in HSPE1 concentration in urine specimens from hernia (*n* = 142), early-stage BC (*n* = 98), and advanced-stage BC (*n* = 28) patients. Differences between groups were determined using the Mann-Whitney test.

The availability of commercial antibodies for Western blot detection in BC cell lines or urine proteins of individual clinical specimens limited our analysis of the twelve proteins to ANXA3, VPS29, YWHAQ, and HSPE1 (data not show). In initial tests, only ANXA3 and HSPE1 showed an increase in concentration in the urine of BC patients. Because ANXA3 has been reported to be a biomarker for other urothelial carcinomas [34], we focused on HSPE1 expression in clinical samples, validating its concentration level in individual urine samples. Western blotting indicated that the urinary concentration of HSPE1 was significantly higher (~4.7-fold) in individual BC patients than in hernia patients (*P* < 0.05, *n* = 16; Figure 2A). The alternative immune-based method again confirmed MRM-MS identification of urinary HSPE1 as a potential BC biomarker for BC diagnosis.

### Validation of HSPE1 in clinical urine samples by sandwich ELISA

To further confirm the utility of HSPE1 as a non-invasive biomarker for BC diagnosis, we measured the concentration of HSPE1 in a large number of urine specimens from 142 hernia, 126 bladder cancer, 67 hematuria, and 59 urinary tract infection patients by sandwich ELISA. As shown in Figure 2B, the concentration of HSPE1 in urine from BC patients was 2.5-fold higher than that in urine from hernia patients (*P* < 0.01, total *n* = 268). Differences between BC and non-cancerous groups are summarized in Table 3. As shown in Figure 2C and 2D, the urinary concentration of HSPE1 in early-stage and low-grade BC were both significantly higher (at least 2.46-fold) than that in hernia. As shown in Table 3, the area-under-the-curve (AUC) value was 0.60 in low-grade BC (*P* < 0.05; H, *n* = 142; low-grade BC, *n* = 42), and 0.61 in early-stage BC (*P* < 0.01, H, *n* = 142, early-stage BC, *n* = 98) using hernia urine specimens as control samples. These results indicate that urinary HSPE1 is a potential candidate for the non-invasive early detection of BC. Additionally, the concentration of HSPE1 in urine from BC patients was 3.49- and 3.86-fold higher than that in urine from hematuria and UTI patients, respectively (Figure 2B). The AUC value for BC differentiation was 0.63 when comparing with hematuria (*P* < 0.05; HU, *n* = 67; BC, *n* = 126), or UTI patients (*P* < 0.01, UTI, *n* = 59, BC, *n* = 126) as control samples (Table 3). We further evaluated the effect of blood contamination and urinary tract infection in urinary HSPE1 concentration of BC patients. The urine red blood cells (RBC) and white blood cells (WBC) tests in urine are the most common way to measure the presence of hematuria, and UTI/inflammation. We have examined the correlations between RBC and the BC urinary levels of HSPE1 as Supplementary Figure 5, which showed a poor correlation (Spearman *r* = 0.2541, *n* = 119). The result of WBC test also shown the poor correlation with BC urinary levels of HSPE1 (Spearman *r* = 0.1964, *n* = 119). Additionally, correlation analyses between urinary HSPE1 expression and clinicopathological characteristics of urine specimens from bladder cancer patients were assessed using a Chi-squared test. Supplementary Table 6 confirmed that the expression of urinary HSPE1 was not significantly correlated with the characteristics of age, sex, histologic grade, TNM stage, hematuria and urinary tract infection. These results indicate that urinary HSPE1 is a potential candidate for the non-invasive early detection of BC and can be used to discriminate BC from hernia, hematuria, and UTI.

### Immunohistochemical detection of HSPE1 in clinical tissue samples

**Figure 3:**
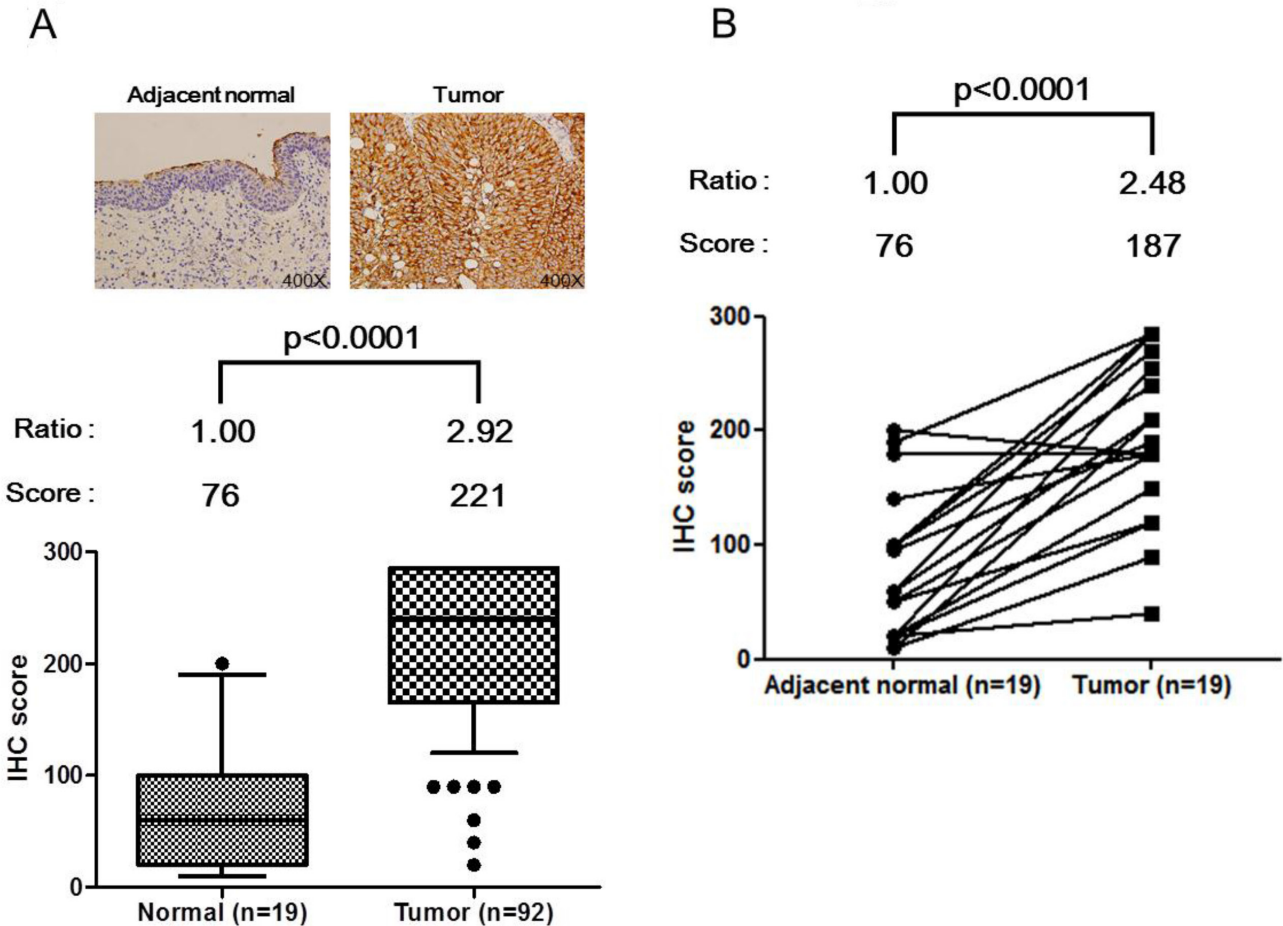
Up-regulation of HSPE1 in individual bladder tumor tissue specimens, determined by IHC (**A**) IHC scores for normal tissue (*n* = 19) and tumor tissue (*n* = 92) in bladder tumor slides. (**B**) IHC score for adjacent normal tissue (*n* = 19) and paired tumor tissue (*n* = 19) in paired bladder tumor slides. HSPE1 expression data for normal and tumor tissue cells from a given individual are linked with dashed lines. Differences between groups were determined using the Mann-Whitney test for (A) and paired Student's *t*-test for (B).

The above data demonstrate an increased concentration of HSPE1 in BC urine. To verify the expression of HSPE1 in cells in BC tissue-a possible source of urinary HSPE1-we assessed HSPE1 in formalin-fixed paraffin-embedded (FFPE) clinical tissue specimens by immunohistochemistry (IHC). As shown in Figure 3A and Supplementary Table 7, IHC scoring results indicate that HSPE1 expression levels in bladder tumor tissues were ~2.9-fold higher than those in normal tissues (*P* < 0.0001). We also confirmed that HSPE1 expression in paired tissue from the 19 individuals was significantly higher in tumor tissue (average, ~2.5 fold-change) than in adjacent normal tissues (*p* < 0.0001), noted by dashed lines in Figure 3B. The ability of HSPE1 to differentiate BC was accessed by a receiver operator curve (ROC) analysis of clinical tumor tissue. As shown in Table 3, the AUC of the ROC was 0.92 (*p* < 0.0001, *n* = 92 vs. 19). Correlations between HSPE1 expression and clinicopathological characteristics of tissue specimens were assessed using a Chi-squared test. As shown in Table 4, the IHC score for HSPE1 was significantly correlated with tumor size (*p* = 0.021), with 86.8% of slices from larger tumors (>2 cm) having a high IHC score (151–300) compared with only 66.7% of slices with small tumors (≤2 cm). IHC scores for HSPE1 were also significantly correlated with TNM stage (*p* = 0.028), with tissue specimens from BC patients with late-stage tumors (III + IV) showing higher IHC scores than patients with early-stage tumors (I + II). These results indicate that HSPE1 might be a potential molecular marker for evaluating tumor progression. Taken together, the above results suggest that high expression of HSPE1 in BC tissue represents a TNM stage-dependent marker that correlates with clinicopathological characteristics. HSPE1 overexpressed in BC tissue cells may be secreted into urine, resulting in higher concentrations in BC urine specimens than control samples.

**Table 3:** The *p*-values, fold changes, sensitivity, specificity, and AUC values of HSPE1 ROC curves

Expression level of HSPE1	Different comparison	Fold change (BC/control)	*p*-value for diagnosis	AUC (sensitivity/specificity)	Optimal cutoff point (score for tissue; ng/ml for urine)
Tissue	Bladder cancer (*n* = 92) vs Adjacent normal (*n* = 19)	4.68	<0.0001	0.92 (92.39%/78.95%)	110
Urine	Bladder cancer (*n* = 126) vs Hernia (*n* = 142)	2.51	<0.01	0.60 (75.40%/42.25%)	0.074
	Low-grade BC (*n* = 42) vs Hernia (*n* = 142)	2.48	<0.05	0.60 (59.52%/68.31%)	0.204
	Early-stage BC (*n* = 98) vs Hernia (*n* = 142)	2.46	<0.01	0.61 (52.04%/68.31%)	0.204
	Bladder cancer (*n* = 126) vs Hematuria (*n* = 67)	3.48	<0.01	0.63 (57.94%/65.67%)	0.155
	Bladder cancer (*n* = 126) vs Urinary tract infection (*n* = 59)	3.87	<0.01	0.63 (38.10%/84.75%)	0.261

**Table 4:** Correlations of HSPE1 protein expression (IHC scores) in tissue specimens w ith clinicopathological characteristics

Characteristics	*n*	HSPE1 expression (score)		*p* values
		low	high	
		(0–150)	(151–300)	
Age				
≤65 years	32	6	26	0.612
>65 years	60	14	46	
Sex				
Male	48	10	38	0.826
Female	44	10	34	
Size of tumor				
≤2cm	39	13	26	0.021
>2 cm	53	7	46	
Histologic grade				
Low grade	10	4	6	0.115
High grade	81	15	66	
Tumor status				
Ta + T1	43	13	30	0.064
≥T2	49	7	42	
Lymph node metastasis				
N0	76	18	58	0.922
N1 + N2	9	2	7	
Distant metastasis				
M0	49	16	33	NA
M1	0	0	0	
TNM stage				
I + II	59	17	42	0.028
III + IV	33	3	30	

## DISCUSSION

In this study, we sought to verify hundreds of novel BC-associated tissue protein biomarker candidates discovered in an analysis of the BC tissue proteome [18] by developing an MRM-MS assay capable of detecting and quantifying BC-derived proteins in urine, a proximal fluid for the bladder, for use as a non-invasive BC diagnostic biomarker. These 130 tissue proteins were further analyzed by SignalP and SecretomeP programs designed to predict the classical and non-classical protein secretion pathways, respectively. Among the 130 tissue proteins up-expressed in bladder cancer tissue, 27 proteins were predicted as normally secreted proteins by SignalP analysis (> default D-cutoff value) based on the presence of a signal peptide. The SecretomeP program predicted that 33 proteins were released via the nonclassical secretory pathway (SecretomeP score ≥ 0.6 and without a signal peptide). A total of 73 proteins were also identified in the urine specimens of microparticle/exosome proteome from bladder cancer patients. 82 proteins were detectable in the secretome datasets [35] of two bladder cancer cell lines, U1 and U4. After combination of these analyses, a total of 115 bladder cancer-associated proteins were predicted as proteins with secretion properties (Table 2 and Supplementary Table 1) and are potentially to be secreted into urine. However, these bladder tissue-derived proteins become highly diluted in urine, yielding concentrations in the nanogram-per-milliliter range and below [18, 36], and are likely interfered with by other more abundant urinary proteins during multiplexed quantification. To efficiently identify these novel and highly diluted BC-specific tissue proteins in urine across a large number of samples, we selected MRM-based technology, with its precise and reproducible quantification, as a platform for multiplexed quantification. Software was used to predict suitable Q1 and Q3 pairs for MRM-MS assays. Because of the rareness of clinical BC tissue specimens and the highly dynamic range of protein concentrations in clinical urine specimens, we used BC cell line extracts as the material for MRM-MS method optimization. This material is also preferable for this application because proteomic profiles of tissue and cell extracts are more similar than those of tissue and urine. Of the 130 BC-associated tissue proteins, 89 (68.5%) were detectable in BC cell line extracts (Supplementary Figure 1). These results indicate that cell extracts are suitable alternative samples for prescreening of BC-associated tissue protein biomarker candidates. After prescreening 1459 signature peptides for these 130 candidates in BC cell extracts, we selected one signature peptide for each protein for quantification in a larger number of individual clinical urine specimens. In the case of 12 candidates, no suitable signature peptides could be entered into the MRM-MS assay development pipeline owing to the lack of qualified peptide sequences or MRM signal stability. Thus, the MRM-MS assay was ultimately successfully established based on 118 proteins for subsequent quantification in urine samples. Stable isotopic dimethyl labeling, which dramatically reduces the cost of synthetic peptides, was used to select signature peptides during early-stage development. Stable isotope dimethyl labeling is an easy-to-manage and cost-effective method that can be applied to any type of sample, including tissue, cells, and bodily fluids [37]. Importantly, it is a stable and efficient technique for relative quantification of a large number of targets with high reproducibility and low relative standard deviation [38–40]. After completing signature peptide selection and biomarker prioritization, immunoassays or synthetic proteins/peptides could serve as alternative strategies for absolute quantification of targets for determination of cutoff values for clinical purses, in this case, BC diagnosis.

Methodological verification experiments established that 29 of 122 targets (24%) could be detected in clinical urine specimens (Table 2), a relatively low percentage that likely reflects the low concentration of these proteins in urine or their inherent lack of secretory properties. Although this detection percentage is less than ideal, these MRM-MS data proved invaluable in prioritizing the detected protein biomarkers for selection of biomarker candidates for further verification using ELISAs. Therefore, we focused only on proteins that were detectable in BC urine by MRM-MS for further verification by IHC and ELISA (Table 2). Among qualified proteins shown in Table 2, TAGLN2 has been proven to be an excellent biomarker that is highly expressed in BC tissue as well as BC urine specimens [18]. The successful methodological verification of HSPE1 protein in this study again validates the MRM-based approach for biomarker prioritization for future clinical use.

The use of MRM-MS assay mainly aimed to prioritize the bladder cancer-associated tissue proteins for the following verification in urine by immuno-based methods. The urinary concentration of HSPE1 is low. Additionally, limited loading amount of total urinary proteins (1 μg) resulted in the poor sensitivity of HSPE1 detection in urine by MRM-MS. 50 μg total urine proteins was used for quantification by Western blot analysis. Therefore, HSPE1 was detectable in more clinical urine specimens by Western blotting. ELISA was used for the final high throughput measurement of HSPE1 in individual samples. According to the result of ELISA assay (*n* = 126 for bladder cancer), HSPE1 was detectable in 97 bladder cancer urine specimens. And HSPE1 was up-expressed (cut-off value = 0.07414 ng/ml) in 95 out of 126 (75.4%) bladder cancer urine samples.

Most heat shock proteins (HSPs) function as intracellular chaperones that assure correct protein folding by assisting in the refolding and degradation of misfolded proteins, and preventing the formation of abnormal protein aggregation [41–43]. *HSPE1* (heat shock protein family E member 1), also known as HSP10 (heat shock protein 10) and CPN10 (chaperonin 10) [44, 45] encodes the protein, HSPE1. HSP60 and HSPE1 are the main constitutive components of the mitochondrial chaperonin complex”. and assist in protein folding in mitochondria [46, 47]. There is also evidence that HSPE1 may play different roles in tumor cells, with some previous studies reporting that differential expression of HSPE1 contributes to tumor survival, tumor progression, and suppression of antitumor immunity [48–51]. In this study, immunohistochemical analyses showed that HSPE1 is overexpressed in BC compared with adjacent normal tissue; similar overexpression of HSPE1 in tumor cells has also been reported in other cancers [52–55]. An alternative name for HSPE1 is early pregnancy factor (EPF) [56], which has been reported as an extracellular protein in sera of different mammals during fertilization [57–59]. In humans, extracellular HSPE1 has been reported to act as a suppressor of antitumor immunity in ovarian cancer [51]. Here, we found that urinary expression of HSPE1, which can also be measured in human blood [56, 60, 61], was greater in BC patients than in other non-cancer control conditions, including hernia, hematuria, and UTI (Figure 2B). In previous studies, increased concentrations of cellular HSPs were found to correlate with aggregation of cancer oncoproteins [62, 63], increasing research interest in HSPs for targeted cancer therapy [64–66]. The expression of other HSPs, including HSP27, HSP70 and HSP90, is correlated with BC progression [43, 67–72]. Notably, these same HSPs have been detected in BC urine specimens. However, although their levels in BC urine are significantly higher than in healthy controls, they are not significantly different compared with hematuria or non-muscle–invasive BC [73]. Current non-invasive BC diagnosis assays, including the FDA-approved urinary biomarkers [1–3] may be interfered by the hematuria and result in false-positive diagnosis. Notably, our ELISA result revealed that the level of HSPE1 in BC urine specimens was higher than hernia, hematuria and UTI, which may be able to overcome the interferences of hematuria. Notably, this observation resolves the interfering effect of hematuria that affects most BC urinary biomarkers, including other FDA-approved urinary biomarkers [74–76]. Therefore, HSPE1 is a potential indicator of BC, even in patients with hematuria.

In conclusion, we have successfully integrated tissue and urine proteomes using targeted approaches and verified tumor-specific biomarker candidates in clinical urine specimens. Specifically, HSPE1 was methodologically verified as a potential BC biomarker for early detection and diagnosis that is free from interference of hematuria, making this candidate worthy of further verification studies in a larger number of clinical specimens.

## MATERIALS AND METHODS

### Preparation of clinical urinary specimens and BC cell extracts

Urine specimens were collected from all patients and controls on the morning of surgery using a previously described protocol [24]. Hernia patients without urological system pathologies were used non-cancer disease controls. Briefly, after collecting the first morning urine samples, a protease inhibitor cocktail (one tablet/50 ml of urine; Roche, Mannheim, Germany) and sodium azide (1 mM) were added, and the collected urine specimens were centrifuged at 5000 × g for 30 min at 4°C to remove cells and debris. The supernatants were subsequently collected and stored at −80°C within 5 h of urine collection. Urinary proteins were prepared for protein quantification by targeted MS by first concentrating urinary proteins using a 10-kDa centrifugal filter (Merck Millipore Inc., USA) at 5000 × g for 30 min at 4°C, after which they were desalted and re-centrifuged with 12.5 ml of 20% acetonitrile in H_2_O, and finally desalted with 12.5 ml of water. The final volume of the concentrated urinary protein fraction was approximately 500 μl. The bladder cancer cell lines, 5637, BFTC905 and TSGH 8301, were purchased from the Food Industry Research and Development Institute (FIRDI; Taiwan). Whole-cell lysates were prepared using lysis buffer (#9803S; Cell Signaling Technology, USA), as described by the manufacturer. The amount of protein in each concentrated urine fraction and cell extracts was measured using a Pierce BCA protein assay kit (Thermo Scientific). Purified proteins were then lyophilized and stored at −20°C for subsequent processing.

### Tryptic digestion of urinary proteins and bladder cancer cell extracts

for tryptic digestion, 15 μg of urinary protein or cancer cell extract was dissolved in 96.4 μl of 50 mM sodium bicarbonate, to which 15 μl of 10% deoxycholic acid was added (final concentration, 1%). The sample was reduced by adding 12.4 μl of 50 mM Tris(2-carboxyethyl)phosphine (TCEP; final concentration, 5 mM) and incubating at 60°C for 30 min. The sample was then alkylated by adding 13.75 μl of 200 mM iodoacetamide (final concentration, 10 mM) and incubating at room temperature in the dark for 30 min. Sequencing-grade modified trypsin (Promega, Madison, WI, USA) was then added at a 1:20 (enzyme/substrate) ratio, and the samples were incubated at 37°C for 9 h. Tryptic peptides were stored at −20°C for subsequent dimethyl labeling.

### Dimethyl labeling of N-termini and lysine residues of tryptic peptides

Tryptic peptide samples (25 μg in 100 μl of 0.1 M triethylammonium bicarbonate) were mixed with 7.5 μl of freshly prepared sodium cyanoborohydride (2 M). After the addition of 4 μl of 4% (w/w in H_2_O) ^12^CH_2_-formaldehyde (light labeling) or ^13^CD_2_-formaldehyde (heavy labeling) solution, the mixtures were allowed to react for 1 h at 37°C. The reaction was quenched by adding 4 μl of 1 M ammonium bicarbonate. This procedure converted all primary amines (N-termini and side chains of lysine residues) in a tryptic peptide mixture to dimethylamines with isotopic dimethyl tags. The mass shifts per labeling site were +28 Da and +34 Da for light peptides and heavy peptides, respectively. Next, dimethylated peptide samples were desalted using an Oasis HLB 96-well μElution plate (Waters, Milford, MA, USA). The elution process was as follows: (1) activation: 200 μl acetonitrile (ACN) was added twice to each well and eluted; (2) equilibrium: 200 μl ultrapure water was added to each well (twice); (3) sample loading: 200 μl of acidified sample was added to each well and eluted; (4) first washing: 800 μl of 0.5% trifluoroacetic acid in 0.1% formic acid solution was added to each well and eluted; (5) second washing: 200 μl of water was added to each well and eluted; and (6) elution: 25 μl of 70% ACN was added and eluted (twice). Eluted peptides were lyophilized and then dissolved in 0.1% formic acid for MRM-MS analysis.

### Development of MRM methods for quantifying dimethylated peptides

targeted dimethylated peptides corresponding to biomarker candidate proteins were determined using MRMPilot software (version 2.1; AB Sciex) according to the following criteria: (1) +2 or +3 charge states; (2) peptide length, 7−20 amino acids; (3) no Met or Cys in the peptide sequence; (4) no missed cleavage sites; (5) dimethylated (K) and dimethylated (N-term) for light labeling; dimethylated-D_4_^13^C_2_ (K) and dimethylated-D_4_^13^C_2_ (K) (N-term) for heavy labeling; and (6) both Q3 and Q1 ions were smaller than 1000 Da. The Q1/Q3 transition was further confirmed by performing protein database searches using MS/MS spectra triggered by qualified Q1/Q3 transitions. The sequences of MS/MS spectra and retention times were confirmed using the Mascot Daemon database search engine (version 2.2.04; Matrix Science, London, U.K.). The uniqueness of each peptide, defined as no shared amino acid sequence with other proteins, was confirmed by database searching in UniProt. Peptides for use as internal standards (purity, >40%) were synthesized by Kelowna International Scientific (Taipei, Taiwan). Three Q1/Q3 transitions for each peptide were monitored by MRM-MS.

### LC−MRM/MS analysis and data acquisition

A nanoACQUITY UPLC System (Waters) was used for processing of dimethylated peptides. The LC−MRM/MS analysis of each sample took 70 min per run. Four microliters of each sample, representing 1 μg light-labeled endogenous peptide and 122 heavy-labeled synthetic peptide standard mixtures (20 fmol/peptide), were trapped onto a resolving analytical column (ACQUITY UPLC BEH130 C_18_, 100 μm × 100 mm, 1.7-μm particle size; Waters) at a flow rate of 600 nl/min in solvent A (0.1% formic acid in H_2_O) for 15 min. Samples were then separated using a flow rate of 400 nl/min with a 0.5-min linear gradient from 3% to 10% solvent B (0.1% formic acid in acetonitrile), then a 31.5-min linear gradient from 10% to 30% solvent B, followed by a 10-min linear gradient from 30% to 40% solvent B, and finally a 1-min linear gradient from 40% to 97% solvent B. The analytical column was then reconditioned by holding solvent B at 97% for 10 min prior to ramping back down to 3% solvent B over 1 min and re-equilibrating for 16 min with 3% solvent B. A blank solvent injection (70-min analysis with the same LC gradient used for clinical samples; flow rate, 400 nl/min) was run between samples to prevent sample carryover on the HPLC column.

An AB/MDS Sciex 5500 QTRAP with a nanoelectrospray ionization source controlled by Analyst 1.5.1 software (AB Sciex) was used for all LC-MRM/MS analyses. All acquisition methods used the following parameters: ion spray voltage, 1900−2000 V; curtain gas setting, 20 psi (ultra-high purity nitrogen); interface heater temperature, 150°C; and MS operating pressure, 3.5 × 10^−5^ Torr; Windows for Q1 and Q3 ions were set to unit resolution (0.6−0.8 Da full width at half height). MRM acquisition methods were constructed using three MRM ion pairs per peptide with fragment-ion-specific tuned CE voltages and retention time constraints. A default collision cell exit potential of 35 V was used for all MRM ion pairs, and the scheduled MRM option was used for all data acquisition, with a target cycle time of 1 s and a 4-min MRM detection window to reduce cycle time and acquire more data points per peak.

### Processing of MRM-MS Data

The 122 heavy dimethyl-labeled synthetic peptides were spiked into 119 light dimethyl-labeled individual urine peptide samples (20 fmol/peptide) for quantitative analysis by targeted MS. Detection ability in schedule-MRM mode was maximized by dividing the 122 peptides into two MRM-MS methods (61 peptides/method). MS raw data files were imported into Skyline (version 3.6) for peptide quantification using the ProteoWizard Data Access Library [77, 78]. MRM data for each peptide were processed with Skyline to generate extracted ion chromatograms representing the fragment ion signals, which were then manually inspected. The limit for differences in retention time between endogenous dimethylated peptides and heavy-dimethylated peptide standards was less than 0.1 min. Consistency in the peak-area proportion for three transitions of each peptide was checked manually to confirm that endogenous peptide signals were correct. The area ratio of undetectable endogenous peptides was set as the zero value. Processed MRM data containing peak areas, area ratio of each transition, and peak heights were exported as customized report tables using Skyline for subsequent statistical analyses.

### Western blotting

Urinary proteins (50 μg) from individuals or pooled samples were resolved by sodium dodecyl sulfate-polyacrylamide gel electrophoresis (SDS-PAGE) and transferred electrophoretically to a PVDF (polyvinylidene difluoride) membrane (Bio-Rad Laboratories) for protein quantification. The membrane was blocked by incubating with 5% non-fat dried milk in Tris-buffered saline (Bio-Rad Laboratories) containing 0.1% Tween-20 (TBST; Sigma, St. Louis, MO, USA) for 1 h at room temperature. The following antibodies were used for Western blot analysis: anti-annexin a3 (Anxa3, 1:800, GTX30892; GeneTex, Irvine, CA, USA) and anti-HSPE1 (1:10000, ab108600; Abcam, UK). The membranes were probed by incubating first with primary antibody and then with horseradish peroxidase (HRP)-conjugated secondary antibody (anti-rabbit, 1:10000, sc-2077; Santa Cruz Biotechnology, Santa Cruz, CA, USA), and detected using enhanced chemiluminescence reagents according to the manufacturer's instructions (PerkinElmer, Netherlands or Millipore, MA, USA). An internal standard from a pooled urine sample was included in all Western blot analysis gels and used to normalize the intensities of target protein bands detected in different SDS-PAGE gels.

### Immunohistochemical staining

Immunohistochemical staining of 5-μm-thick consecutive sections of formalin fixed, paraffin-embedded tissue specimens was performed as previously described [79]. Antigens for HSPE1 were retrieved with Bond Epitope Retrieval Solution 1 using a Bond-Max automated immunostainer (Vision BioSystems, Melbourne, Australia) at 100°C for 20 min. Staining was performed with a specific antibody against HSPE1 (1:10000, ab108600; Abcam) at room temperature for 60 min using a standard protocol on the Bond-Max automated IHC stainer (Leica Biosystems, Global). A Bond Polymer Refine detection system (Vision BioSystems, Melbourne, Australia) was used to reduce nonspecific staining. Immunohistochemical staining was evaluated using a quantitative scoring method defined by two parameters: staining intensity (I) and percentage of positive-stained cells (P). For staining intensity, a score of 0 represents no staining, whereas scores of 1, 2 and 3 represent weak, moderate and strong staining, respectively. The final score was obtained by multiplying the intensity (I) by the percentage of positive-stained cell (P). HSPE1 protein expression levels in BC tissue and adjacent normal cells were categorized into three groups: low staining (score, 0–99), moderate staining (score, 100–199) and strong staining (score, ≥200).

### Sandwich ELISA for HSPE1 quantification in urinary proteins

Urine specimens from age-matched patients with non-cancerous urological diseases, including hernia, UTI and hematuria, were selected as controls for biomarker methodological verification in urine. The concentrations of candidate proteins in urine samples of controls and cancer patients were measured using a commercial ELISA kit (CSB-E09917h; Cusabio, Hubei, China). The urinary concentration of HSPE1 was measured according to the respective manufacturer's instructions using 100 μl of 2-fold dilutions of raw urine specimens for each well. Fluorescence was measured with a SpectraMax M5 microplate reader (Molecular Devices, Sunnyvale, CA, USA) using excitation and emission wavelengths of 355 and 460 nm, respectively.

### Possible secretory pathways of 130 targeted proteins

To predict the possible secretion pathway of the 130 bladder cancer-associated tissue proteins, we used SignalP 4.1 server [80] for prediction of the proteins with secretory signal peptide sequences. The organism group was selected as eukaryotes and default D-cutoff values were used for identifying the proteins with a signal peptide. SecretomeP 2.0 server [81, 82] was used to predict the proteins with non-classical secretion properties. The organism group was selected as mammalian for performing prediction. We also compared these 130 up-regulated bladder cancer tissue protein with the bladder cancer urinary microparticle proteome to discovery proteins that may secrete into urine through secretion of microparticle/exosome [39]. The secretome datasets [35] of two bladder cancer cell lines, U1 and U4, were also used for comparison. For the remaining tissue proteins that were also detectable in urine may be caused as a result of cell death or an unknown secretory pathway.

### Statistical analysis

The average values of light-to-heavy peak area ratios were calculated from triplicate experiments from independent digests for clinical correlation and statistical analyses. The average area ratio of each protein target was imported into GraphPad Prism 5.0 (GraphPad Software, Inc.) for drawing box plots and performing nonparametric Mann-Whitney tests and parametric paired-*T* tests. ROC and AUC analyses of ELISA results were used to detect the optimal cutoff point that yielded the highest total accuracy with respect to discriminating different clinical classifications. The Chi-square test was employed to identify significant clinicopathological characteristics in clinical tissue specimens of BC patients. A *p*-value < 0.05 was considered statistically significant for all tests; for statistical analyses of ROC and AUC results, *p*-values were two-tailed.

## SUPPLEMENTARY MATERIALS FIGURES AND TABLES












