# Supplementary material for: Systematic verification of bladder cancer-associated tissue protein biomarker candidates in clinical urine specimens
Source: Oncotarget. 2018 Jul 20;9(56):30731–47. doi: 10.18632/oncotarget.24578 (PMC6089400; doi:10.18632/oncotarget.24578)
Supplement: Supplementary file 1 [file oncotarget-09-30731-s001.pdf]

Systematic verification of bladder cancer-associated tissue protein biomarker candidates in clinical urine specimens

SUPPLEMENTARY MATERIALS

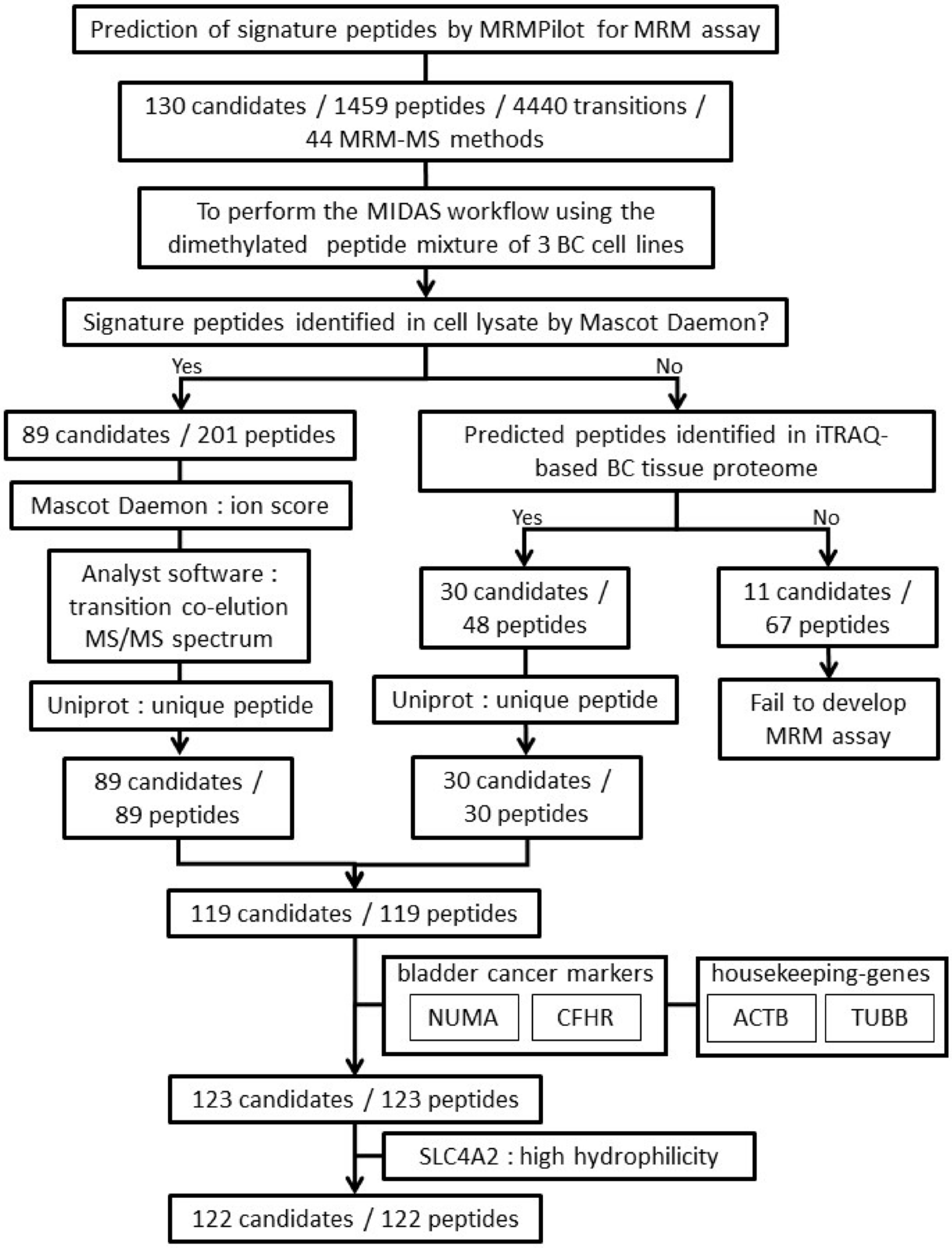

Supplementary Figure 1: Criteria for signature peptide selection in MRM prescreens of 122 BC tissue candidates. Signature peptide candidates were predicted by MRMPilot software and screened against BC cell lysate proteins using MIDAS workflow.

A Mascot Daemon

MS / MS spectrum

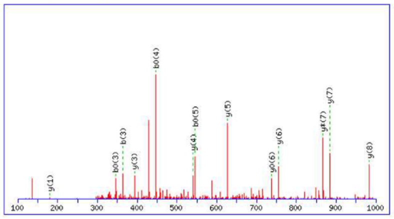


```
Monoisotopic mass of neutral peptide Mr(calc): 1446.8606
Variable modifications:
N-term : Dimethyl:2H(4)13C(2) (N-term)
K12    : Dimethyl:2H(4)13C(2) (K)
Ions Score: 42  Expect: 0.0027
Matches (Bold Red): 13/114 fragment ions using 31 most intense peaks
```

Detected product ions

#	b	b^{++}	b^{*}	b^{*++}	b^{0}	b^{0++}	Seq.	y	y^{++}	y^{*}	y^{*++}	y^{0}	y^{0++}	#
1	164.1130	82.5601			146.1024	73.5548	E							12
2	277.1970	139.1022			259.1865	130.0969	L	1284.7622	642.8847	1267.7356	634.3715	1266.7516	633.8795	11
3	364.2291	182.6182			346.2185	173.6129	S	1171.6781	586.3427	1154.6516	577.8294	1153.6676	577.3374	10
4	465.2767	233.1420			447.2662	224.1367	T	1084.6461	542.8267	1067.6195	534.3134	1066.6355	533.8214	9
5	564.3452	282.6762			546.3346	273.6709	V	983.5984	492.3028	966.5719	483.7896	965.5879	483.2976	8
6	692.4037	346.7055	675.3772	338.1922	674.3932	337.7002	Q	884.5300	442.7686	867.5035	434.2554	866.5194	433.7634	7
7	821.4463	411.2268	804.4198	402.7135	803.4358	402.2215	E	756.4714	378.7394	739.4449	370.2261	738.4609	369.7341	6
8	908.4784	454.7428	891.4518	446.2295	890.4678	445.7375	S	627.4288	314.2181	610.4023	305.7048	609.4183	305.2128	5
9	1055.5468	528.2770	1038.5202	519.7638	1037.5362	519.2717	F	540.3968	270.7020	523.3703	262.1888			4
10	1168.6308	584.8191	1151.6043	576.3058	1150.6203	575.8138	L	393.3284	197.1678	376.3018	188.6546			3
11	1267.6993	634.3533	1250.6727	625.8400	1249.6887	625.3480	V	280.2443	140.6258	263.2178	132.1125			2
12							K	181.1759	91.0916	164.1494	82.5783			1

B Analyst software

Protein name : Alpha-2-macroglobulin-like protein 1
Q1 (m/z) : 724.44 (charge : 2^{+})
Gene name : A2ML1
Modification : Heavy dimethylation (+72 Da)
Peptide sequence : [$^{13}C_2D_4H_2$]-ELSTVQESFLVK[$^{13}C_2D_4H_2$]

Q3 transition signal

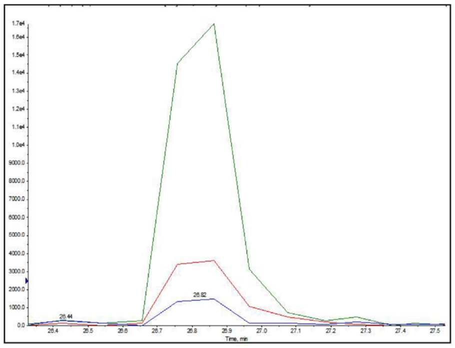

MS / MS spectrum

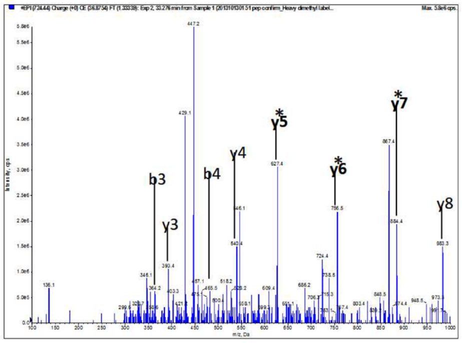

* : Q3 transition was selected for MRM assay development.

Supplementary Figure 2: An example of MS/MS spectrum confirmation by Mascot Daemon and Analyst software. (**A**) The ion score of the MS/MS spectrum in Mascot Daemon, and (**B**) co-elution of product ions in Analyst software, were further confirmed from the raw data of MRM Q3 signal and MS/MS spectrum. The intensity distributions of the top three product ions were further confirmed by MRM-MS.

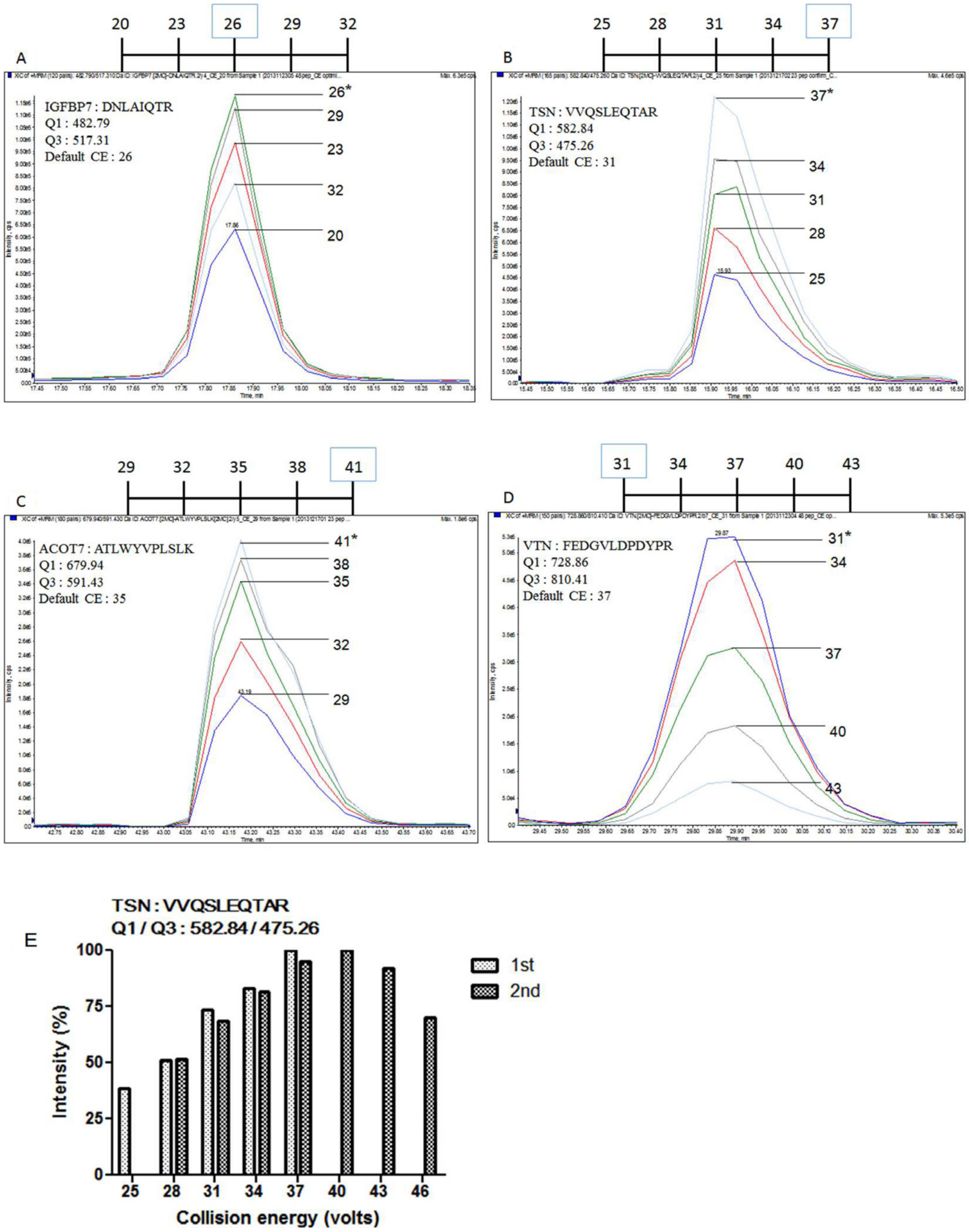

Supplementary Figure 3: Selecting the best CE using the CE ramping mode in a QTRAP MS. The Q3 signal of (**A**) IGFBP7, (**B**) TSN, (**C**) ACOT7 and (**D**) VTN were triggered with different CEs, expressed as volts. Asterisks and blue squares, optimized CE values that generate the highest Q3 ion intensity. (**E**) The signal intensity of TSN (Q1/Q3: 582.84/475.26) at different CE values. Comparison of Q3 ion intensities generated using different CE values. The ramping process was performed twice using difference CE ranges to optimize CE value for the TSN peptide.

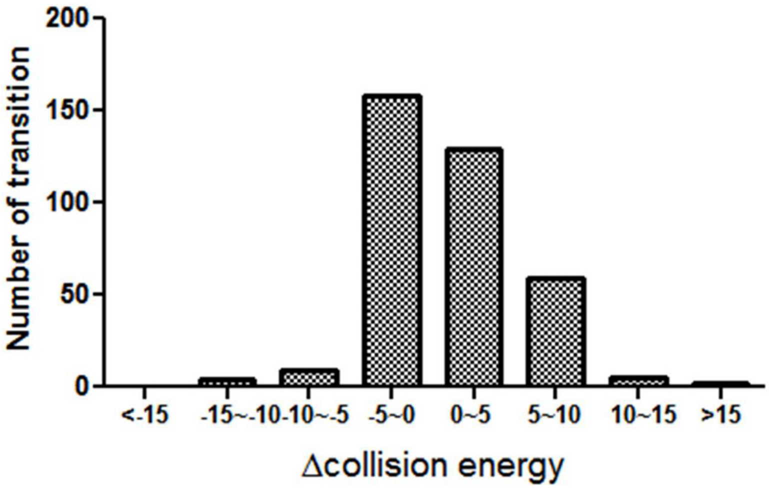

Supplementary Figure 4: Comparison of differences in CE between default and optimized value (ΔCE) for all optimized Q1/Q3 transitions. The average ΔCE was 2.09 ± 4.62 V.

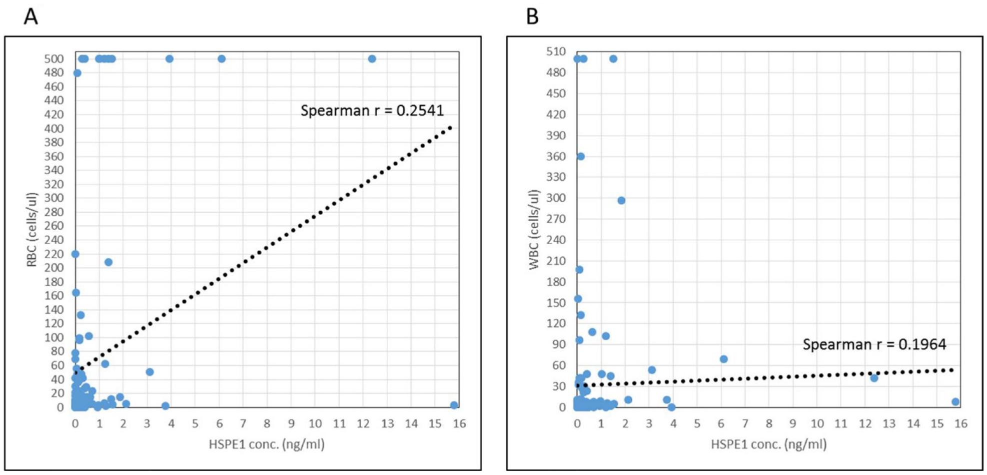

Supplementary Figure 5: Correlation analysis between urinary HSPEL levels with urine (**A**) RBC and (**B**) WBC numbers in BC patients (n = 119).

Supplementary Table 1: The list of 130 protein biomarker candidates for MRM assay development and their secretory properties. See Supplementary_Table_1

Supplementary Table 2: Criteria of peptide selection for MRM assay development. See Supplementary_Table_2

Supplementary Table 3: The transition list of MRM assay of 122 bladder cancer associated tissue proteins. See Supplementary_Table_3

Supplementary Table 4: The CV values for quantifying 122 bladder cancer associated tissue proteins in clinical urine specimens. See Supplementary_Table_4

Supplementary Table 5: Quantitative results of 12 detectable bladder cancer associated tissue proteins by MRM assay in clinical urine specimens

Targets		Bladder cancer (*n* = 30) v.s. Hernia (*n* = 31)	Bladder cancer (*n* = 30) v.s. Hematuria (*n* = 30)	Bladder cancer (*n* = 30) v.s. Urinary tract infection (*n* = 28)
ACTB	Fold change	1.017	0.867	0.355
	p-value	0.983	0.409	0.007
GAA	Fold change	0.426	0.807	1.520
	p-value	0.142	0.215	0.391
HSP90AB1	Fold change	2.523	1.124	0.763
	p-value	0.159	0.981	0.225
LAMP2	Fold change	0.445	0.402	0.969
	p-value	0.058	0.004	0.832
RAB11B	Fold change	1.124	1.346	0.511
	p-value	0.918	0.383	0.489
TPI1	Fold change	1.339	1.960	0.846
	p-value	0.873	0.401	0.987
VTN	Fold change	0.410	0.751	0.783
	p-value	0.182	0.588	0.260
DPP7	Fold change	0.595	0.835	1.172
	p-value	0.042	0.836	0.699
ENO1	Fold change	Cancer only	0.918	0.254
	p-value	N/A	0.352	0.065
IGFBP7	Fold change	0.621	1.694	3.507
	p-value	0.894	0.548	0.035
RNASET2	Fold change	0.474	0.406	0.274
	p-value	0.001	<0.0001	<0.0001
SERPINA3	Fold change	1.013	0.689	0.556
	p-value	0.493	0.039	0.234

Supplementary Table 6: Correlation results between urinary HSPE1 expression and clinicopathological characteristics of the bladder cancer patients by a Chi-squared test

Characteristics	Urinary HSPE1 Conc. (ng/ml)		Total number	*p* values
	Low	High		
	<0.074 g/mL	>0.074 ng/mL		
**Age**				
≤65 years	16	47	63	0.960
>65 years	15	45	60	
**Sex**				
Male	25	67	92	0.270
Female	6	28	34	
**Histologic grade**				
Low grade	10	32	42	0.884
High grade	21	63	84	
**TNM stage**				
Early stage	23	75	98	0.580
Advanced stage	8	20	28	
**Hematuria**				
RBC<20 (cells/ul)	23	58	81	0.395
RBC>20 (cells/ul)	8	30	38	
**Urinary tract infection**				
WBC<30 (cells/ul)	28	68	96	0.114
WBC>30 (cells/ul)	3	20	23	

Supplementary Table 7: The IHC scores of expressions of HSPE1 in clinical tissue slides of BC patients. See Supplementary_Table_7

REFERENCES

1. Chen CL, Lai YF, Tang P, Chien KY, Yu JS, Tsai CH, Chen HW, Wu CC, Chung T, Hsu CW, Chen CD, Chang YS, Chang PL, et al. Comparative and targeted proteomic analyses of urinary microparticles from bladder cancer and hernia patients. J Proteome Res. 2012; 11:5611–29.

2. Wu CC, Hsu CW, Chen CD, Yu CJ, Chang KP, Tai DI, Liu HP, Su WH, Chang YS, Yu JS. Candidate serological biomarkers for cancer identified from the secretomes of 23 cancer cell lines and the human protein atlas. Mol Cell Proteomics. 2010; 9:1100–17.
