# Supplementary material for: Systematic verification of bladder cancer-associated tissue protein biomarker candidates in clinical urine specimens
Source: Oncotarget. 2018 Jul 20;9(56):30731–47. doi: 10.18632/oncotarget.24578 (PMC6089400; doi:10.18632/oncotarget.24578)
Supplement: Supplementary file 2 [file oncotarget-09-30731-s002.docx]

**Supplementary Table 1**: **The list of 130 protein biomarker candidates for MRM assay development and their secretory properties**

| Accession number (IPI) | Accession number (Uniprot) | Genes | Proteins | Fail to established in MRM assay : ● | Classically secreted proteins by SignalP | Non-classically secreted proteins by SecretomeP | Bladder cancer urinary microparticle  Proteome [1] | Secretome of BC cell lines [2] |
| --- | --- | --- | --- | --- | --- | --- | --- | --- |
| IPI00000051.4 | O60925 | PFDN1 | Prefoldin subunit 1 |  |  | ● |  |  |
| IPI00001589.1 | Q9Y5L4 | TIMM13 | Mitochondrial import inner membrane translocase subunit Tim13 |  |  | ● |  | ● |
| IPI00002203.6 | Q9P287-1 | BCCIP | Isoform 1 of BRCA2 and CDKN1A-interacting protein |  |  | ● |  | ● |
| IPI00002857.2 | Q16539-1 | MAPK14 | Isoform CSBP2 of Mitogen-activated protein kinase 14 |  |  |  | ● | ● |
| IPI00003870.1 | Q16740 | CLPP | Putative ATP-dependent Clp protease proteolytic subunit, mitochondrial | ● |  |  |  | ● |
| IPI00004797.1 | P54107-1 | CRISP1 | Isoform Long of Cysteine-rich secretory protein 1 | ● | ● |  |  |  |
| IPI00005102.3 | P52788-1 | SMS | Isoform 1 of Spermine synthase |  |  |  | ● | ● |
| IPI00005563.1 | Q9GZM7-1 | TINAGL1 | Isoform 1 of Tubulointerstitial nephritis antigen-like |  | ● | ● |  | ● |
| IPI00005585.5 | O14907 | TAX1BP3 | Tax1-binding protein 3 |  |  | ● |  |  |
| IPI00005658.3 | P11441 | UBL4A | Ubiquitin-like protein 4A |  |  | ● |  | ● |
| IPI00005861.1 | O43395 | PRPF3 | U4/U6 small nuclear ribonucleoprotein Prp3 |  |  | ● |  |  |
| IPI00006211.4 | O95292-1 | VAPB | Isoform 1 of Vesicle-associated membrane protein-associated protein B/C |  |  |  |  | ● |
| IPI00007321.2 | B4DJV9 | LYPLA1 | cDNA FLJ60607, highly similar to Acyl-protein thioesterase 1 |  |  |  |  | ● |
| IPI00008164.2 | P48147 | PREP | Prolyl endopeptidase |  |  |  | ● | ● |
| IPI00008454.1 | Q9UBS4 | DNAJB11 | DnaJ homolog subfamily B member 11 |  | ● | ● |  | ● |
| IPI00008561.1 | P03956 | MMP1 | Interstitial collagenase |  | ● | ● |  |  |
| IPI00008599.3 | Q15125 | EBP | 3-beta-hydroxysteroid-Delta(8),Delta(7)-isomerase |  |  |  |  |  |
| IPI00008943.3 | Q9UMR2-1 | DDX19B | Isoform 1 of ATP-dependent RNA helicase DDX19B |  |  |  | ● | ● |
| IPI00009030.1 | P13473-1 | LAMP2 | Isoform LAMP-2A of Lysosome-associated membrane glycoprotein 2 |  |  | ● | ● |  |
| IPI00009504.2 | Q08357 | SLC20A2 | Sodium-dependent phosphate transporter 2 |  |  |  |  |  |
| IPI00010415.2 | O00154-1 | ACOT7 | Isoform 1 of Cytosolic acyl coenzyme A thioester hydrolase |  |  | ● |  | ● |
| IPI00011284.1 | P21964-1 | COMT | Isoform Membrane-bound of Catechol O-methyltransferase |  |  | ● | ● |  |
| IPI00011619.4 | O43252 | PAPSS1 | Bifunctional 3'-phosphoadenosine 5'-phosphosulfate synthase 1 |  |  |  | ● |  |
| IPI00011996.5 | Q9H832-1 | UBE2Z | Isoform 1 of Ubiquitin-conjugating enzyme E2 Z | ● |  |  | ● | ● |
| IPI00012007.6 | P23526 | AHCY | Adenosylhomocysteinase |  |  |  | ● | ● |
| IPI00012093.1 | O00506 | STK25 | Serine/threonine-protein kinase 25 |  |  |  | ● |  |
| IPI00012197.1 | Q9H773 | DCTPP1 | dCTP pyrophosphatase 1 |  |  | ● |  | ● |
| IPI00012578.1 | O00629 | KPNA4 | Importin subunit alpha-4 |  |  |  | ● | ● |
| IPI00013159.1 | P41212 | ETV6 | Transcription factor ETV6 | ● |  |  |  |  |
| IPI00013890.2 | P31947-1 | SFN | Isoform 1 of 14-3-3 protein sigma |  |  |  | ● | ● |
| IPI00014587.1 | P09496-1 | CLTA | Isoform Brain of Clathrin light chain A |  |  | ● |  | ● |
| IPI00014938.3 | P82979 | SARNP | SAP domain-containing ribonucleoprotein |  |  |  |  | ● |
| IPI00015891.1 | Q9NQP4 | PFDN4 | Prefoldin subunit 4 |  |  | ● |  | ● |
| IPI00016572.1 | P62308 | SNRPG^#^ | Small nuclear ribonucleoprotein G | ● |  | ● |  |  |
| IPI00016915.1 | Q16270 | IGFBP7 | Insulin-like growth factor-binding protein 7 |  | ● |  | ● | ● |
| IPI00018146.1 | P27348 | YWHAQ | 14-3-3 protein theta |  |  |  | ● | ● |
| IPI00018274.1 | P00533-1 | EGFR | Isoform 1 of Epidermal growth factor receptor |  | ● |  | ● | ● |
| IPI00018768.1 | Q15631 | TSN | Translin |  |  |  | ● | ● |
| IPI00018804.3 | Q15642-2 | TRIP10 | Isoform 2 of Cdc42-interacting protein 4 |  |  |  | ● |  |
| IPI00019178.2 | P78330 | PSPH | Phosphoserine phosphatase |  |  |  | ● | ● |
| IPI00020004.3 | Q5BJF2 | TMEM97 | Transmembrane protein 97 |  |  |  |  |  |
| IPI00020436.4 | Q15907 | RAB11B | Ras-related protein Rab-11B |  |  |  | ● | ● |
| IPI00021187.4 | Q9Y265-1 | RUVBL1 | Isoform 1 of RuvB-like 1 |  |  |  | ● | ● |
| IPI00022078.3 | Q92597 | NDRG1 | Protein NDRG1 |  |  | ● | ● | ● |
| IPI00022462.2 | P02786 | TFRC | Transferrin receptor protein 1 |  |  | ● | ● | ● |
| IPI00022892.2 | P04216 | THY1 | Thy-1 membrane glycoprotein |  | ● |  | ● |  |
| IPI00023728.1 | Q92820 | GGH | Gamma-glutamyl hydrolase |  | ● | ● | ● | ● |
| IPI00024095.3 | P12429 | ANXA3 | Annexin A3 |  |  |  | ● | ● |
| IPI00024290.1 | O14896 | IRF6 | Interferon regulatory factor 6 |  |  | ● | ● | ● |
| IPI00024913.2 | P30042-1 | C21orf33 | Isoform Long of ES1 protein homolog, mitochondrial |  | ● | ● |  | ● |
| IPI00024976.5 | Q9NS69 | TOMM22 | Mitochondrial import receptor subunit TOM22 homolog | ● |  |  |  |  |
| IPI00025273.1 | P22102-1 | GART | Isoform Long of Trifunctional purine biosynthetic protein adenosine-3 |  |  |  | ● | ● |
| IPI00026833.4 | P30520 | ADSS | Adenylosuccinate synthetase isozyme 2 |  |  |  | ● | ● |
| IPI00027493.1 | P08195-2 | SLC3A2 | Isoform 2 of 4F2 cell-surface antigen heavy chain |  |  | ● | ● | ● |
| IPI00027851.2 | B4DVA7 | HEXA | cDNA FLJ53927, highly similar to Beta-hexosaminidase alpha chain |  | ● | ● |  | ● |
| IPI00028109.1 | Q9C005 | DPY30 | Protein dpy-30 homolog |  |  | ● |  |  |
| IPI00028376.1 | O60220 | TIMM8A | Mitochondrial import inner membrane translocase subunit Tim8 A |  |  | ● |  |  |
| IPI00031008.1 | P24821-1 | TNC | human tenascin-C |  | ● |  | ● | ● |
| IPI00031030.1 | Q06481-1 | APLP2 | Isoform 1 of Amyloid-like protein 2 |  | ● |  | ● | ● |
| IPI00031570.1 | Q9BSD7 | NTPCR | Cancer-related nucleoside-triphosphatase |  |  |  |  | ● |
| IPI00032140.4 | P50454 | SERPINH1 | Serpin H1 |  | ● | ● |  | ● |
| IPI00037283.3 | O00429-5 | DNM1L | Isoform 5 of Dynamin-1-like protein |  |  |  | ● | ● |
| IPI00065500.3 | Q5VW32 | BROX | BRO1 domain-containing protein BROX |  |  |  | ● | ● |
| IPI00075248.11 | P62158 | CALM3 | Calmodulin |  |  | ● | ● | ● |
| IPI00169383.3 | P00558 | PGK1 | Phosphoglycerate kinase 1 |  |  |  | ● | ● |
| IPI00170796.1 | Q9UBQ0-1 | VPS29 | Isoform 1 of Vacuolar protein sorting-associated protein 29 |  |  |  | ● | ● |
| IPI00178188.5 | Q8TF09 | DYNLRB2 | Dynein light chain roadblock-type 2 |  |  | ● |  |  |
| IPI00180292.5 | Q9UQB8-5 | BAIAP2 | Isoform 5 of Brain-specific angiogenesis inhibitor 1-associated protein 2 |  |  |  | ● | ● |
| IPI00185919.3 | Q6PKG0-1 | LARP1 | Isoform 1 of La-related protein 1 |  |  |  |  | ● |
| IPI00215918.3 | P18085 | ARF4 | ADP-ribosylation factor 4 |  |  | ● | ● | ● |
| IPI00216164.4 | Q08426 | EHHADH | Peroxisomal bifunctional enzyme |  |  |  |  |  |
| IPI00216298.6 | P10599 | TXN | Thioredoxin |  |  |  | ● | ● |
| IPI00216694.3 | P13797 | PLS3 | Plastin-3 |  |  |  | ● | ● |
| IPI00217272.7 | Q86V21-1 | AACS | Isoform 1 of Acetoacetyl-CoA synthetase |  |  |  | ● |  |
| IPI00217918.1 | Q8IZ81 | ELMOD2 | ELMO domain-containing protein 2 |  |  |  |  |  |
| IPI00218414.5 | P00918 | CA2 | Carbonic anhydrase 2 |  |  |  | ● | ● |
| IPI00218493.7 | P00492 | HPRT1 | Hypoxanthine-guanine phosphoribosyltransferase |  |  | ● | ● | ● |
| IPI00218803.3 | P23142-3 | FBLN1 | Isoform B of Fibulin-1 |  | ● |  | ● | ● |
| IPI00218839.2 | P04798 | CYP1A1 | Cytochrome P450 1A1 |  |  | ● |  |  |
| IPI00220362.5 | P61604 | HSPE1 | 10 kDa heat shock protein, mitochondrial |  |  |  |  | ● |
| IPI00220766.5 | Q04760-1 | GLO1 | Isoform 1 of Lactoylglutathione lyase |  |  |  | ● | ● |
| IPI00291200.2 | Q8WUM0 | NUP133 | Nuclear pore complex protein Nup133 | ● |  |  |  |  |
| IPI00291510.3 | P12268 | IMPDH2 | Inosine-5'-monophosphate dehydrogenase 2 |  |  |  | ● | ● |
| IPI00293307.1 | Q99541 | PLIN2 | Perilipin-2 |  |  |  |  |  |
| IPI00293867.7 | P30046 | DDT | D-dopachrome decarboxylase |  |  |  | ● | ● |
| IPI00294619.2 | Q92734 | TFG | Protein TFG |  |  |  | ● | ● |
| IPI00296141.4 | Q9UHL4 | DPP7 | Dipeptidyl peptidase 2 |  | ● | ● | ● | ● |
| IPI00296537.4 | P23142-4 | FBLN1 | Isoform C of Fibulin-1 |  | ● |  | ● | ● |
| IPI00296913.1 | Q9UKK9 | NUDT5 | ADP-sugar pyrophosphatase |  |  |  | ● | ● |
| IPI00298971.1 | P04004 | VTN | Vitronectin |  | ● | ● | ● | ● |
| IPI00300094.6 | Q9H089 | LSG1 | Large subunit GTPase 1 homolog |  |  |  |  |  |
| IPI00301280.2 | Q9BTV4 | TMEM43 | Transmembrane protein 43 |  |  | ● |  |  |
| IPI00302944.3 | Q99715-4 | COL12A1 | Isoform 4 of Collagen alpha-1(XII) chain |  | ● |  |  |  |
| IPI00306322.2 | P08572 | COL4A2 | Collagen alpha-2(IV) chain |  | ● |  |  |  |
| IPI00328867.6 | P12931-2 | SRC | Isoform 2 of Proto-oncogene tyrosine-protein kinase Src |  |  |  | ● | ● |
| IPI00334907.3 | P48739-1 | PITPNB | Isoform 1 of Phosphatidylinositol transfer protein beta isoform |  |  |  | ● | ● |
| IPI00374563.3 | O00468 | AGRN | Agrin |  | ● |  | ● | ● |
| IPI00375631.6 | P05161 | ISG15 | Ubiquitin-like protein ISG15 |  |  | ● | ● | ● |
| IPI00383597.2 | P04920-1 | SLC4A2 | Isoform A of Anion exchange protein 2 | ● |  |  |  |  |
| IPI00395663.5 | Q92625 | ANKS1A | Ankyrin repeat and SAM domain-containing protein 1A |  |  |  | ● |  |
| IPI00399183.5 | Q0VD83-1 | APOBR | Apolipoprotein B receptor |  |  |  |  |  |
| IPI00413778.7 | Q0VDC6 | FKBP1A | FKBP1A protein | ● |  | ● |  |  |
| IPI00414676.6 | P08238 | HSP90AB1 | Heat shock protein HSP 90-beta |  |  |  | ● |  |
| IPI00414896.1 | O00584-1 | RNASET2 | Isoform 1 of Ribonuclease T2 |  | ● | ● | ● | ● |
| IPI00418433.1 | P49327 | FASN | Fatty acid synthase |  |  |  | ● |  |
| IPI00419215.5 | A8K2U0 | A2ML1 | Alpha-2-macroglobulin-like protein 1 |  | ● | ● |  |  |
| IPI00419237.3 | P28838-1 | LAP3 | Isoform 1 of Cytosol aminopeptidase |  |  |  | ● | ● |
| IPI00441867.1 | P40855-1 | PEX19 | Isoform 1 of Peroxisomal biogenesis factor 19 | ● |  | ● |  |  |
| IPI00453476.2 | F2Z2J9 | LOC643576 | Phosphoglycerate mutase |  |  |  |  | ● |
| IPI00456940.5 | Q6DKI1 | RPL7L1 | 60S ribosomal protein L7-like 1 |  |  |  |  |  |
| IPI00465028.7 | P60174-3 | TPI1 | triosephosphate isomerase isoform 2 |  |  |  | ● | ● |
| IPI00465248.5 | P06733-1 | ENO1 | Isoform alpha-enolase of Alpha-enolase |  |  |  | ● | ● |
| IPI00472013.1 | P16190 | HLA-A | HLA class I histocompatibility antigen, A-33 alpha chain |  | ● |  |  |  |
| IPI00472164.2 | Q9Y6W5 | WASF2 | Wiskott-Aldrich syndrome protein family member 2 |  |  |  | ● | ● |
| IPI00472610.2 | Q6PI81 | IGHM | IGHM protein | ● |  |  |  |  |
| IPI00477729.2 | Q15067-2 | ACOX1 | Isoform 2 of Peroxisomal acyl-coenzyme A oxidase 1 |  |  | ● | ● |  |
| IPI00479997.4 | P16949-1 | STMN1 | Isoform 1 of Stathmin |  |  | ● |  | ● |
| IPI00549467.3 | Q9NQR4 | NIT2 | Omega-amidase NIT2 |  |  | ● | ● | ● |
| IPI00550363.3 | P37802 | TAGLN2 | Transgelin-2 |  |  | ● | ● | ● |
| IPI00550746.4 | Q9Y266 | NUDC | Nuclear migration protein nudC |  |  |  | ● | ● |
| IPI00551062.2 | Q9BT09-1 | CNPY3 | Isoform 1 of Protein canopy homolog 3 | ● | ● |  |  |  |
| IPI00554590.1 | Q9H2M9-1 | RAB3GAP2 | Isoform 1 of Rab3 GTPase-activating protein non-catalytic subunit |  |  |  |  | ● |
| IPI00644196.1 | P36952-2 | SERPINB5 | Isoform 2 of Serpin B5 |  |  |  | ● |  |
| IPI00783097.3 | P41250 | GARS | glycyl-tRNA synthetase, isoform CRA_b |  | ● |  | ● | ● |
| IPI00783446.1 | P10253 | GAA | glucan 1, 4-alpha-glucosidase |  | ● | ● | ● |  |
| IPI00783625.1 | P36952 | SERPINB5 | maspin |  |  |  | ● | ● |
| IPI00784154.1 | P10809 | HSPD1 | 60 kDa heat shock protein, mitochondrial |  |  |  |  |  |
| IPI00784376.2 | Q9UDX5-1 | MTFP1 | Isoform 1 of Mitochondrial fission process protein 1 |  |  | ● |  |  |
| IPI00793199.1 | Q6LES2 | ANXA4 | annexin A4 |  |  |  |  | ● |
| IPI01025667.1 | P01011 | SERPINA3 | cDNA FLJ35730 fis, clone TESTI2003131, highly similar to ALPHA-1-ANTICHYMOTRYPSIN |  | ● | ● | ● |  |
