# Supplementary material for: Systematic verification of bladder cancer-associated tissue protein biomarker candidates in clinical urine specimens
Source: Oncotarget. 2018 Jul 20;9(56):30731–47. doi: 10.18632/oncotarget.24578 (PMC6089400; doi:10.18632/oncotarget.24578)
Supplement: Supplementary file 3 [file oncotarget-09-30731-s003.docx]

**Supplementary Table 2**: **Criteria of peptide selection for MRM assay development**

| Genes (130) | Peptide sequence (1459) | Mascot score | Mascot score cutoff  (● : p<0.05 ; ▲ : p>0.05) | Mascot score cutoff  (A : p<0.05 ; B : p>0.05 ; C : detected in iTRAQ-based BC tissue proteome^1^, but identification failed in BC cell lysates ) | Unique peptide  (● : unique ; ▲ : non-unique) | Selected for MRM assay : ● |
| --- | --- | --- | --- | --- | --- | --- |
| A2ML1 | AGLLTEEIR |  |  |  |  |  |
|  | ANTYWYR |  |  |  |  |  |
|  | ASFSLSLTFTSR |  |  |  |  |  |
|  | AVDESVLLLRPDR |  |  |  |  |  |
|  | AVGFLEIGYQK |  |  |  |  |  |
|  | EAVHVTVPDAITEWK |  |  |  |  |  |
|  | ELSTVQESFLVK | 5.45 | ▲ | B | ● | ● |
|  | FTVTLETK |  |  |  |  |  |
|  | GHDDSFLK |  |  |  |  |  |
|  | HSNGSYS |  |  |  |  |  |
|  | LGHINFTISTK |  |  |  |  |  |
|  | LLEYSGLK | 18.32 | ▲ | B | ● |  |
|  | LNFPSVQK |  |  |  |  |  |
|  | LTATIFNYLK |  |  |  |  |  |
|  | NLSGQTDK |  |  |  |  |  |
|  | SFLGIHR |  |  |  |  |  |
|  | TFGTFSVEEYVLPK |  |  |  |  |  |
|  | THHWNITAVK | 4.22 | ▲ | B | ● |  |
|  | VEVVEPK |  |  |  |  |  |
|  | VQTDLAK |  |  |  |  |  |
|  | VSGVGNNISFEEK |  |  |  |  |  |
| AACS | AQSYEYVYR |  |  |  |  |  |
|  | DIPELQGF |  |  |  |  |  |
|  | ENIDLSK |  |  |  |  |  |
|  | FSGIVFSR |  |  |  |  |  |
|  | FSQIQPK |  |  |  |  |  |
|  | GIADVPEWFK |  |  | C | ● | ● |
|  | GIPYTLNGK |  |  |  |  |  |
|  | HVPSLILETK |  |  |  |  |  |
|  | IGITVLVTGAK |  |  |  |  |  |
|  | LIFSVEAVVYNGK |  |  |  |  |  |
|  | LNYAENLLR |  |  |  |  |  |
|  | SDGTLNPNGVR |  |  |  |  |  |
|  | VALYIAR |  |  |  |  |  |
|  | VTFEELR |  |  |  |  |  |
|  | VVVIPYVSSR |  |  |  |  |  |
|  | VYDEVVDTSK |  |  |  |  |  |
|  | WLSVLEEK |  |  |  |  |  |
| ACOT7 | AASAFFTYVSLSQEGR | 8 | ▲ | B | ● |  |
|  | ATLWYVPLSLK | 27 | ▲ | B | ● | ● |
|  | QEQEEEGR |  |  |  |  |  |
|  | QGHAEPQP |  |  |  |  |  |
|  | SLPVPQLVPETEDEK |  |  |  |  |  |
|  | TNIVTASVDAINFHDK |  |  |  |  |  |
|  | VLEVPPVVYSR |  |  |  |  |  |
| ACOX1 | AEVHESYK |  |  |  |  |  |
|  | AIQAVLR |  |  |  |  |  |
|  | ELLTLIR |  |  |  |  |  |
|  | EVAWNLTSVDLVR |  |  | C |  |  |
|  | FGYDEIDNGYLK |  |  |  |  |  |
|  | LVEIAAK |  |  |  |  |  |
|  | SFLVGEAAR |  |  |  |  |  |
|  | SYDQVHSGK |  |  |  |  |  |
|  | TSNHAIVLAQLITK |  |  |  |  |  |
|  | WWPGGLGK |  |  |  | ● | ● |
|  | YDGNVYENLFEWAK |  |  |  |  |  |
| ADSS | ARPGGNR |  |  |  |  |  |
|  | EFGVTTGR |  |  |  |  |  |
|  | ELPVNAQNYVR |  |  |  |  |  |
|  | FIEDELQIPVK | 18.39 | ▲ | B | ● | ● |
|  | GIGPVYSSK | 5.87 | ▲ | B | ● |  |
|  | GLEGWEK |  |  |  |  |  |
|  | SIYPTLEIDIEGELQK |  |  |  |  |  |
|  | TLPGWNTDISNAR |  |  |  |  |  |
|  | VLANQYK |  |  |  |  |  |
|  | VTVVLGAQWGDEGK |  |  |  |  |  |
| AGRN | AAAVSSGFDGAIQLVSLGGR |  |  |  |  |  |
|  | AIVDVHFDPTTAFR |  |  |  |  |  |
|  | ALEPQGLLLYNGNAR |  |  |  |  |  |
|  | ALQSNHFELSLR |  |  |  |  |  |
|  | APHPSHTSQPVAK |  |  |  |  |  |
|  | DFLALALLDGR |  |  |  |  |  |
|  | EPLYVGGAPDFSK |  |  |  |  |  |
|  | EPVTLGAWTR |  |  |  |  |  |
|  | ESLLDGGNK |  |  |  |  |  |
|  | GDFVSLALR | 6 | ▲ | B | ● | ● |
|  | GIVTDGR |  |  |  |  |  |
|  | GLHTFAR |  |  |  |  |  |
|  | GPSGLLLYNGQK |  |  |  |  |  |
|  | IFFVNPAPPYLWPAHK |  |  |  |  |  |
|  | LELGIGPGAATR |  |  |  |  |  |
|  | LLDVNNQR |  |  |  |  |  |
|  | LPSSAVTPR |  |  |  |  |  |
|  | QLLTPEHVLR |  |  |  |  |  |
|  | RPLQEHVR |  |  |  |  |  |
|  | RPPTTAPSR |  |  |  |  |  |
|  | SAGDVDTLAFDGR |  |  |  |  |  |
|  | SELFGETAR |  |  |  |  |  |
|  | SFLAFPTLR |  |  |  |  |  |
|  | SIESTLDDLFR |  |  |  |  |  |
|  | STVPVNTNR |  |  |  |  |  |
|  | TEATQGLVLWSGK |  |  |  |  |  |
|  | TFVEYLNAVTESEK |  |  |  |  |  |
|  | TFVGAGLR |  |  |  |  |  |
|  | TTAAPTTR |  |  |  |  |  |
|  | TTASVPR |  |  |  |  |  |
|  | VLGAPVPAFEGR |  |  |  |  |  |
|  | VLGESPVPHTVLNLK |  |  |  |  |  |
| AHCY | AGIPVYAWK | 18.87 | ▲ | B | ● |  |
|  | ESLIDGIK | 19.23 | ▲ | B | ● |  |
|  | GISEETTTGVHNLYK | 25.01 | ▲ | B | ● |  |
|  | IILLAEGR |  |  |  |  |  |
|  | LDEAVAEAHLGK | 0.32 | ▲ | B | ● |  |
|  | VADIGLAAWGR | 3.02 | ▲ | B | ● |  |
|  | VAVVAGYGDVGK | 24.51 | ▲ | B | ● |  |
|  | VNIKPQVDR |  |  |  |  |  |
|  | VPAINVNDSVTK | 6.84 | ▲ | B | ● |  |
|  | WLNENAVEK | 31.69 | ● | A | ● | ● |
|  | YPQLLPGIR | 6.62 | ▲ | B | ● |  |
|  | YPVGVHFLPK |  |  |  |  |  |
|  | YSASKPLK | 8.16 | ▲ | B | ● |  |
| ANKS1A | APPTSKPK | 7 | ▲ | B | ● |  |
|  | DIGISDPQHR |  |  |  |  |  |
|  | DNHGLTALDTVR |  |  |  |  |  |
|  | DVVEVLLR | 8 | ▲ | B | ● |  |
|  | EAEAAGVKPAGVRPR |  |  |  |  |  |
|  | EQELLEAAR |  |  |  |  |  |
|  | FETPLDLAALYGR | 9 | ▲ | B | ● | ● |
|  | FIDASNK |  |  |  |  |  |
|  | GDAQIVR |  |  |  |  |  |
|  | HDSLHDPAAPSR |  |  |  |  |  |
|  | HTPLHLAAR |  |  |  |  |  |
|  | IHGSAAR |  |  |  |  |  |
|  | IPTIILSITYK |  |  |  |  |  |
|  | LLIHQGPSHTR |  |  |  |  |  |
|  | NDALTNVADSK |  |  |  |  |  |
|  | NLWELELVNVLK |  |  |  |  |  |
|  | NVIAEHEIR |  |  |  |  |  |
|  | SADLLLPPGDTGR |  |  |  |  |  |
|  | SPSFASEWDEIEK |  |  |  |  |  |
|  | SQGDVEK |  |  |  |  |  |
|  | SSDQDSTNK |  |  |  |  |  |
|  | TDVVQILLAAGTDVNIK |  |  |  |  |  |
|  | TGHLPAVEK |  |  |  |  |  |
|  | VGYLTGLPTTNSR |  |  |  |  |  |
|  | VQLLGHR |  |  |  |  |  |
|  | VVLVDGK |  |  |  |  |  |
| ANXA3 | ALLTLADGR | 44.62 | ● | A | ● |  |
|  | DISQAYYTVYK | 27.85 | ▲ | B | ● |  |
|  | DIVDSIK |  |  |  |  |  |
|  | DYPDFSPSVDAEAIQK | 39.63 | ● | A | ● |  |
|  | EYQAAYGK |  |  |  |  |  |
|  | GAGTNEDALIEILTTR | 6.72 | ▲ | B | ● |  |
|  | GIGTDEFTLNR | 53.28 | ● | A | ● | ● |
|  | GIGTDEK |  |  |  |  |  |
|  | HYGYSLYSAIK | 8.24 | ▲ | B | ● |  |
|  | LTFDEYR |  |  |  |  |  |
|  | NTPAFLAER | 22.03 | ▲ | B | ● |  |
|  | QDAQILYK | 21.02 | ▲ | B | ● |  |
|  | SDTSGDYEITLLK | 11.23 | ▲ | B | ● |  |
|  | SEIDLLDIR | 21.53 | ▲ | B | ● |  |
|  | SLGDDISSETSGDFR | 37.85 | ● | A | ● |  |
|  | VDEHLAK |  |  |  |  |  |
|  | WGTDEDK |  |  |  |  |  |
| ANXA4 | DEGNYLDDALVR | 44.8 | ● | A | ● |  |
|  | DIEQSIK | 20.31 | ▲ | B | ● |  |
|  | DLIDDLK |  |  |  |  |  |
|  | GDTSGDYR |  |  |  |  |  |
|  | GLGTDEDAIISVLAYR |  |  |  |  |  |
|  | ISQTYQQQYGR | 10.56 | ▲ | B | ● |  |
|  | NHLLHVFDEYK |  |  |  |  |  |
|  | QDAQDLYEAGEK | 45.89 | ● | A | ● | ● |
|  | SAYFAEK |  |  |  |  |  |
|  | SETSGSFEDALLAIVK | 71.03 | ● | A | ● |  |
|  | SLEDDIR |  |  |  |  |  |
|  | SLYSFIK | 5.46 | ▲ | B | ● |  |
|  | VLVSLSAGGR | 8.32 | ▲ | B | ● |  |
|  | WGTDEVK |  |  |  |  |  |
| APLP2 | DYYYDTFK |  |  |  |  |  |
|  | EITHDVK |  |  |  |  |  |
|  | ESVGPLR |  |  |  |  |  |
|  | EWEEAELQAK |  |  |  |  |  |
|  | GSGVGEQDGGLIGAEEK |  |  |  |  |  |
|  | HYQHVLAVDPEK |  |  |  |  |  |
|  | IIGSVSK |  |  |  |  |  |
|  | NQSLSLLYK |  |  |  |  |  |
|  | QQLVETHLAR |  |  |  | ● | ● |
|  | VGGLEEER |  |  |  |  |  |
|  | WEPDPTGTK |  |  |  |  |  |
|  | WYFDLSK | 4 | ▲ | B | ● |  |
| APOBR | AGAVGPK |  |  |  |  |  |
|  | AILDGEEAR |  |  |  |  |  |
|  | EADAGETEEPGAEGAGK |  |  |  |  |  |
|  | EAEVSPFPK |  |  |  |  |  |
|  | EAQAAEELGVVAVGK |  |  |  |  |  |
|  | EEADLLGVR |  |  |  |  |  |
|  | EGGPWGGR |  |  |  |  |  |
|  | ETEDEEAEADR |  |  |  |  |  |
|  | ETEPESLEHVR |  |  | C |  |  |
|  | GAESEWTWHGETEGK |  |  |  |  |  |
|  | GEEVVVVEK |  |  |  |  |  |
|  | GNTQEDAADGEQR |  |  |  |  |  |
|  | GSQNEGAGR |  |  |  |  |  |
|  | GSQVEAFESR |  |  |  |  |  |
|  | HDGTPVPAR |  |  |  |  |  |
|  | IVEEEAQEDLEGLR |  |  | C |  |  |
|  | LGRPKPQ |  |  |  |  |  |
|  | LLEATGK |  |  |  |  |  |
|  | LQAVAVGLPDR |  |  |  |  |  |
|  | LYLPGLHQALR |  |  |  |  |  |
|  | QAQVLGTER |  |  |  |  |  |
|  | QDSGAGETAK |  |  |  |  |  |
|  | QESHEQEVNR |  |  |  |  |  |
|  | QTEYGAVPGER |  |  |  |  |  |
|  | SGQAQER |  |  |  |  |  |
|  | SLEAGPR |  |  |  |  |  |
|  | SWEQEEEEEEVR |  |  | C | ● | ● |
|  | TEEAAESQTAGR |  |  |  |  |  |
|  | TPAWEQQEEPPAPN |  |  |  |  |  |
|  | VWVLEEEGDEER |  |  |  |  |  |
|  | WTLLEEEAVGWQER |  |  |  |  |  |
| ARF4 | DAVLLLFANK | 39 | ● | A | ● | ● |
|  | HYFQNTQGLIFVVDSNDR | 41 | ● | A | ▲ |  |
|  | IQEVADELQK | 27 | ▲ | B | ● |  |
|  | LGLQSLR |  |  |  |  |  |
| BAIAP2 | AFPAQTASGFK | 22.19 | ▲ | B | ● |  |
|  | AIFSHAAGDNSTLLSFK |  |  |  |  |  |
|  | ALAGVTYAAK | 42.07 | ● | A | ● | ● |
|  | DDLAIPPPDYGAASR |  |  |  |  |  |
|  | DGWHYGESEK |  |  |  |  |  |
|  | EGDLITLLVPEAR | 30.45 | ● | A | ● |  |
|  | ELQYIDAISNK | 4.89 | ▲ | B | ● |  |
|  | GWFPFSYTR |  |  |  |  |  |
|  | GYFDALVK | 10.28 | ▲ | B | ● |  |
|  | LSDSYSNTLPVR |  |  |  |  |  |
|  | LTENVYK | 4.06 | ▲ | B | ● |  |
|  | NSAAYHSK |  |  |  |  |  |
|  | NSYATTENK |  |  |  |  |  |
|  | QGELENYVSDGYK | 22 | ▲ | B | ● |  |
|  | SFHNELLTQLEQK |  |  |  |  |  |
|  | SLSPPQSQSK | 16.62 | ▲ | B | ● |  |
|  | SSSTGNLLDK |  |  |  |  |  |
|  | TALTEER |  |  |  |  |  |
|  | VLDSDGSDR |  |  |  |  |  |
|  | YLSAALK |  |  |  |  |  |
| BCCIP | AVESGVPQPPDPPVQR |  |  |  |  |  |
|  | ELAGAHR |  |  |  |  |  |
|  | EVENEDEDDDDSDK |  |  |  |  |  |
|  | FLNDTTKPVGLLLSER |  |  |  |  |  |
|  | LLQQLFLK | 8.21 | ▲ | B | ● |  |
|  | TFVEAGK |  |  |  | ● | ● |
| BROX | ADHTLSSLEPAYSAK |  |  |  |  |  |
|  | ENGFIYFQK |  |  |  |  |  |
|  | ENITEDEAK |  |  |  |  |  |
|  | EPDIKPQK |  |  |  |  |  |
|  | GPGPTVKPSGHLFFR |  |  |  |  |  |
|  | IAAGIFK |  |  |  |  |  |
|  | IPTEAPQLELK |  |  | C | ● | ● |
|  | LITPAEK |  |  |  |  |  |
|  | SLQEAEK |  |  |  |  |  |
| C21orf33 | AALHLSVPRPAAR |  |  |  |  |  |
|  | EVVEAHVDQK | 22.84 | ▲ | B | ● | ● |
|  | GQPSEGESR |  |  |  |  |  |
|  | GVEVTVGHEQEEGGK |  |  |  |  |  |
|  | LAAASAFTSLSPGGR |  |  |  |  |  |
|  | NLSTFAVDGK |  |  |  |  |  |
|  | NVLTESAR |  |  |  |  |  |
|  | VLELTGK |  |  |  |  |  |
|  | WPYAGTAEAIK |  |  |  |  |  |
| CA2 | AVQQPDGLAVLGIFLK | 10.61 | ▲ | B | ● |  |
|  | EPISVSSEQVLK |  |  |  |  |  |
|  | GGPLDGTYR | 10.7 | ▲ | B | ● |  |
|  | HNGPEHWHK |  |  |  |  |  |
|  | QSPVDIDTHTAK |  |  |  |  |  |
|  | SADFTNFDPR | 6.63 | ▲ | B | ● | ● |
|  | VGSAKPGLQK | 1.47 | ▲ | B | ● |  |
|  | VVDVLDSIK | 32.64 | ● | A | ● |  |
|  | YAAELHLVHWNTK |  |  |  |  |  |
| CALM3 | DGDGTITTK | 71 | ● | A | ● |  |
|  | DGNGYISAAELR | 51 | ● | A | ● |  |
|  | DTDSEEEIR | 52.24 | ● | A | ● | ● |
|  | EAFSLFDK | 31.39 | ● | A | ▲ |  |
| CLPP | ALPLIPIVVEQTGR |  |  |  |  |  |
|  | AYDIYSR |  |  |  |  |  |
|  | EPVEAAPAAEPVPAST |  |  |  |  |  |
|  | HSLPNSR |  |  |  |  |  |
|  | LAAHFPAQRPPQR |  |  |  |  |  |
|  | QLYNIYAK |  |  |  |  |  |
|  | TLQNGLALQR |  |  |  |  |  |
|  | YPALGPR |  |  |  |  |  |
| CLTA | ELEEWYAR |  |  |  |  |  |
|  | LEALDANSR |  |  |  |  |  |
|  | LQSEPESIR | 22.18 | ▲ | B | ● | ● |
|  | QDEQLQK |  |  |  |  |  |
|  | SVLISLK |  |  |  |  |  |
|  | VADEAFYK |  |  |  |  |  |
| CNPY3 | ASPLTHSPPDEL |  |  |  |  |  |
|  | EVIGTGYGILDQK |  |  |  |  |  |
|  | GDTAALGGK |  |  |  |  |  |
|  | LLDYSLHK |  |  |  |  |  |
|  | SAFEETGK |  |  |  |  |  |
|  | YVAVELK |  |  |  |  |  |
| COL12A1 | AFLEVLVK |  |  |  |  |  |
|  | ALALGALQNIR |  |  |  |  |  |
|  | DELLAAIK | 19 | ▲ | B | ● | ● |
|  | DLSFSEVTSYGFK |  |  |  |  |  |
|  | DPHTEFTLK |  |  |  |  |  |
|  | DTEPTRPK |  |  |  |  |  |
|  | EVTTPPNQR | 8 | ▲ | B | ● |  |
|  | GDAGEPGLPGR |  |  |  |  |  |
|  | GDTTNTVLQGLK |  |  |  |  |  |
|  | GGSTNTGK |  |  |  |  |  |
|  | GISGAIGPPGPR |  |  |  |  |  |
|  | GQEITVR |  |  |  |  |  |
|  | GSPQDLVTK |  |  |  |  |  |
|  | GTGSSGPR |  |  |  |  |  |
|  | IEQELAAIK |  |  |  |  |  |
|  | IGVLITDGK |  |  |  |  |  |
|  | IIYRPVAGGESR |  |  |  |  |  |
|  | ITEVTSEGFR |  |  |  |  |  |
|  | ITVDPTTDGPTK |  |  |  |  |  |
|  | ITWTQAPGR |  |  |  |  |  |
|  | IVEVFDIGPK |  |  |  |  |  |
|  | LNTYNDK |  |  |  |  |  |
|  | LNWNPSPSPVTGYK |  |  |  |  |  |
|  | LSPADGTR | 8 | ▲ | B | ● |  |
|  | LSWSGAPGK |  |  |  |  |  |
|  | NADEVELK |  |  |  |  |  |
|  | NSDVEIFAVGVK |  |  |  |  |  |
|  | NTFTESAGAR |  |  |  |  |  |
|  | NVGVEVFSLGIK |  |  |  |  |  |
|  | QEFYVSR |  |  |  |  |  |
|  | QGSGTTASR |  |  |  |  |  |
|  | QHALSVGPQTTTLSVR |  |  |  |  |  |
|  | QNSVVLQK |  |  |  |  |  |
|  | SETSTSLK | 16 | ▲ | B | ● |  |
|  | SFEISPNR |  |  |  |  |  |
|  | SLYDDVDTGEK |  |  |  |  |  |
|  | SQDDVEAPSK |  |  |  |  |  |
|  | SQDEVEIPAR |  |  |  |  |  |
|  | TEFNLNQYYQR |  |  |  |  |  |
|  | TEWQLNAHR |  |  |  |  |  |
|  | TGPPGSTGSR |  |  |  |  |  |
|  | TKPLNTVR |  |  |  |  |  |
|  | TLENLIPDTK | 18 | ▲ | B | ● |  |
|  | TLFYGSFHK |  |  |  |  |  |
|  | TLSFFNK | 9 | ▲ | B | ● |  |
|  | TPEEDVK |  |  |  |  |  |
|  | VAIIITDGK |  |  |  |  |  |
|  | VEDIIEAINTFPYR |  |  |  |  |  |
|  | VEYYPVSGGK |  |  |  |  |  |
|  | VGVVQYSSDTR |  |  |  |  |  |
|  | VLVVVTDGR |  |  |  |  |  |
|  | VPPTVTSTVLK |  |  |  |  |  |
|  | VQISLVQYSR |  |  |  |  |  |
|  | VTDETTDSFK |  |  |  |  |  |
|  | VTFHPTGDDR |  |  |  |  |  |
|  | VTWKPAPGK |  |  |  |  |  |
|  | VVYRPHGR |  |  |  |  |  |
|  | WDHAEGNPR |  |  |  |  |  |
|  | WDPASGR |  |  |  |  |  |
| COL4A2 | AGFPGLPGSPGAR |  |  |  |  |  |
|  | AVAGPALR |  |  |  |  |  |
|  | DGLDGFPGLPGPPGDGIK |  |  |  |  |  |
|  | EGFPGPPGFIGPR |  |  |  |  |  |
|  | EGLPGDR |  |  |  |  |  |
|  | GAPGIFGLK |  |  |  |  |  |
|  | GAPGVTGPK |  |  |  |  |  |
|  | GDAGFTGEQGHPGSPGFK |  |  |  |  |  |
|  | GDAGVPGQPGLK |  |  |  |  |  |
|  | GDDGSPGR |  |  |  |  |  |
|  | GDDGWPGAPGLPGFPGLR |  |  |  |  |  |
|  | GDPGEANTLPGPVGVPGQK |  |  |  |  |  |
|  | GDPGQHGLPGFPGLK |  |  |  |  |  |
|  | GDQGAPGER |  |  |  |  |  |
|  | GDTGNPGAPGTPGTK |  |  |  |  |  |
|  | GEAGFFGIPGLK |  |  |  |  |  |
|  | GEPYALPK |  |  |  |  |  |
|  | GFAGINGEPGR |  |  |  |  |  |
|  | GGVSAVPGFR | 8 | ▲ | B | ● | ● |
|  | GIPGFAGADGGPGPR |  |  |  |  |  |
|  | GLAGEPGFK |  |  |  |  |  |
|  | GLDGYQGPDGPR |  |  |  |  |  |
|  | GLGFYGVK |  |  |  |  |  |
|  | GLHGLPGTK |  |  |  |  |  |
|  | GLPGDAGR |  |  |  |  |  |
|  | GLPGEVLGAQPGPR |  |  |  |  |  |
|  | GLPGLPGPK |  |  |  |  |  |
|  | GLPGLPGPPGPTGAK |  |  |  |  |  |
|  | GPPGDPGYPGIPGTK |  |  |  |  |  |
|  | GPPGPPGSAALPGSK |  |  |  |  |  |
|  | GQTGFPGLTGPPGSQGELGR |  |  |  |  |  |
|  | GRPGFPGSK |  |  |  |  |  |
|  | GSEGEPGIR |  |  |  |  |  |
|  | GTTGIPGLK |  |  |  |  |  |
|  | GVPGNIGAPGPK |  |  |  |  |  |
|  | GYPGLSGEK | 14 | ▲ | B | ● |  |
|  | IAVQPGTVGPQGR |  |  |  |  |  |
|  | IGLPGGK |  |  |  |  |  |
|  | LWSGYSLLYFEGQEK |  |  |  |  |  |
|  | SVSIGYLLVK |  |  |  |  |  |
| COMT | EVVDGLEK |  |  |  |  |  |
|  | GPGSEAGP |  |  |  |  |  |
|  | LLSPGAR |  |  |  |  |  |
|  | VTLVVGASQDIIPQLK |  |  | C | ● | ● |
| CRISP1 | LVTDLPNVQEEIVNIHNALR |  |  |  |  |  |
| CYP1A1 | ASRPQVPK |  |  |  |  |  |
|  | FLTPDGAIDK |  |  |  |  |  |
|  | HSSFVPFTIPHSTTR |  |  |  |  |  |
|  | IGSTPVVVLSGLDTIR |  |  | C | ● | ● |
|  | IQEELDTVIGR |  |  | C |  |  |
|  | LAQNGLK |  |  |  |  |  |
|  | LWVNPSEFLPER |  |  | C |  |  |
|  | NPHLALSR |  |  |  |  |  |
|  | QLDENANVQLSDEK |  |  |  |  |  |
|  | VEFSVPLGVK |  |  |  |  |  |
|  | WEVFLFLAILLQR |  |  |  |  |  |
|  | YLPNPSLNAFK |  |  | C |  |  |
| DCTPP1 | DWEQFHQPR |  |  |  |  |  |
|  | FSFSPEPTLEDIR |  |  |  |  |  |
|  | GDTGGEDTAAPGR |  |  |  |  |  |
|  | LHAEFAAER |  |  |  |  |  |
|  | TDGEPGPQGWSPR |  |  |  |  |  |
|  | VDLPLAVLSK | 11 | ▲ | B | ● | ● |
|  | YPAHLAR |  |  |  |  |  |
| DDT | ELALGQDR |  |  |  |  |  |
|  | FFPLESWQIGK |  |  | C | ● | ● |
|  | SHSAHFFEFLTK |  |  |  |  |  |
|  | VPAGLEK |  |  |  |  |  |
| DDX19B | AAQSLLNK |  |  |  |  |  |
|  | DGNPDNETYLHR |  |  |  |  |  |
|  | DPNSPLYSVK | 14.1 | ▲ | B | ▲ |  |
|  | EEETLDTIK |  |  |  |  |  |
|  | IKPDTNGAVVK |  |  |  |  |  |
|  | IQEHFNK |  |  |  |  |  |
|  | LDTDDLDEIEK |  |  |  |  |  |
|  | SNLVDNTNQVEVLQR | 58.4 | ● | A | ▲ | ● |
|  | TASWLAAELSK |  |  |  |  |  |
|  | TNANAEK |  |  |  |  |  |
|  | VVPDPNVIK | 20.05 | ▲ | B | ▲ |  |
| DNAJB11 | EQLTEEAR |  |  |  |  |  |
|  | FQDLGAAYEVLSDSEK |  |  |  |  |  |
|  | GEGLPNFDNNNIK |  |  |  |  |  |
|  | GSLIITFDVDFPK |  |  |  |  |  |
|  | ITRPGAK |  |  |  |  |  |
|  | LALQLHPDR |  |  |  |  |  |
|  | NPDDPQAQEK |  |  |  |  |  |
|  | QYDTYGEEGLK |  |  |  |  |  |
|  | TLEVEIEPGVR | 5.7 | ▲ | B | ● | ● |
|  | TTQLGPGR |  |  |  |  |  |
|  | VYNGLQGY |  |  |  |  |  |
| DNM1L | AEELLAEEK | 18.52 | ▲ | B | ● |  |
|  | ALQGASQIIAEIR |  |  |  |  |  |
|  | DEYAFLQK |  |  |  |  |  |
|  | DIELQIR |  |  |  |  |  |
|  | DTLQSELVGQLYK |  |  |  |  |  |
|  | ELPSAVSR |  |  |  |  |  |
|  | EVDPDGR |  |  |  |  |  |
|  | GHAVNLLDVPVPVAR |  |  |  |  |  |
|  | GTGIVTR |  |  |  |  |  |
|  | GVSPEPIHLK |  |  |  |  |  |
|  | LEEPSLR |  |  |  |  |  |
|  | LGIIGVVNR |  |  |  |  |  |
|  | LYTDFDEIR |  |  |  |  |  |
|  | NIQDSVPK |  |  |  |  |  |
|  | QEIENETER |  |  |  |  |  |
|  | RPLILQLVHVSQEDK |  |  |  |  |  |
|  | SATLLQLITK | 27.87 | ▲ | B | ● | ● |
|  | SQLDINNK |  |  |  |  |  |
|  | SSVLESLVGR | 8.33 | ▲ | B | ● |  |
|  | SVTDSIR |  |  |  |  |  |
|  | SYFLIVR |  |  |  |  |  |
|  | TLAVITK |  |  |  |  |  |
|  | TTGEENGVEAEEWGK |  |  |  |  |  |
|  | VPVGDQPK | 5.08 | ▲ | B | ● |  |
|  | YPSLANR |  |  |  |  |  |
| DPP7 | APDPGFQER |  |  |  |  |  |
|  | ASHPEDPASVVEAR |  |  |  |  |  |
|  | DLFLQGAYDTVR | 16.5 | ▲ | B | ● |  |
|  | DVTADFEGQSPK | 17.15 | ▲ | B | ● |  |
|  | EQQPALR |  |  |  |  |  |
|  | GALLVFAEHR |  |  |  |  |  |
|  | GLQAGAR |  |  |  |  |  |
|  | LDHFNFER |  |  |  |  |  |
|  | LEATIIGEWVK |  |  |  |  |  |
|  | LLSEAQR |  |  |  |  |  |
|  | SLPFGAQSTQR | 16.74 | ▲ | B | ● | ● |
| DPY30 | ERPPNPIEFLASYLLK |  |  |  |  |  |
|  | VDLQSLPTR | 15 | ▲ | B | ● | ● |
| DYNLRB2 | DIDPQNDLTFLR |  |  | C | ▲ | ● |
| EBP | AAVVPLGTWR | 18 | ▲ | B | ● | ● |
|  | HLTHAQSTLDAK |  |  |  |  |  |
| EGFR | DSLSINATNIK | 5.94 | ▲ | B | ● |  |
|  | EISDGDVIISGNK | 46.14 | ● | A | ● |  |
|  | EITGFLLIQAWPENR |  |  |  |  |  |
|  | ELIIEFSK |  |  |  |  |  |
|  | EYHAEGGK |  |  |  |  |  |
|  | GLWIPEGEK |  |  |  |  |  |
|  | IPLENLQIIR |  |  |  |  |  |
|  | ITDFGLAK |  |  |  |  |  |
|  | LFGTSGQK |  |  |  |  |  |
|  | LLGAEEK |  |  |  |  |  |
|  | NLQEILHGAVR | 35.31 | ● | A | ● |  |
|  | NYDLSFLK | 38.95 | ● | A | ● | ● |
|  | TDLHAFENLEIIR |  |  |  |  |  |
|  | VLGSGAFGTVYK | 2.21 | ▲ | B | ▲ |  |
|  | YLVIQGDER | 36.92 | ● | A | ● |  |
| EHHADH | ALQYAFFAER |  |  |  |  |  |
|  | AVIDHTIK |  |  |  |  |  |
|  | AVQAAVQYPYEVGIK |  |  |  |  |  |
|  | EEELFLYLLQSGQAR |  |  |  |  |  |
|  | EWQSLAGSPSSK |  |  |  |  |  |
|  | FSAGADIR |  |  |  |  |  |
|  | GIVISFAR |  |  |  |  |  |
|  | GQGLTGPTLLPGTPAR |  |  |  |  |  |
|  | GTQLLPR |  |  |  |  |  |
|  | GWYQYDKPLGR |  |  |  |  |  |
|  | IHKPDPWLSK |  |  |  |  |  |
|  | ILADEALK |  |  |  |  |  |
|  | IPVIAVDSDK |  |  |  |  |  |
|  | LASQGNPPLK |  |  |  |  |  |
|  | LHNALALIR |  |  |  |  |  |
|  | LTGVPAALDLITSGR |  |  |  |  |  |
|  | NPPVNAISTTLLR |  |  |  |  |  |
|  | NQLATANK |  |  |  |  |  |
|  | QNPDIPQLEPSDYLK |  |  |  |  |  |
|  | TFGLTLGHVVDEIQR |  |  |  |  |  |
|  | THHIEPR |  |  |  |  |  |
|  | TISQDEILER |  |  |  |  |  |
|  | VSDLAGLDVGWK |  |  |  |  |  |
|  | VSDQPLESR |  |  |  |  |  |
|  | VVNSDPVEEAIR | 1 | ▲ | B | ● |  |
|  | WSTPSGASWK |  |  |  | ● | ● |
| ELMOD2 | ATHVVQSEVDK |  |  | C | ● | ● |
|  | IENSLTYSK |  |  |  |  |  |
|  | IFDTYVGAQR |  |  |  |  |  |
|  | QLYLDVESVR |  |  |  |  |  |
|  | QWAEIGFQGDDPK |  |  |  |  |  |
| ENO1 | AAVPSGASTGIYEALELR | 6.5 | ▲ | B | ▲ |  |
|  | AVEHINK | 10.57 | ▲ | B | ● |  |
|  | EGLELLK | 54.65 | ● | A | ● | ● |
|  | GNPTVEVDLFTSK | 41.19 | ● | A | ● |  |
|  | IEEELGSK |  |  |  |  |  |
|  | IGAEVYHNLK | 51.05 | ● | A | ● |  |
|  | LNVTEQEK | 26.41 | ▲ | B | ● |  |
|  | SPDDPSR |  |  |  |  |  |
|  | TIAPALVSK | 33.07 | ● | A | ● |  |
|  | YISPDQLADLYK | 46.06 | ● | A | ● |  |
| ETV6 | ALLLLTK |  |  |  |  |  |
|  | DDVAQWLK |  |  |  |  |  |
|  | EGKPINLSHR |  |  |  |  |  |
|  | IVDPNGLAR |  |  |  |  |  |
|  | LLWDYVYQLLSDSR |  |  |  |  |  |
|  | LQPIYWSR |  |  |  |  |  |
|  | LSEDGLHR |  |  |  |  |  |
| FASN | AAEQYTPK |  |  |  |  |  |
|  | AFEVSENGNLVVSGK |  |  |  |  |  |
|  | AGLYGLPR |  |  |  |  |  |
|  | AQVADVVVSR | 24.95 | ▲ | B | ● |  |
|  | DGAWGAFR |  |  |  |  |  |
|  | DGVVRPLK |  |  |  |  |  |
|  | DNLEFFLAGIGR | 10.81 | ▲ | B | ● |  |
|  | DPSQQELPR | 5.36 | ▲ | B | ● |  |
|  | ELNLVLSVR | 11.94 | ▲ | B | ● |  |
|  | FDASFFGVHPK |  |  |  |  |  |
|  | GDLSSIR |  |  |  |  |  |
|  | GTPLISPLIK |  |  |  |  |  |
|  | GYAVLGGER |  |  |  |  |  |
|  | HGLYLPTR |  |  |  |  |  |
|  | LLEQGLR |  |  |  |  |  |
|  | LQELSSK |  |  |  |  |  |
|  | LQVVDQPLPVR |  |  |  |  |  |
|  | LSFFFDFR |  |  |  |  |  |
|  | LSPDAIPGK | 16.49 | ▲ | B | ● |  |
|  | LYTLQDK |  |  |  |  |  |
|  | QELSFAAR |  |  |  |  |  |
|  | QEPLLIGSTK |  |  |  |  |  |
|  | QVQPEGPYR |  |  |  |  |  |
|  | SDEAVKPFGLK |  |  |  |  |  |
|  | SEGVVAVLLTK | 24.15 | ▲ | B | ● |  |
|  | SHQGLDR |  |  |  |  |  |
|  | TGTVSLEVR |  |  |  |  |  |
|  | TPEAVQK |  |  |  |  |  |
|  | VAAAVDLIIK |  |  |  |  |  |
|  | VFTTVGSAEK | 26.21 | ▲ | B | ● | ● |
|  | VGDPQELNGITR |  |  |  |  |  |
|  | VLEALLPLK | 13.37 | ▲ | B | ● |  |
|  | VLFPATGYLSIVWK |  |  |  |  |  |
|  | VYATILNAGTNTDGFK |  |  |  |  |  |
|  | VYQWDDPDPR |  |  |  |  |  |
|  | YSGTLNLDR |  |  |  |  |  |
| FBLN1_Isoform B | DSFDIIK |  |  |  |  |  |
|  | EFTRPEEIIFLR |  |  | C | ● | ● |
|  | NVVNVHIFVSEYWF |  |  |  |  |  |
|  | QGLEDTHLDASLPTFR |  |  |  |  |  |
|  | QNTPAGSSK |  |  |  |  |  |
|  | QVRPIVGPFHAVLK |  |  |  |  |  |
|  | SQETGDLDVGGLQETDK |  |  |  |  |  |
| FBLN1_Isoform C | DLLLTVK |  |  |  |  |  |
|  | GYHLNEEGTR |  |  | C | ● | ● |
|  | HGTVSSFVAK |  |  |  |  |  |
|  | LFIFVSAEL |  |  |  |  |  |
|  | TGYYFDGISR |  |  | C |  |  |
|  | VSPHSGVVALTKPVPEPR |  |  |  |  |  |
| FKBP1A | AVEPPPGR |  |  |  |  |  |
| GAA | AGYIIPLQGPGLTTTESR | 17.91 | ▲ | B | ● |  |
|  | DAQAHPGRPR |  |  |  |  |  |
|  | GAYTQVIFLAR |  |  |  |  |  |
|  | GELFWDDGESLEVLER |  |  |  |  |  |
|  | GTRPFVISR |  |  |  |  |  |
|  | GVFITNETGQPLIGK |  |  |  |  |  |
|  | NNTIVNELVR |  |  |  |  |  |
|  | STFAGHGR |  |  |  |  |  |
|  | TTPTFFPK | 19.07 | ▲ | B | ● |  |
|  | VTSEGAGLQLQK |  |  |  |  |  |
|  | WGYSSTAITR | 18.66 | ▲ | B | ● | ● |
|  | YEVPLETPR |  |  |  |  |  |
| GARS | AALLLLLPPR |  |  |  |  |  |
|  | APQVDVDK | 10.07 | ▲ | B | ● |  |
|  | AQVSGQSAR |  |  |  |  |  |
|  | ELALQPK | 27.17 | ▲ | B | ● |  |
|  | ELSEALTR | 11.08 | ▲ | B | ● |  |
|  | FQNVADLHLYLYSAK |  |  |  |  |  |
|  | GEFTIETEGK |  |  |  |  |  |
|  | IYLYLTK | 1.8 | ▲ | B | ● |  |
|  | LLARPSLLLR |  |  |  |  |  |
|  | LLEFNQGK | 31 | ● | A | ● |  |
|  | LPFAAAQIGNSFR |  |  |  |  |  |
|  | NNIIQTWR |  |  |  |  |  |
|  | QQGDLVR |  |  |  |  |  |
|  | TFFSFPAVVAPFK | 6.5 | ▲ | B | ● |  |
|  | TPHTATLR |  |  |  |  |  |
|  | TSGHVDK | 3.55 | ▲ | B | ● |  |
|  | TVNVVQFEPSK | 37.3 | ● | A | ● | ● |
|  | VDDSSGSIGR | 12.72 | ▲ | B | ● |  |
|  | VGISPDK |  |  |  |  |  |
|  | VPLVAEKPLK | 6.46 | ▲ | B | ● |  |
|  | YPLFEGQETGK |  |  |  |  |  |
| GART | AAVAGLDK |  |  |  |  |  |
|  | AFAHITGGGLLENIPR | 5 | ▲ | B | ● |  |
|  | AIAFLQQPR |  |  |  |  |  |
|  | ASGLAAGK |  |  |  |  |  |
|  | DPLLASGTDGVGTK |  |  |  |  |  |
|  | EEAWVIGSVVAR |  |  |  |  |  |
|  | EHTLAWK |  |  |  |  |  |
|  | ENLISALEEAK |  |  |  |  |  |
|  | EPNSSAQIDIVISNK |  |  |  |  |  |
|  | EQTEQILR |  |  |  |  |  |
|  | FEGAIYR |  |  |  |  |  |
|  | GDTVATLSER | 27 | ▲ | B | ● | ● |
|  | GYPGDYTK |  |  |  |  |  |
|  | HGIPTAQWK |  |  |  |  |  |
|  | ILSGPFVQK |  |  |  |  |  |
|  | IYSHSLLPVLR |  |  |  |  |  |
|  | LAQSHHVK |  |  |  |  |  |
|  | LDLSVTEAVVAGIAK |  |  |  |  |  |
|  | LGVDLDAQTWR |  |  |  |  |  |
|  | NGSLTNHFSFEK |  |  |  |  |  |
|  | VDLGGFAGLFDLK |  |  |  |  |  |
|  | VLAVTAIR |  |  |  |  |  |
|  | VLIIGSGGR |  |  |  |  |  |
|  | VVTHGGR |  |  |  |  |  |
| GGH | DYEILFK |  |  |  |  |  |
|  | FFNVLTTNTDGK | 26.29 | ▲ | B | ● | ● |
|  | NLDGISHAPNAVK |  |  |  |  |  |
|  | SESEEEK |  |  |  |  |  |
|  | SINGILFPGGSVDLR |  |  |  |  |  |
|  | TAFYLAEFFVNEAR |  |  |  |  |  |
|  | YLESAGAR |  |  |  |  |  |
|  | YPVYGVQWHPEK |  |  |  |  |  |
|  | YYIAASYVK |  |  |  |  |  |
| GLO1 | FEELGVK |  |  |  | ● | ● |
|  | FSLYFLAYEDK | 11.89 | ▲ | B | ● |  |
|  | IAWALSR |  |  |  |  |  |
|  | SLDFYTR |  |  |  |  |  |
| HEXA | AGAVAER |  |  |  |  |  |
|  | ALLSAPWYLNR |  |  |  |  |  |
|  | ELELVTK |  |  |  |  |  |
|  | EVIEYAR |  |  |  |  |  |
|  | GFGEDFK |  |  |  |  |  |
|  | GLETFSQLVWK |  |  |  |  |  |
|  | GLLLDTSR |  |  |  |  |  |
|  | GSYNPVTHIYTAQDVK |  |  |  |  |  |
|  | GYVVWQEVFDNK |  |  |  |  |  |
|  | IQPDTIIQVWR |  |  | C | ● | ● |
|  | ISYGPDWK |  |  |  |  |  |
|  | LTSDLTFAYER |  |  |  |  |  |
|  | LWFSLLLAAAFAGR |  |  |  |  |  |
|  | SAEGTFFINK |  |  |  |  |  |
|  | TEIEDFPR |  |  |  |  |  |
|  | YVLYPNNFR |  |  |  |  |  |
| HLA-A | AHSQIDR |  |  |  |  |  |
|  | APWIEQEGPEYWDR |  |  |  |  |  |
|  | DYIALNEDLR | 24.59 | ▲ | B | ▲ |  |
|  | FDSDAASQR | 13.81 | ▲ | B | ▲ |  |
|  | FIAVGYVDDTQFVR | 47.35 | ● | A | ▲ | ● |
|  | GYQQDAYDGK |  |  |  |  |  |
|  | VDLGTLR | 15.7 | ▲ | B | ▲ |  |
|  | WASVVVPSGQEQR |  |  |  |  |  |
|  | YFTTSVSRPGR |  |  |  |  |  |
| HPRT1 | FFADLLDYIK | 41.99 | ● | A | ● |  |
|  | NVLIVEDIIDTGK | 5.5 | ▲ | B | ● |  |
|  | SVGYKPDFVGFEIPDK |  |  |  |  |  |
|  | VASLLVK |  |  |  |  |  |
|  | VIGGDDLSTLTGK | 53.68 | ● | A | ● | ● |
| HSP90AB1 | ADHGEPIGR |  |  |  |  |  |
|  | ADLINNLGTIAK | 65.87 | ● | A | ▲ |  |
|  | ALLFIPR | 42.71 | ● | A | ● | ● |
|  | APFDLFENK | 4.75 | ▲ | B | ▲ |  |
|  | DDEEKPK |  |  |  |  |  |
|  | EDQTEYLEER | 66.59 | ● | A | ▲ |  |
|  | EGLELPEDEEEK | 28.61 | ● | A | ▲ |  |
|  | EISDDEAEEEK | 0.81 | ▲ | B | ▲ |  |
|  | ELISNASDALDK | 48.32 | ● | A | ▲ |  |
|  | EQVANSAFVER | 52.21 | ● | A | ▲ |  |
|  | FYEAFSK | 18.33 | ▲ | B | ● |  |
|  | GVVDSEDLPLNISR | 39.53 | ● | A | ▲ |  |
|  | HFSVEGQLEFR | 24.81 | ▲ | B | ▲ |  |
|  | HLEINPDHPIVETLR | 15.51 | ▲ | B | ● |  |
|  | HSQFIGYPITLYLEK |  |  |  |  |  |
|  | IDIIPNPQER |  |  |  |  |  |
|  | IEDVGSDEEDDSGK |  |  |  |  |  |
|  | LGIHEDSTNR |  |  |  |  |  |
|  | NPDDITQEEYGEFYK | 7.17 | ▲ | B | ● |  |
|  | SIYYITGESK | 41.12 | ● | A | ▲ |  |
|  | SLTNDWEDHLAVK | 23.38 | ▲ | B | ▲ |  |
|  | SLVSVTK |  |  |  |  |  |
|  | TKPIWTR | 9.63 | ▲ | B | ▲ |  |
|  | YESLTDPSK | 7.47 | ▲ | B | ▲ |  |
|  | YIDQEELNK |  |  |  |  |  |
| HSPD1 | APGFGDNR | 18.2 | ▲ | B | ● |  |
|  | DGVTVAK |  |  |  |  |  |
|  | GANPVEIR | 16.06 | ▲ | B | ● |  |
|  | GIIDPTK | 22.18 | ▲ | B | ● |  |
|  | GYISPYFINTSK | 35.01 | ● | A | ● |  |
|  | IGIEIIK | 8 | ▲ | B | ● |  |
|  | ISSIQSIVPALEIANAHR | 36.99 | ● | A | ● |  |
|  | LSDGVAVLK | 7.06 | ▲ | B | ● |  |
|  | NAGVEGSLIVEK | 26.17 | ▲ | B | ● |  |
|  | TVIIEQSWGSPK | 70.23 | ● | A | ● |  |
|  | VGEVIVTK | 30.8 | ● | A | ● |  |
|  | VGGTSDVEVNEK | 40.79 | ● | A | ● |  |
|  | VGLQVVAVK | 68.71 | ● | A | ● | ● |
|  | VLAPHLTR | 7.93 | ▲ | B | ● |  |
|  | VTDALNATR | 30.54 | ● | A | ● |  |
| HSPE1 | DGDILGK |  |  |  |  |  |
|  | FLPLFDR | 21.82 | ▲ | B | ● |  |
|  | GGEIQPVSVK | 8.31 | ▲ | B | ● |  |
|  | SAAETVTK | 9.79 | ▲ | B | ● |  |
|  | VLLPEYGGTK | 10.05 | ▲ | B | ● |  |
|  | VLQATVVAVGSGSK | 37.03 | ● | A | ● | ● |
| IGFBP7 | AITQVSK |  |  |  |  |  |
|  | DNLAIQTR |  |  |  | ● | ● |
|  | GHYGVQR |  |  |  |  |  |
|  | HEVTGWVLVSPLSK |  |  |  |  |  |
|  | ITVVDALHEIPVK |  |  | C |  |  |
|  | TELLPGDR |  |  |  |  |  |
| IGHM | ALPAPIEK |  |  | C |  |  |
|  | EEQYNSTYR |  |  |  |  |  |
|  | EPQVYTLPPSR |  |  | C |  |  |
|  | FNWYVDGVEVHNAK |  |  | C |  |  |
|  | GLEWVANIK |  |  | C |  |  |
|  | GPSVFPLAPSSK |  |  | C |  |  |
|  | GTTVTVSSASTK |  |  | C |  |  |
|  | SLSLSPGK |  |  |  |  |  |
|  | TTPPVLDSDGSFFLYSK |  |  | C |  |  |
|  | VVSVLTVLHQDWLNGK |  |  | C |  |  |
|  | YYVDSVK |  |  |  |  |  |
| IMPDH2 | DYPLASK |  |  |  |  |  |
|  | EANEILQR | 15.38 | ▲ | B | ▲ |  |
|  | EDLVVAPAGITLK | 10.43 | ▲ | B | ● |  |
|  | HLSSQNR | 18.24 | ▲ | B | ● |  |
|  | LPIVNEDDELVAIIAR | 7.63 | ▲ | B | ● |  |
|  | LVGIISSR |  |  |  |  |  |
|  | NLIDAGVDALR | 7.98 | ▲ | B | ● |  |
|  | VAQGVSGAVQDK | 74.56 | ● | A | ● | ● |
|  | YEQGFITDPVVLSPK |  |  |  |  |  |
|  | YFSEADK |  |  |  |  |  |
|  | YPNLQVIGGNVVTAAQAK |  |  |  |  |  |
| IRF6 | AWAVETGK |  |  |  |  |  |
|  | DNIVAQLK |  |  |  |  |  |
|  | FPGPEHITNEK |  |  |  |  |  |
|  | GLILEVSGHAIYAIR |  |  |  |  |  |
|  | HSPQQEEENTIFK |  |  |  |  |  |
|  | LILVQVIPVVAR | 14.55 | ▲ | B | ● |  |
|  | LQISTPDIK |  |  |  |  |  |
|  | SFDSGSVR |  |  |  |  |  |
|  | YQEGVDDPDPAK | 16.67 | ▲ | B | ● | ● |
| ISG15 | GGGTEPGGR |  |  |  |  |  |
|  | IGVHAFQQR |  |  | C |  |  |
|  | LAVHPSGVALQDR |  |  | C | ● | ● |
|  | LTQTVAHLK |  |  |  |  |  |
|  | SSTYEVR |  |  |  |  |  |
| KPNA4 | DAQVVQVVLDGLSNILK | 25.86 | ▲ | B | ● |  |
|  | EAAWAISNLTISGR | 7.97 | ▲ | B | ▲ |  |
|  | GDFGTQK |  |  |  | ▲ | ● |
|  | IEQLQNHENEDIYK |  |  |  |  |  |
|  | NEVVVELR |  |  |  |  |  |
|  | NPPIDDLIK | 6.96 | ▲ | B | ▲ |  |
|  | VQTAALR |  |  |  |  |  |
| LAMP2 | GILTVDELLAIR | 54.55 | ● | A | ● | ● |
|  | HHHAGYEQF |  |  |  |  |  |
|  | IAVQFGPGFSWIANFTK |  |  |  |  |  |
|  | IPLNDLFR |  |  |  |  |  |
|  | LNSSTIK | 3.69 | ▲ | B | ● |  |
|  | SHTALLR |  |  |  |  |  |
|  | VQPFNVTQGK |  |  |  |  |  |
|  | YLDFVFAVK | 22.93 | ▲ | B | ● |  |
| LAP3 | AAGIDEQENWHEGK |  |  |  |  |  |
|  | ANKPGDVVR | 11 | ▲ | B | ● |  |
|  | DEVPYLR |  |  |  |  |  |
|  | EDDVPQFTSAGENFDK |  |  |  |  |  |
|  | EFVTHPK |  |  |  |  |  |
|  | ETLNISGPPLK |  |  |  |  |  |
|  | FAEIIEK |  |  |  |  |  |
|  | GITFDSGGISIK | 7 | ▲ | B | ● |  |
|  | GLVLGIYSK |  |  |  |  |  |
|  | GSDEPPVFLEIHYK |  |  |  |  |  |
|  | GSPNANEPPLVFVGK |  |  |  |  |  |
|  | GVLFASGQNLAR |  |  |  |  |  |
|  | LFEASIETGDR |  |  |  |  |  |
|  | LYGSGDQEAWQK |  |  |  |  |  |
|  | TEVHIRPK |  |  |  |  |  |
|  | TIQVDNTDAEGR | 26 | ▲ | B | ● | ● |
|  | TLIEFLLR | 14 | ▲ | B | ● |  |
| LARP1 | AVTPVPTK |  |  |  |  |  |
|  | DFQEETVK |  |  |  |  |  |
|  | DFVEAPPPK |  |  |  |  |  |
|  | DSSQTSR |  |  |  |  |  |
|  | DYEAGQLYGLEK |  |  |  |  |  |
|  | EGTGQQER |  |  |  |  |  |
|  | ENGFTQHVYHK |  |  |  |  |  |
|  | ETESAPGSPR |  |  |  |  |  |
|  | FDGVEGPR |  |  |  |  |  |
|  | FEPEYSQIK |  |  |  |  |  |
|  | FQHPSHELLK |  |  |  |  |  |
|  | FQQVPTDALANK |  |  |  |  |  |
|  | FWSFFLR |  |  |  |  |  |
|  | GEGSDSK |  |  |  |  |  |
|  | GEPGPNDVR |  |  |  |  |  |
|  | GGEPDGSAR |  |  |  |  |  |
|  | KPPPAPEGK |  |  |  |  |  |
|  | LASRPTRPPEPR |  |  |  |  |  |
|  | LFGAPEPSTIAR |  |  |  |  |  |
|  | LQEYLGK |  |  |  |  |  |
|  | NLDIDPK |  |  |  |  |  |
|  | QEVENFK |  |  |  |  |  |
|  | QIEYYFSVDNLER |  |  |  |  |  |
|  | QLALEDAK |  |  |  |  |  |
|  | RPRPSPARPK |  |  |  |  |  |
|  | SDESGEEK |  |  |  |  |  |
|  | SDGAGGAR |  |  |  |  |  |
|  | SLPTTVPESPNYR |  |  |  |  |  |
|  | SVQPQSHKPQPTR |  |  | C | ● | ● |
|  | TEEVSNLK |  |  |  |  |  |
|  | TGNHTSR |  |  |  |  |  |
|  | THFDYQFGYR |  |  |  |  |  |
|  | VQALTTDISLIFAALK |  |  | C |  |  |
|  | VVEIVDEK |  |  |  |  |  |
|  | YYSYGLEK |  |  |  |  |  |
| LOC643576 | ALPFWNEEIVPQIK | 18.89 | ▲ | B | ▲ |  |
|  | HGESAWNLENR | 56.59 | ● | A | ● | ● |
|  | HYGGLTGLNK | 39.57 | ● | A | ▲ |  |
|  | VLIAAHGNSLR | 42.92 | ● | A | ▲ |  |
| LSG1 | ADLLTAEQR |  |  |  |  |  |
|  | APAGGSLGR |  |  |  |  |  |
|  | DGQLTVGLVGYPNVGK |  |  |  |  |  |
|  | DNFLEWR |  |  |  |  |  |
|  | DPVTFQHQHQR |  |  |  |  |  |
|  | ENVILINK |  |  |  |  |  |
|  | ESSTADSEAR |  |  |  |  |  |
|  | FVPAEAR |  |  |  |  |  |
|  | HVLEATYGINIITPR |  |  |  |  |  |
|  | LILTPFER | 4 | ▲ | B | ● | ● |
|  | QELLELFK |  |  |  |  |  |
|  | QIENIVDK |  |  |  |  |  |
|  | QIHNFSHLVSK |  |  |  |  |  |
|  | RPNWNQNTTPEELK |  |  |  |  |  |
|  | SDIVVQIVDAR |  |  |  |  |  |
|  | TFFHQENVR |  |  |  |  |  |
|  | TGLLSFEESQR |  |  |  |  |  |
|  | VSVSATPGHTK |  |  |  |  |  |
| LYPLA1 | ALIDQEVK |  |  |  |  |  |
|  | ASFPQGPIGGANR | 21.56 | ▲ | B | ● |  |
|  | NGIPSNR |  |  |  |  |  |
|  | QAAENIK |  |  |  |  |  |
|  | TLVNPANVTFK | 40.1 | ● | A | ● | ● |
| MAPK14 | DLLIDEWK |  |  |  |  |  |
|  | HENVIGLLDVFTPAR |  |  |  |  |  |
|  | ILDFGLAR |  |  |  |  |  |
|  | ISSESAR |  |  |  |  |  |
|  | LSRPFQSIIHAK |  |  |  |  |  |
|  | LTDDHVQFLIYQILR |  |  |  |  |  |
|  | TIWEVPER |  |  |  |  |  |
|  | TLFPGTDHIDQLK |  |  |  |  |  |
|  | YIHSADIIHR | 6 | ▲ | B | ● | ● |
| MMP1 | ADVDHAIEK |  |  |  |  |  |
|  | AFQLWSNVTPLTFTK |  |  | C |  |  |
|  | DGFFYFFHGTR |  |  | C |  |  |
|  | DIYSSFGFPR |  |  | C | ● | ● |
|  | HIDAALSEENTGK |  |  |  |  |  |
|  | IENYTPDLPR |  |  |  |  |  |
|  | LTFDAITTIR |  |  | C |  |  |
|  | NSGPVVEK |  |  |  |  |  |
|  | SQNPVQPIGPQTPK |  |  |  |  |  |
|  | TYFFVANK |  |  |  |  |  |
|  | VTGKPDAETLK |  |  |  |  |  |
|  | WEQTHLTYR |  |  |  |  |  |
|  | YWAVQGQNVLHGYPK |  |  |  |  |  |
| MTFP1 | AGEVPSPEAGR |  |  |  |  |  |
|  | LYPTVGKPSSS |  |  |  |  |  |
|  | SVDFLLDSSLR | 8 | ▲ | B | ● | ● |
|  | YLGYANEVGEAFR |  |  |  |  |  |
| NDRG1 | SHTSEGTR |  |  |  |  |  |
|  | TASGSSVTSLDGTR | 16 | ▲ | B | ● | ● |
| NIT2 | AGTEEAIVYSDIDLK |  |  |  |  |  |
|  | AVDNQVYVATASPAR | 62.53 | ● | A | ● | ● |
|  | EAATQGAK |  |  |  |  |  |
|  | FAELAQIYAQR | 1.93 | ▲ | B | ● |  |
|  | IHLFDIDVPGK |  |  |  |  |  |
|  | IPGESTQK |  |  |  |  |  |
|  | ITFQESK |  |  |  |  |  |
|  | LALIQLQISSIK | 2.85 | ▲ | B | ● |  |
|  | QQIPVFR |  |  |  |  |  |
|  | YFPEYAEK |  |  |  |  |  |
| NTPCR | GKPLALVEEIR |  |  | C |  |  |
|  | HVFLTGPPGVGK |  |  |  |  |  |
|  | IGFDVVTLSGTR |  |  | C | ● | ● |
|  | QTLSTPGTIILGTIPVPK |  |  |  |  |  |
|  | SSGVPVDGFYTEEVR |  |  |  |  |  |
|  | VGLEPPPGK |  |  |  |  |  |
| NUDC | DAENHEAQLK |  |  |  |  |  |
|  | ELTDEEAER | 38.32 | ● | A | ● | ● |
|  | GQPAIIDGELYNEVK | 34.6 | ● | A | ● |  |
|  | INPENSK |  |  |  |  |  |
|  | LITQTFSHHNQLAQK |  |  |  |  |  |
|  | LKPNLGNGADLPNYR |  |  |  |  |  |
|  | LQLEIDQK |  |  |  |  |  |
|  | LSDLDSETR |  |  |  |  |  |
|  | LVSSDPEINTK | 18.74 | ▲ | B | ● |  |
|  | NGSLDSPGK |  |  |  |  |  |
|  | QDTEEDEEEDEK |  |  |  |  |  |
|  | SETSGPQIK |  |  |  |  |  |
|  | VEESSWLIEDGK | 47.95 | ● | A | ● |  |
|  | VVTVHLEK | 35.15 | ● | A | ● |  |
| NUDT5 | ELEEETGYK | 44.82 | ● | A | ● |  |
|  | EQTADGVAVIPVLQR | 23.39 | ▲ | B | ● |  |
|  | HANAKPFEVPFLK | 1.42 | ▲ | B | ● |  |
|  | LDALVAEEHLTVDAR | 9.81 | ▲ | B | ● |  |
|  | QYIISEELISEGK | 43.95 | ● | A | ● | ● |
|  | VYSYALALK | 3.59 | ▲ | B | ● |  |
| NUP133 | EPEYVPWTATSGPGGIR |  |  |  |  |  |
|  | EYEIPSNLTPADVFFR |  |  |  |  |  |
|  | FSLPQEK |  |  |  |  |  |
|  | GLPLGSAVSSPVLFSPVGR |  |  |  |  |  |
|  | GPLAGLGPGSTPR |  |  |  |  |  |
|  | HAYSWDINR |  |  |  |  |  |
|  | IALSPITK |  |  |  |  |  |
|  | LGSFPVR |  |  |  |  |  |
|  | LIPESSGK |  |  |  |  |  |
|  | LSAAIVLK |  |  |  |  |  |
|  | LSDLVNTAILIALNK |  |  |  |  |  |
|  | NETIAQEDK |  |  |  |  |  |
|  | NSGLVSITSR |  |  |  |  |  |
|  | QHEIVLK |  |  |  |  |  |
|  | SSFYSLTSSNISK |  |  |  |  |  |
|  | TFGSSLPVK |  |  |  |  |  |
|  | TLLGLSK |  |  |  |  |  |
|  | TPGTGSR |  |  |  |  |  |
|  | VAYPQADSNLR |  |  |  |  |  |
|  | WELDDSSEK |  |  |  |  |  |
| PAPSS1 | ATNVTYQAHHVSR |  |  |  |  |  |
|  | AWTVLTEYYK |  |  |  |  |  |
|  | DIVPVDASYEVK |  |  |  |  |  |
|  | DLYEPSHGAK |  |  |  |  |  |
|  | ELYVPENK |  |  |  |  |  |
|  | GQVVGTR |  |  |  |  |  |
|  | LTPTELK |  |  |  |  |  |
|  | NLGFSPEDR |  |  |  |  |  |
|  | NPEFFEHR |  |  |  |  |  |
|  | RPVLLLHPLGGWTK |  |  |  |  |  |
|  | TDAETLPALK | 11.29 | ▲ | B | ● | ● |
|  | VYWNDGLDQYR |  |  |  |  |  |
| PEX19 | DALFASQEK |  |  |  |  |  |
|  | DVLYPSLK |  |  |  |  |  |
|  | ELAEEEPHLVEQFQK |  |  |  |  |  |
|  | ELEELLESALDDFDK |  |  |  |  |  |
|  | ESLPPEQFEK |  |  |  |  |  |
|  | ETLSGLAK |  |  |  |  |  |
|  | LSEAAGR |  |  |  |  |  |
|  | SPGDTAK |  |  |  |  |  |
|  | YPEWLQSHR |  |  |  |  |  |
| PFDN1 | AFTELQAK | 49.74 | ● | A | ● | ● |
|  | EAEDNIR | 20.52 | ▲ | B | ● |  |
|  | EAIHSQLLEK |  |  |  |  |  |
|  | LADIQIEQLNR | 43.52 | ● | A | ● |  |
|  | VIDTQQK |  |  |  |  |  |
| PFDN4 | AAAEDVNVTFEDQQK | 18 | ▲ | B | ● | ● |
|  | FGSNINLEADES | 8 | ▲ | B | ● |  |
|  | NLQEEIDALESR |  |  |  |  |  |
| PGK1 | AAVPSIK | 12.72 | ▲ | B | ● |  |
|  | ELNYFAK |  |  |  |  |  |
|  | FHVEEEGK |  |  |  |  |  |
|  | ITLPVDFVTADK | 32.1 | ● | A | ● | ● |
|  | LGDVYVNDAFGTAHR | 59.85 | ● | A | ▲ |  |
|  | NNQITNNQR |  |  |  |  |  |
|  | VLPGVDALSNI | 21.57 | ▲ | B | ● |  |
|  | VSHVSTGGGASLELLEGK | 46.41 | ● | A | ▲ |  |
|  | YAEAVTR | 8.15 | ▲ | B | ● |  |
|  | YSLEPVAVELK | 27.53 | ▲ | B | ● |  |
| PITPNB | ADEDPALFQSVK |  |  |  |  |  |
|  | GPLGPNWK | 4.46 | ▲ | B | ● |  |
|  | GTSAADV |  |  |  |  |  |
|  | IFTNFHR |  |  |  |  |  |
|  | NETGGGEGIEVLK | 52.88 | ● | A | ● | ● |
|  | SQVEPADYK | 26.79 | ▲ | B | ● |  |
|  | TVEIVHIDIADR | 26.95 | ▲ | B | ● |  |
|  | VENFIQK |  |  |  |  |  |
|  | WWGLQSK |  |  |  |  |  |
| PLIN2 | AYQQALSR |  |  |  |  |  |
|  | DAVTTTVTGAK |  |  |  |  |  |
|  | DQYPYLK |  |  |  |  |  |
|  | EVSDSLLTSSK |  |  |  |  |  |
|  | GAVTGAK |  |  |  |  |  |
|  | GAVTGSVEK |  |  |  |  |  |
|  | IQDAQDK |  |  |  |  |  |
|  | LGSLSTK |  |  |  | ● | ● |
|  | LPILNQPSTQIVANAK |  |  |  |  |  |
|  | LYLSWVEWK |  |  |  |  |  |
|  | NVYSANQK |  |  |  |  |  |
|  | SSQETQR |  |  |  |  |  |
|  | SVVSGSINTVLGSR |  |  | C |  |  |
|  | VEGFDLVQKPSYYVR |  |  |  |  |  |
| PLS3 | AGKPHLVLGLLWQIIK |  |  |  |  |  |
|  | ANDDIIVNWVNR | 23.21 | ▲ | B | ● |  |
|  | AYFHLLNQIAPK |  |  |  |  |  |
|  | DELDELK |  |  |  |  |  |
|  | IGLFADIELSR |  |  |  |  |  |
|  | INNFSADIK |  |  |  |  |  |
|  | ISFDEFVYIFQEVK |  |  |  |  |  |
|  | LNLAFVANLFNK | 40.38 | ● | A | ● |  |
|  | LSPEELLLR | 40.13 | ● | A | ▲ |  |
|  | NEALAALLR | 48.78 | ● | A | ● | ● |
|  | QFVTPADVVSGNPK | 6.1 | ▲ | B | ▲ |  |
|  | SGNLTEDDK |  |  |  |  |  |
|  | STSIQSFK |  |  |  |  |  |
|  | TLSEAGK |  |  |  |  |  |
|  | VNKPPYPK | 20.09 | ▲ | B | ▲ |  |
|  | VPVDWSK |  |  |  |  |  |
|  | VYALPEDLVEVKPK |  |  |  |  |  |
|  | WANFHLENSGWQK |  |  |  |  |  |
|  | YAFVNWINK |  |  |  |  |  |
|  | YTLNVLEDLGDGQK | 62.99 | ● | A | ● |  |
| PREP | AFVEAQNK |  |  |  |  |  |
|  | AGHGAGKPTAK |  |  |  |  |  |
|  | DETAVQDYHGHK |  |  |  |  |  |
|  | EGYTSPK |  |  |  |  |  |
|  | ELPDVLER |  |  |  |  |  |
|  | FIATLQYIVGR |  |  |  |  |  |
|  | GGGEYGETWHK |  |  |  |  |  |
|  | GGILANK |  |  |  |  |  |
|  | NILQLHDLTTGALLK |  |  | C | ● | ● |
|  | QHFEWLVK |  |  |  |  |  |
|  | QSNPLLIHVDTK |  |  |  |  |  |
|  | SDGTETSTNLHQK |  |  |  |  |  |
|  | TFPLDVGSIVGYSGQK |  |  |  |  |  |
|  | VINIDFR |  |  |  |  |  |
|  | VLVPEHEK |  |  |  |  |  |
|  | VLYVQDSLEGEAR |  |  |  |  |  |
|  | VVPLHSLK |  |  |  |  |  |
|  | YFYFYNTGLQNQR |  |  |  |  |  |
|  | YSPLHNVK |  |  |  |  |  |
|  | YVLLSIR |  |  |  |  |  |
| PRPF3 | AADHLKPFLDDSTLR |  |  |  |  |  |
|  | AAELQAR |  |  |  |  |  |
|  | AHEEANAAR |  |  |  |  |  |
|  | DQTKPTPLILDEQGR |  |  |  |  |  |
|  | DVNVVVVEGGPK |  |  |  |  |  |
|  | EDISQGVHISVYR |  |  |  |  |  |
|  | EIELTHR |  |  |  |  |  |
|  | ELDELKPWIEK |  |  |  |  |  |
|  | EVFGDDSEISK |  |  |  |  |  |
|  | GDDDEESDEEAVK |  |  |  |  |  |
|  | LALIAPK |  |  |  |  |  |
|  | LFEAVEEGR |  |  |  |  |  |
|  | LQAEISQAAR |  |  |  | ● | ● |
|  | NLSNPAK |  |  |  |  |  |
|  | QLSFISPPTPQPK |  |  |  |  |  |
|  | TGIHTSTR |  |  |  |  |  |
|  | TPSSSQPER | 12 | ▲ | B | ● |  |
|  | TVDATGK |  |  |  |  |  |
|  | VLGTEAVQDPTK |  |  |  |  |  |
|  | VSIAPSQR |  |  |  |  |  |
|  | WDEQTSNTK |  |  |  |  |  |
| PSPH | EEGIDELAK |  |  |  |  |  |
|  | LALIQPSR |  |  |  |  |  |
|  | LIAEQPPHLTPGIR |  |  |  |  |  |
|  | LNIPATNVFANR | 7.09 | ▲ | B | ● |  |
|  | NVQVFLISGGFR |  |  |  |  |  |
|  | SIVEHVASK | 28.69 | ▲ | B | ● | ● |
|  | WYITDFVELLGELEE |  |  |  |  |  |
| RAB11B | AITSAYYR | 7.12 | ▲ | B | ▲ |  |
|  | AQIWDTAGQER | 21.2 | ▲ | B | ▲ |  |
|  | AVPTDEAR | 9.89 | ▲ | B | ▲ |  |
|  | DDEYDYLFK | 9.48 | ▲ | B | ▲ |  |
|  | GAVGALLVYDIAK | 40.25 | ● | A | ▲ |  |
|  | HLTYENVER | 22.44 | ▲ | B | ▲ |  |
|  | NEFNLESK | 16.54 | ▲ | B | ▲ |  |
|  | NILTEIYR |  |  |  |  |  |
|  | SIQVDGK |  |  |  |  |  |
|  | STIGVEFATR | 44.5 | ● | A | ▲ | ● |
|  | VVLIGDSGVGK | 42.42 | ● | A | ▲ |  |
| RAB3GAP2 | AAASGNENIQPPPLAYK |  |  |  |  |  |
|  | ADFSPFGNSQGPSR |  |  |  |  |  |
|  | AVFLVPK | 4 | ▲ | B | ● |  |
|  | DAENPDEPK |  |  |  |  |  |
|  | DAQIGWIQTVEDLHER |  |  |  |  |  |
|  | DFLFPHLR |  |  |  |  |  |
|  | DGVLPVK |  |  |  |  |  |
|  | EEILSGALR |  |  |  |  |  |
|  | ELILDIK |  |  |  |  |  |
|  | FGLPDSR |  |  |  |  |  |
|  | GGIADSVAK |  |  |  |  |  |
|  | GILEVWSTQQGPR |  |  |  |  |  |
|  | GNTQTSK |  |  |  |  |  |
|  | HEEEAVQK |  |  |  |  |  |
|  | LLYPGYK |  |  |  |  |  |
|  | LQALLEK |  |  |  |  |  |
|  | LTSALFNAASGWLGWK |  |  |  |  |  |
|  | QALESILASER |  |  |  |  |  |
|  | QDFSPEVLK |  |  |  |  |  |
|  | SPNLDLVETEIK |  |  |  |  |  |
|  | TASNIGGFNAAIK |  |  |  |  |  |
|  | TFLEYLEYEK |  |  |  |  |  |
|  | TVNVPFHLALSDK |  |  |  |  |  |
|  | VAQFLVIYAPR |  |  |  |  |  |
|  | VEPATPLAVR |  |  |  | ● | ● |
|  | VGAFNVGK |  |  |  |  |  |
|  | VILLDVAR |  |  |  |  |  |
|  | VSSLQAEPLPR |  |  |  |  |  |
| RNASET2 | AYWPDVIHSFPNR |  |  |  |  |  |
|  | DPPDYWTIHGLWPDK |  |  |  |  |  |
|  | ELDLNSVLLK |  |  | C | ● | ● |
|  | QEVWLANGAAESR |  |  |  |  |  |
|  | SWPFNLEEIK |  |  |  |  |  |
|  | VYGVIPK |  |  |  |  |  |
| RPL7L1 | HSLAFVVR |  |  |  |  |  |
|  | IDGVSLLVQR |  |  |  |  |  |
|  | IFSGVFVK |  |  |  |  |  |
|  | IPLVPENLLK |  |  |  |  |  |
|  | IVEPYVTWGFPNLK |  |  |  |  |  |
|  | LESFLHDSWR |  |  |  |  |  |
|  | LEVKPHALELPDK |  |  |  |  |  |
|  | TIPLTDNTVIEEHLGK |  |  | C | ● | ● |
|  | VTPQNLK |  |  |  |  |  |
| RUVBL1 | ALESSIAPIVIFASNR | 7.85 | ▲ | B | ● |  |
|  | AVLLAGPPGTGK | 52.31 | ● | A | ● | ● |
|  | EHVEEISELFYDAK |  |  |  |  |  |
|  | GLGLDESGLAK |  |  |  |  |  |
|  | IASHSHVK |  |  |  |  |  |
|  | ILADQQDK |  |  |  |  |  |
|  | LDPSIFESLQK | 6.57 | ▲ | B | ● |  |
|  | QAASGLVGQENAR | 38.85 | ● | A | ● |  |
|  | TALALAIAQELGSK | 43.33 | ● | A | ● |  |
|  | TISHVIIGLK | 1.83 | ▲ | B | ● |  |
|  | VEAGDVIYIEANSGAVK | 2.49 | ▲ | B | ● |  |
|  | YSVQLLTPANLLAK | 12.82 | ▲ | B | ● |  |
| SARNP | EEEPPEK |  |  |  |  |  |
|  | FGISSVPTK |  |  |  |  |  |
|  | FGIVTSSAGTGTTEDTEAK | 28 | ▲ | B | ● |  |
|  | FGLNVSSISR | 16 | ▲ | B | ● |  |
|  | FNVPVSLESK |  |  |  |  |  |
|  | ITSEIPQTER | 28 | ▲ | B | ● | ● |
|  | TVDVAAEK |  |  |  |  |  |
| SERPINA3 | ADLSGITGAR |  |  | C |  |  |
|  | AVLDVFEEGTEASAATAVK |  |  | C |  |  |
|  | EIGELYLPK |  |  |  |  |  |
|  | EQLSLLDR |  |  |  |  |  |
|  | ITLLSALVETR |  |  | C | ● | ● |
|  | LINDYVK |  |  |  |  |  |
|  | LYGSEAFATDFQDSAAAK |  |  |  |  |  |
|  | NLAVSQVVHK |  |  | C |  |  |
|  | YTGNASALFILPDQDK |  |  |  |  |  |
| SERPINB5_Isoform 2 | DVPFGFQTVTSDVNK | 27 | ▲ | B | ● |  |
|  | ELETVDFK | 32 | ● | A | ● | ● |
|  | FSESETK |  |  |  |  |  |
|  | GQINNSIK | 9 | ▲ | B | ● |  |
|  | ILVVNAAYFVGK |  |  |  |  |  |
|  | LSSFYSLK |  |  |  |  |  |
|  | SLNLSTEFISSTK |  |  |  |  |  |
|  | SPIIDVK |  |  |  |  |  |
| SERPINB5_maspin | DELNADHPFIYIIR |  |  |  |  |  |
|  | DIPFGFQTVTSDVNK |  |  |  |  |  |
|  | DVEDESTGLEK |  |  |  |  |  |
|  | FPESETK |  |  |  |  |  |
|  | GVALSNVIHK |  |  |  |  |  |
|  | IIELPFQNK | 14 | ▲ | B | ● |  |
|  | NIIFFGK | 24 | ▲ | B | ● | ● |
| SERPINH1 | AATLAER | 20.63 | ▲ | B | ● |  |
|  | ATTASQAK | 0.03 | ▲ | B | ● |  |
|  | AVAISLPK | 33.78 | ● | A | ● | ● |
|  | AVLSAEQLR |  |  |  |  |  |
|  | DEEVHAGLGELLR | 9.47 | ▲ | B | ● |  |
|  | DTQSGSLLFIGR | 22.23 | ▲ | B | ● |  |
|  | GVVEVTHDLQK | 35.93 | ● | A | ● |  |
|  | HLAGLGLTEAIDK |  |  |  |  |  |
|  | KPAAAAAPGTAEK | 6.75 | ▲ | B | ● |  |
|  | LFYADHPFIFLVR |  |  |  |  |  |
|  | LYGPSSVSFADDFVR | 5.9 | ▲ | B | ● |  |
|  | SLSNSTAR |  |  |  |  |  |
|  | TGLYNYYDDEK | 4.3 | ▲ | B | ● |  |
| SFN | EAGDAESR |  |  |  |  |  |
|  | NVVGGQR |  |  |  |  |  |
|  | SNEEGSEEK |  |  |  |  |  |
|  | VLSSIEQK |  |  |  |  |  |
|  | YLAEVATGDDK | 45 | ● | A | ● | ● |
| SLC20A2 | AADSSAPEDSEK |  |  | C | ● | ● |
|  | EDPAEEEK |  |  |  |  |  |
|  | EDPVPNGLR |  |  |  |  |  |
|  | GPEEKPAQESNYR |  |  |  |  |  |
|  | LASELADPDQPR |  |  |  |  |  |
|  | LVGDTVSYSK |  |  |  |  |  |
|  | SDGHVYHTVHK |  |  |  |  |  |
|  | SPISNGTFGFDGHTR |  |  |  |  |  |
|  | VGSVVAVGWIR |  |  |  |  |  |
|  | VQEAESPVFK |  |  |  |  |  |
|  | VSDESLSK |  |  |  |  |  |
| SLC3A2 | ADLLLSTQPGR | 34.33 | ● | A | ● |  |
|  | DASSFLAEWQNITK |  |  |  |  |  |
|  | EAVELDAVLEVPVPVR |  |  |  |  |  |
|  | EDFDSLLQSAK | 64.83 | ● | A | ● | ● |
|  | EEGSPLELER | 10.23 | ▲ | B | ● |  |
|  | EVELNELEPEK | 50.39 | ● | A | ● |  |
|  | GENSWFSTQVDTVATK | 46.5 | ● | A | ● |  |
|  | GLVLGPIHK |  |  |  |  |  |
|  | GQSEDPGSLLSLFR | 28.66 | ● | A | ● |  |
|  | HWDQNER |  |  |  |  |  |
|  | LDYLSSLK | 36.15 | ● | A | ● |  |
|  | LEPHEGLLLR |  |  |  |  |  |
|  | LLTSFLPAQLLR | 26.56 | ▲ | B | ● |  |
|  | SLVTQYLNATGNR | 14.04 | ▲ | B | ● |  |
|  | TSPSSPAPLPHQEATPR |  |  |  |  |  |
|  | VAEDEAEAAAAAK | 66.01 | ● | A | ● |  |
|  | VAGSPGWVR |  |  |  |  |  |
|  | VILDLTPNYR | 6.45 | ▲ | B | ● |  |
|  | WWHTGALYR |  |  |  |  |  |
| SLC4A2 | ELPPPAPPAGITR |  |  |  |  |  |
|  | EPGPTPR | 13.24 | ▲ | B | ● |  |
|  | FEDVPGVR |  |  |  |  |  |
|  | FEEDVEEETER |  |  |  |  |  |
|  | FEEILQEAGSR |  |  |  |  |  |
|  | GGEEPGR |  |  |  |  |  |
|  | GSTQSGR |  |  |  |  |  |
|  | GWVINPLGEK |  |  |  |  |  |
|  | HSHPSDEK | 16.85 | ▲ | B | ● |  |
|  | LLPTGAGLEPK |  |  |  |  |  |
|  | LSVPSGFSVTAPEK |  |  |  |  |  |
|  | NQEPQWR |  |  |  |  |  |
|  | QFHEAAYLADER |  |  |  |  |  |
|  | SLAGQSGQGKPR |  |  |  |  |  |
|  | SVAHFQR |  |  |  |  |  |
|  | SYGEEDFEYHR |  |  |  |  |  |
|  | SYNLQER |  |  |  |  |  |
|  | TGRPFGGLIR |  |  |  |  |  |
|  | TPQGPGR | 21.11 | ▲ | B | ● | ● |
|  | WGKPHVASLSFR |  |  |  |  |  |
|  | YPHYLSDFR |  |  |  |  |  |
| SMS | ADGETILK | 15 | ▲ | B | ● | ● |
|  | EEIDSILNK |  |  |  |  |  |
|  | ELSQDSTGR |  |  |  |  |  |
|  | NGSFANLR |  |  |  |  |  |
|  | YWPTADGR |  |  |  |  |  |
| SNRPG | AHPPELK |  |  |  |  |  |
|  | HVQGILR |  |  |  |  |  |
| SRC | DAWEIPR |  |  |  |  |  |
|  | EVLDQVER |  |  |  |  |  |
|  | GPSAAFAPAAAEPK | 26.24 | ▲ | B | ● |  |
|  | GSLLDFLK | 16.89 | ▲ | B | ▲ |  |
|  | LDSGGFYITSR |  |  |  |  |  |
|  | LFGGFNSSDTVTSPQR |  |  |  |  |  |
|  | LIEDNEYTAR | 14.26 | ▲ | B | ▲ |  |
|  | LLLNAENPR | 18.87 | ▲ | B | ● |  |
|  | LQIVNNTR |  |  |  |  |  |
|  | SDVWSFGILLTELTTK |  |  |  |  |  |
|  | TETDLSFK | 8.6 | ▲ | B | ● |  |
|  | TQFNSLQQLVAYYSK |  |  |  |  |  |
|  | VADFGLAR |  |  |  |  |  |
|  | WTAPEAALYGR | 39.31 | ● | A | ▲ | ● |
| STK25 | AANVLLSEQGDVK |  |  |  |  |  |
|  | ADIWSLGITAIELAK |  |  | C | ▲ | ● |
|  | GFANQHSR |  |  |  |  |  |
|  | GIDNHTK |  |  |  |  |  |
|  | GLDYLHSER |  |  |  |  |  |
|  | GSFGEVYK |  |  |  |  |  |
|  | GTALHSSQKPAEPVK |  |  |  |  |  |
|  | LADFGVAGQLTDTQIK |  |  |  |  |  |
|  | NHLTSTR |  |  |  |  |  |
|  | NSPPTLEGQHSKPFK |  |  |  |  |  |
|  | QSAYDFK |  |  |  |  |  |
|  | TSFLTELIDR |  |  |  |  |  |
|  | VDPEELFTK |  |  |  |  |  |
|  | VLFLIPK |  |  |  |  |  |
|  | YFGSYLK |  |  |  |  |  |
| STMN1 | AIEENNNFSK | 44 | ● | A | ● |  |
|  | ASGQAFELILSPR | 69 | ● | A | ● |  |
|  | DLSLEEIQK | 60 | ● | A | ▲ |  |
|  | DPADETEAD |  |  |  |  |  |
|  | ESVPEFPLSPPK | 29 | ● | A | ● | ● |
|  | LEAAEER | 42 | ● | A | ▲ |  |
|  | SHEAEVLK | 47 | ● | A | ● |  |
| TAGLN2 | DDGLFSGDPNWFPK | 21.02 | ▲ | B | ● |  |
|  | DVGRPQPGR |  |  |  |  |  |
|  | ENFQNWLK |  |  |  |  |  |
|  | GPAYGLSR | 35.35 | ● | A | ● | ● |
|  | NFSDNQLQEGK |  |  |  |  |  |
| TAX1BP3 | VSEGGPAEIAGLQIGDK | 22.06 | ▲ | B | ● | ● |
| TFG | AQLGEDIR |  |  |  |  |  |
|  | LLSNDEVTIK |  |  | C | ● | ● |
|  | LTLFVNGQPRPLESSQVK |  |  |  |  |  |
|  | NQDEINK |  |  |  |  |  |
|  | NRPPFGQGYTQPGPGYR |  |  |  |  |  |
|  | SASDSSGK |  |  |  |  |  |
| TFRC | AAAEVAGQFVIK |  |  |  |  |  |
|  | AATVTGK | 15 | ▲ | B | ● |  |
|  | AFTYINLDK |  |  |  |  |  |
|  | ANVTKPK | 8 | ▲ | B | ● |  |
|  | AVLGTSNFK | 21 | ▲ | B | ● |  |
|  | DAWGPGAAK |  |  |  |  |  |
|  | DENLALYVENQFR |  |  |  |  |  |
|  | DGFQPSR |  |  |  |  |  |
|  | DSAQNSVIIVDK | 43 | ● | A | ● | ● |
|  | EEPGEDFPAAR |  |  |  |  |  |
|  | GFVEPDHYVVVGAQR |  |  |  |  |  |
|  | ILNIFGVIK |  |  |  |  |  |
|  | LAGTESPVR | 15 | ▲ | B | ● |  |
|  | LAVDEEENADNNTK |  |  |  |  |  |
|  | LDSTDFTGTIK | 2 | ▲ | B | ● |  |
|  | LLNENSYVPR |  |  |  |  |  |
|  | LTHDVELNLDYER |  |  |  |  |  |
|  | LTTDFGNAEK |  |  |  |  |  |
|  | LTVSNVLK | 5 | ▲ | B | ● |  |
|  | LVHANFGTK |  |  |  |  |  |
|  | LVYLVENPGGYVAYSK |  |  |  |  |  |
|  | LYWDDLK |  |  |  |  |  |
|  | QNNGAFNETLFR |  |  |  |  |  |
|  | SAFSNLFGGEPLSYTR | 12 | ▲ | B | ● |  |
|  | SGVGTALLLK |  |  |  |  |  |
|  | SSGLPNIPVQTISR | 7 | ▲ | B | ● |  |
|  | VEYHFLSPYVSPK |  |  |  |  |  |
|  | VSASPLLYTLIEK |  |  |  |  |  |
|  | YNSQLLSFVR |  |  |  |  |  |
| THY1 | HVLFGTVGVPEHTYR |  |  | C | ● | ● |
|  | VLYLSAFTSK |  |  | C |  |  |
| TIMM13 | VQIAVANAQELLQR | 35.59 | ● | A | ● | ● |
| TIMM8A | FIDTSQFILNR |  |  | C | ● | ● |
|  | SKPVFSESLSD |  |  |  |  |  |
| TINAGL1 | ELAPGLHLR |  |  |  |  |  |
|  | GGIYSHTPVSLGRPER |  |  |  |  |  |
|  | HGTHSVK |  |  |  |  |  |
|  | ITGWGEETLPDGR |  |  | C | ● | ● |
|  | LDGAWWFLR |  |  |  |  |  |
|  | YWTAANSWGPAWGER |  |  |  |  |  |
| TMEM43 | DLVNIGLK | 6.77 | ▲ | B | ● |  |
|  | EYTEDGQVK | 43.1 | ● | A | ● | ● |
|  | FFLSSGLIDK | 3.31 | ▲ | B | ● |  |
|  | GDFFYHSENPK |  |  |  |  |  |
|  | GDQLVPFSTK | 7.35 | ▲ | B | ● |  |
|  | ILYTLVDWFPVFR |  |  |  |  |  |
|  | LEDPHVDIIR | 6.74 | ▲ | B | ● |  |
|  | LLSDPNYGVHLPAVK |  |  |  |  |  |
|  | LVHIIGALR | 13.28 | ▲ | B | ● |  |
|  | SEIINSK |  |  |  |  |  |
|  | TSSQPGFLER |  |  |  |  |  |
|  | YPEVGDLR |  |  |  |  |  |
|  | YSYNTEWR |  |  |  |  |  |
| TMEM97 | DPLLQEPPAWFK |  |  |  |  |  |
|  | ELYPVEFR | 28 | ● | A | ● | ● |
|  | GQRPETLHER |  |  |  |  |  |
| TNC | AAIDSYR |  |  |  |  |  |
|  | EDEGEITK |  |  |  |  |  |
|  | ESNPATINAATELDTPK |  |  |  |  |  |
|  | ETFTTGLDAPR |  |  | C |  |  |
|  | LDAPSQIEVK |  |  |  |  |  |
|  | LEELENLVSSLR |  |  | C |  |  |
|  | LNYSLPTG |  |  |  |  |  |
|  | RPETSYR |  |  |  |  |  |
|  | SIPVSAR |  |  |  |  |  |
|  | SQQATTK |  |  |  |  |  |
|  | VATYLPAPEGLK |  |  |  |  |  |
|  | VFAILENK |  |  |  |  |  |
|  | VSQTDNSITLEWR |  |  |  |  |  |
|  | YAPISGGDHAEVDVPK |  |  |  | ● | ● |
| TOMM22 | SAAGATFDLSLFVAQK |  |  |  |  |  |
| TPI1 | ADTDLQR |  |  |  |  |  |
|  | EAGITEK | 12.67 | ▲ | B | ● |  |
|  | FFVGGNWK | 7.1 | ▲ | B | ● |  |
|  | HVFGESDELIGQK | 65.81 | ● | A | ● |  |
|  | QSLGELIGTLNAAK | 43.67 | ● | A | ● |  |
|  | SNVSDAVAQSTR | 68.42 | ● | A | ● | ● |
|  | TATPQQAQEVHEK | 38.91 | ● | A | ● |  |
|  | VIADNVK | 32.23 | ● | A | ● |  |
|  | VVFEQTK | 16.35 | ▲ | B | ● |  |
|  | VVLAYEPVWAIGTGK | 50.2 | ● | A | ● |  |
| TRIP10 | AAQTAER |  |  |  |  |  |
|  | APSDSSLGTPSDGRPELR |  |  |  |  |  |
|  | AQQQLENGFK |  |  |  |  |  |
|  | EGGEGYVPTSYLR | 9.85 | ▲ | B | ● |  |
|  | ELVAENLSVR | 16.71 | ▲ | B | ● | ● |
|  | HTQWGLDLLDR |  |  |  |  |  |
|  | LDQDINATK |  |  |  |  |  |
|  | LQQQLEER |  |  |  |  |  |
|  | NDSHVLIELHK |  |  |  |  |  |
|  | NEYAAQLQR |  |  |  |  |  |
|  | TEVEQAYAK |  |  |  |  |  |
|  | VAANAVDPK |  |  |  |  |  |
|  | YEAWLAEAESR | 15.56 | ▲ | B | ● |  |
| TSN | EAVTEILGIEPDR |  |  |  |  |  |
|  | EHFGTVK |  |  |  |  |  |
|  | FPAEQYYR |  |  |  |  |  |
|  | THLTSLK |  |  |  |  |  |
|  | VEEVVYDLSIR |  |  |  |  |  |
|  | VVQSLEQTAR | 15 | ▲ | B | ● | ● |
| TXN | LEATINELV | 41 | ● | A | ● |  |
|  | TAFQEALDAAGDK | 61 | ● | A | ● | ● |
|  | VGEFSGANK | 53 | ● | A | ● |  |
| UBE2Z | FNPNFYR |  |  |  |  |  |
| UBL4A | HFSAADASR |  |  |  |  |  |
|  | LADSPPPQVWQLISK |  |  |  |  |  |
|  | LNLVVKPLEK |  |  |  |  |  |
|  | LSDYSIGPNSK | 11 | ▲ | B | ● | ● |
|  | LTLDDIER |  |  |  |  |  |
|  | VLEQLQR |  |  |  |  |  |
|  | VLLEEGEAQR |  |  |  |  |  |
| VAPB | EEGLSTR | 5.73 | ▲ | B | ● |  |
|  | GPFTDVVTTNLK |  |  |  |  |  |
|  | IISTTASK |  |  |  |  |  |
|  | LGNPTDR |  |  |  |  |  |
|  | LLALVVLFFIVGVIIGK |  |  |  |  |  |
|  | LQGEVQR | 11.49 | ▲ | B | ● |  |
|  | SLSSSLDDTEVK | 19.88 | ▲ | B | ● |  |
|  | TETPIVSK | 21.41 | ▲ | B | ● |  |
|  | TVQSNSPISALAPTGK | 32.65 | ● | A | ● | ● |
|  | VEQVLSLEPQHELK | 14.32 | ▲ | B | ● |  |
| VPS29 | ESYDYLK |  |  |  |  |  |
|  | FEAFEHENK |  |  |  |  |  |
|  | GDFDENLNYPEQK | 32 | ● | A | ● | ● |
|  | QFDVDILISGHTHK |  |  |  |  |  |
|  | TLAGDVHIVR |  |  |  |  |  |
|  | VVTVGQFK |  |  |  |  |  |
| VTN | AVRPGYPK |  |  |  |  |  |
|  | DVWGIEGPIDAAFTR |  |  | C |  |  |
|  | FEDGVLDPDYPR |  |  | C | ● | ● |
|  | NGSLFAFR |  |  |  |  |  |
|  | QPQFISR |  |  |  |  |  |
|  | VDTVDPPYPR |  |  |  |  |  |
| WASF2 | DVVGNDVATILSR | 24 | ▲ | B | ● |  |
|  | EEEVSLQGINTR | 19 | ▲ | B | ● |  |
|  | FYTDPSYFFDLWK |  |  |  |  |  |
|  | GSGLAGPK |  |  |  |  |  |
|  | QLGSLSK |  |  |  |  |  |
|  | QTLPSVR |  |  |  |  |  |
|  | SDLLSAIR | 27 | ▲ | B | ● | ● |
|  | SSLPAVSDAR |  |  |  |  |  |
|  | SSTIQDQK |  |  |  |  |  |
|  | VSSLAER |  |  |  |  |  |
|  | VTQLDPK |  |  |  |  |  |
| YWHAQ | AVTEQGAELSNEER | 84 | ● | A | ● | ● |
|  | VISSIEQK |  |  |  |  |  |
|  | YLIANATNPESK | 50 | ● | A | ● |  |

^1^: Chen, C. L. *et al.* Comparative Tissue Proteomics of Microdissected Specimens Reveals Novel Candidate Biomarkers of Bladder Cancer. *Mol. Cell Proteomics* **14**, 2466-2478, 2015.
