# Supplementary material for: Systematic verification of bladder cancer-associated tissue protein biomarker candidates in clinical urine specimens
Source: Oncotarget. 2018 Jul 20;9(56):30731–47. doi: 10.18632/oncotarget.24578 (PMC6089400; doi:10.18632/oncotarget.24578)
Supplement: Supplementary file 4 [file oncotarget-09-30731-s004.docx]

**Supplementary Table 3**: **The transition list of MRM assay of 122 bladder cancer associated tissue proteins**

| Proteins | Gene name | Dimethyl labeling | Dimethylated peptide sequence | Charge / ion | Q1 | Q3 | Predicted CE in MS method | Optimized CE | △CE | Retention time |
| --- | --- | --- | --- | --- | --- | --- | --- | --- | --- | --- |
| Alpha-2-macroglobulin-like protein 1 | A2ML1 | Heavy | [2MC]-ELSTVQESFLVK[2MC] | 2 / y5 | 724.44 | 627.43 | 37.00 | 43.00 | 6.00 | 33.39 |
|  |  |  |  | 2 / y6 | 724.44 | 756.47 | 37.00 | 37.00 | 0.00 | 33.39 |
|  |  |  |  | 2 / y7 | 724.44 | 884.53 | 37.00 | 34.00 | -3.00 | 33.39 |
|  |  | Light | [2Me]-ELSTVQESFLVK[2Me] | 2 / y5 | 718.41 | 621.4 | 37.00 | 43.00 | 6.27 | 33.39 |
|  |  |  |  | 2 / y6 | 718.41 | 750.44 | 37.00 | 37.00 | 0.27 | 33.39 |
|  |  |  |  | 2 / y7 | 718.41 | 878.5 | 37.00 | 34.00 | -3.00 | 33.39 |
| Isoform 1 of Acetoacetyl-CoA synthetase | AACS | Heavy | [2MC]-GIADVPEWFK[2MC] | 2 / y3 | 615.36 | 514.32 | 32.00 | 41.00 | 9.00 | 39.31 |
|  |  |  |  | 2 / y5 | 615.36 | 740.42 | 32.00 | 32.00 | 0.00 | 39.31 |
|  |  |  |  | 2 / y6 | 615.36 | 839.49 | 32.00 | 32.00 | 0.00 | 39.31 |
|  |  | Light | [2Me]-GIADVPEWFK[2Me] | 2 / y3 | 609.33 | 508.29 | 32.00 | 41.00 | 9.27 | 39.31 |
|  |  |  |  | 2 / y5 | 609.33 | 734.39 | 32.00 | 32.00 | 0.00 | 39.31 |
|  |  |  |  | 2 / y6 | 609.33 | 833.46 | 32.00 | 32.00 | 0.00 | 39.31 |
| Isoform 1 of Cytosolic acyl coenzyme A thioester hydrolase | ACOT7 | Heavy | [2MC]-ATLWYVPLSLK[2MC] | 2 / y5 | 679.94 | 591.43 | 35.00 | 41.00 | 6.00 | 43.19 |
|  |  |  |  | 2 / y6 | 679.94 | 690.5 | 35.00 | 35.00 | 0.00 | 43.19 |
|  |  |  |  | 2 / y7 | 679.94 | 853.56 | 35.00 | 32.00 | -3.00 | 43.19 |
|  |  | Light | [2Me]-ATLWYVPLSLK[2Me] | 2 / y5 | 673.91 | 585.4 | 35.00 | 41.00 | 6.27 | 43.19 |
|  |  |  |  | 2 / y6 | 673.91 | 684.47 | 35.00 | 35.00 | 0.27 | 43.19 |
|  |  |  |  | 2 / y7 | 673.91 | 847.53 | 35.00 | 32.00 | -3.00 | 43.19 |
| Isoform 2 of Peroxisomal acyl-coenzyme A oxidase 1 | ACOX1 | Heavy | [2MC]-WWPGGLGK[2MC] | 2 / y4 | 484.8 | 408.3 | 26.00 | 35.00 | 9.00 | 33.58 |
|  |  |  |  | 2 / y5 | 484.8 | 465.32 | 26.00 | 29.00 | 3.00 | 33.58 |
|  |  |  |  | 2 / y6 | 484.8 | 562.38 | 26.00 | 26.00 | 0.00 | 33.58 |
|  |  | Light | [2Me]-WWPGGLGK[2Me] | 2 / y4 | 478.77 | 402.27 | 26.00 | 35.00 | 9.27 | 33.58 |
|  |  |  |  | 2 / y5 | 478.77 | 459.29 | 26.00 | 29.00 | 3.27 | 33.58 |
|  |  |  |  | 2 / y6 | 478.77 | 556.35 | 26.00 | 26.00 | 0.00 | 33.58 |
| Actin, cytoplasmic 1 | ACTB | Heavy | [2MC]-SYELPDGQVITIGNER | 2 / b3 | 912.98 | 414.21 | 45.00 | 51.00 | 6.00 | 36.24 |
|  |  |  |  | 2 / b4 | 912.98 | 527.29 | 45.00 | 42.00 | -3.00 | 36.24 |
|  |  |  |  | 2 / y6 | 912.98 | 689.36 | 45.00 | 45.00 | 0.00 | 36.24 |
|  |  | Light | [2Me]-SYELPDGQVITIGNER | 2 / b3 | 909.97 | 408.18 | 45.00 | 51.00 | 6.13 | 36.24 |
|  |  |  |  | 2 / b4 | 909.97 | 521.26 | 45.00 | 42.00 | -2.87 | 36.24 |
|  |  |  |  | 2 / y6 | 909.97 | 689.36 | 45.00 | 45.00 | 0.13 | 36.24 |
| Adenylosuccinate synthetase isozyme 2 | ADSS | Heavy | [2MC]-FIEDELQIPVK[2MC] | 2 / b4 | 699.93 | 539.29 | 36.00 | 36.00 | 0.00 | 33.59 |
|  |  |  |  | 2 / b5 | 699.93 | 668.34 | 36.00 | 36.00 | 0.00 | 33.59 |
|  |  |  |  | 2 / y6 | 699.93 | 731.52 | 36.00 | 36.00 | 0.00 | 33.59 |
|  |  | Light | [2Me]-FIEDELQIPVK[2Me] | 2 / b4 | 693.9 | 533.26 | 36.00 | 36.00 | 0.27 | 33.59 |
|  |  |  |  | 2 / b5 | 693.9 | 662.3 | 36.00 | 36.00 | 0.27 | 33.59 |
|  |  |  |  | 2 / y6 | 693.9 | 725.49 | 36.00 | 36.00 | 0.00 | 33.59 |
| Agrin | AGRN | Heavy | [2MC]-GDFVSLALR | 2 / y5 | 506.31 | 559.36 | 27.00 | 27.00 | 0.00 | 34.55 |
|  |  |  |  | 2 / y7 | 506.31 | 805.49 | 27.00 | 27.00 | 0.00 | 34.55 |
|  |  |  |  | 2 / y8 | 506.31 | 920.52 | 27.00 | 24.00 | -3.00 | 34.55 |
|  |  | Light | [2Me]-GDFVSLALR | 2 / y5 | 503.29 | 559.36 | 27.00 | 27.00 | 0.13 | 34.55 |
|  |  |  |  | 2 / y7 | 503.29 | 805.49 | 27.00 | 27.00 | 0.00 | 34.55 |
|  |  |  |  | 2 / y8 | 503.29 | 920.52 | 27.00 | 24.00 | -3.00 | 34.55 |
| Adenosylhomocysteinase | AHCY | Heavy | [2MC]-WLNENAVEK[2MC] | 2 / y4 | 585.84 | 480.32 | 31.00 | 37.00 | 6.00 | 20.30 |
|  |  |  |  | 2 / y7 | 585.84 | 837.45 | 31.00 | 28.00 | -3.00 | 20.30 |
|  |  |  |  | 2 / y8 | 585.84 | 950.54 | 31.00 | 28.00 | -3.00 | 20.30 |
|  |  | Light | [2Me]-WLNENAVEK[2Me] | 2 / y4 | 579.81 | 474.29 | 31.00 | 37.00 | 6.27 | 20.30 |
|  |  |  |  | 2 / y7 | 579.81 | 831.42 | 31.00 | 28.00 | -3.00 | 20.30 |
|  |  |  |  | 2 / y8 | 579.81 | 944.5 | 31.00 | 28.00 | -3.00 | 20.30 |
| Ankyrin repeat and SAM domain-containing protein 1A | ANKS1A | Heavy | [2MC]-FETPLDLAALYGR | 2 / y6 | 750.42 | 650.36 | 38.00 | 44.00 | 6.00 | 44.68 |
|  |  |  |  | 2 / y7 | 750.42 | 763.45 | 38.00 | 47.00 | 9.00 | 44.68 |
|  |  |  |  | 2 / y8 | 750.42 | 878.47 | 38.00 | 41.00 | 3.00 | 44.68 |
|  |  | Light | [2Me]-FETPLDLAALYGR | 2 / y6 | 747.4 | 650.36 | 38.00 | 44.00 | 6.13 | 44.68 |
|  |  |  |  | 2 / y7 | 747.4 | 763.45 | 38.00 | 47.00 | 9.00 | 44.68 |
|  |  |  |  | 2 / y8 | 747.4 | 878.47 | 38.00 | 41.00 | 3.13 | 44.68 |
| Annexin A3 | ANXA3 | Heavy | [2MC]-GIGTDEFTLNR | 2 / y5 | 628.84 | 650.36 | 33.00 | 33.00 | 0.00 | 26.84 |
|  |  |  |  | 2 / y6 | 628.84 | 779.4 | 33.00 | 30.00 | -3.00 | 26.84 |
|  |  |  |  | 2 / y7 | 628.84 | 894.43 | 33.00 | 30.00 | -3.00 | 26.84 |
|  |  | Light | [2Me]-GIGTDEFTLNR | 2 / y5 | 625.82 | 650.36 | 33.00 | 33.00 | 0.00 | 26.84 |
|  |  |  |  | 2 / y6 | 625.82 | 779.4 | 33.00 | 30.00 | -3.00 | 26.84 |
|  |  |  |  | 2 / y7 | 625.82 | 894.43 | 33.00 | 30.00 | -3.00 | 26.84 |
| annexin A4 | ANXA4 | Heavy | [2MC]-QDAQDLYEAGEK[2MC] | 2 / y4 | 717.87 | 438.28 | 37.00 | 43.00 | 6.00 | 18.23 |
|  |  |  |  | 2 / y6 | 717.87 | 730.38 | 37.00 | 43.00 | 6.00 | 18.23 |
|  |  |  |  | 2 / y7 | 717.87 | 843.47 | 37.00 | 40.00 | 3.00 | 18.23 |
|  |  | Light | [2Me]-QDAQDLYEAGEK[2Me] | 2 / y4 | 711.84 | 432.25 | 36.00 | 43.00 | 6.27 | 18.23 |
|  |  |  |  | 2 / y6 | 711.84 | 724.35 | 36.00 | 43.00 | 6.27 | 18.23 |
|  |  |  |  | 2 / y7 | 711.84 | 837.44 | 36.00 | 40.00 | 4.00 | 18.23 |
| Isoform 1 of Amyloid-like protein 2 | APLP2 | Heavy | [2MC]-QQLVETHLAR | 2 / y6 | 614.86 | 726.39 | 33.00 | 35.00 | 2.51 | 23.28 |
|  |  |  |  | 2 / y7 | 614.86 | 825.46 | 32.00 | 35.00 | 3.00 | 23.28 |
|  |  |  |  | 2 / y8 | 614.86 | 938.54 | 32.00 | 32.00 | 0.00 | 23.28 |
|  |  | Light | [2Me]-QQLVETHLAR | 2 / y6 | 611.85 | 726.39 | 32.00 | 35.00 | 3.13 | 23.28 |
|  |  |  |  | 2 / y7 | 611.85 | 825.46 | 32.00 | 35.00 | 3.00 | 23.28 |
|  |  |  |  | 2 / y8 | 611.85 | 938.54 | 32.00 | 32.00 | 0.00 | 23.28 |
| Apolipoprotein B receptor | APOBR | Heavy | [2MC]-SWEQEEEEEEVR | 2 / b3 | 806.86 | 437.22 | 41.00 | 44.00 | 3.00 | 26.17 |
|  |  |  |  | 2 / b4 | 806.86 | 565.28 | 41.00 | 44.00 | 3.00 | 26.17 |
|  |  |  |  | 2 / y7 | 806.86 | 919.4 | 41.00 | 44.00 | 3.00 | 26.17 |
|  |  | Light | [2Me]-SWEQEEEEEEVR | 2 / b3 | 803.85 | 431.19 | 40.00 | 44.00 | 3.13 | 26.17 |
|  |  |  |  | 2 / b4 | 803.85 | 559.25 | 40.00 | 44.00 | 3.13 | 26.17 |
|  |  |  |  | 2 / y7 | 803.85 | 919.4 | 40.00 | 44.00 | 4.00 | 26.17 |
| ADP-ribosylation factor 4 | ARF4 | Heavy | [2MC]-DAVLLLFANK[2MC] | 2 / y4 | 586.39 | 513.32 | 31.00 | 25.00 | -6.00 | 42.89 |
|  |  |  |  | 2 / y5 | 586.39 | 626.41 | 31.00 | 28.00 | -3.00 | 42.89 |
|  |  |  |  | 2 / y6 | 586.39 | 739.49 | 31.00 | 25.00 | -6.00 | 42.89 |
|  |  | Light | [2Me]-DAVLLLFANK[2Me] | 2 / y4 | 580.36 | 507.29 | 31.00 | 25.00 | -5.74 | 42.89 |
|  |  |  |  | 2 / y5 | 580.36 | 620.38 | 31.00 | 28.00 | -2.74 | 42.89 |
|  |  |  |  | 2 / y6 | 580.36 | 733.46 | 31.00 | 25.00 | -6.00 | 42.89 |
| Isoform 5 of Brain-specific angiogenesis inhibitor 1-associated protein 2 | BAIAP2 | Heavy | [2MC]-ALAGVTYAAK[2MC] | 2 / y7 | 516.84 | 743.45 | 28.00 | 25.00 | -3.00 | 27.70 |
|  |  |  |  | 2 / y8 | 516.84 | 814.49 | 28.00 | 25.00 | -3.00 | 27.70 |
|  |  |  |  | 2 / y9 | 516.84 | 927.57 | 28.00 | 25.00 | -3.00 | 27.70 |
|  |  | Light | [2Me]-ALAGVTYAAK[2Me] | 2 / y7 | 510.81 | 737.42 | 27.00 | 25.00 | -2.00 | 27.70 |
|  |  |  |  | 2 / y8 | 510.81 | 808.46 | 27.00 | 25.00 | -2.00 | 27.70 |
|  |  |  |  | 2 / y9 | 510.81 | 921.54 | 27.00 | 25.00 | -2.00 | 27.70 |
| Isoform 1 of BRCA2 and CDKN1A-interacting protein | BCCIP | Heavy | [2MC]-LLQQLFLK[2MC] | 2 / y3 | 535.88 | 441.33 | 29.00 | 38.00 | 9.00 | 34.25 |
|  |  |  |  | 2 / y6 | 535.88 | 810.53 | 29.00 | 26.00 | -3.00 | 34.25 |
|  |  |  |  | 2 / y7 | 535.88 | 923.61 | 29.00 | 26.00 | -3.00 | 34.25 |
|  |  | Light | [2Me]-LLQQLFLK[2Me] | 2 / y3 | 529.85 | 435.3 | 28.00 | 38.00 | 9.27 | 34.25 |
|  |  |  |  | 2 / y6 | 529.85 | 804.5 | 28.00 | 26.00 | -2.00 | 34.25 |
|  |  |  |  | 2 / y7 | 529.85 | 917.58 | 28.00 | 26.00 | -2.00 | 34.25 |
| BRO1 domain-containing protein BROX | BROX | Heavy | [2MC]-IPTEAPQLELK[2MC] | 2 / y6 | 653.92 | 761.5 | 34.00 | 43.00 | 9.00 | 29.49 |
|  |  |  |  | 2 / y7 | 653.92 | 832.54 | 34.00 | 40.00 | 6.00 | 29.49 |
|  |  |  |  | 2 / y8 | 653.92 | 961.58 | 34.00 | 37.00 | 3.00 | 29.49 |
|  |  | Light | [2Me]-IPTEAPQLELK[2Me] | 2 / y6 | 647.88 | 755.47 | 34.00 | 43.00 | 9.00 | 29.49 |
|  |  |  |  | 2 / y7 | 647.88 | 826.5 | 34.00 | 40.00 | 6.00 | 29.49 |
|  |  |  |  | 2 / y8 | 647.88 | 955.55 | 34.00 | 37.00 | 3.27 | 29.49 |
| Isoform Long of ES1 protein homolog, mitochondrial | C21orf33 | Heavy | [2MC]-EVVEAHVDQK[2MC] | 2 / y6 | 611.36 | 731.43 | 32.00 | 35.00 | 3.00 | 33.08 |
|  |  |  |  | 2 / y7 | 611.36 | 860.47 | 32.00 | 35.00 | 3.00 | 33.08 |
|  |  |  |  | 2 / y8 | 611.36 | 959.54 | 32.00 | 35.00 | 3.10 | 33.08 |
|  |  | Light | [2Me]-EVVEAHVDQK[2Me] | 2 / y6 | 605.33 | 725.39 | 32.00 | 35.00 | 3.00 | 33.08 |
|  |  |  |  | 2 / y7 | 605.33 | 854.44 | 32.00 | 35.00 | 3.00 | 33.08 |
|  |  |  |  | 2 / y8 | 605.33 | 953.51 | 32.00 | 35.00 | 3.37 | 33.08 |
| Calmodulin | CALM3 | Heavy | [2MC]-DTDSEEEIR | 2 / y6 | 564.27 | 762.36 | 30.00 | 33.00 | 3.00 | 21.70 |
|  |  |  |  | 2 / y7 | 564.27 | 877.39 | 30.00 | 27.00 | -3.00 | 21.70 |
|  |  |  |  | 2 / y8 | 564.27 | 978.44 | 30.00 | 27.00 | -3.00 | 21.70 |
|  |  | Light | [2Me]-DTDSEEEIR | 2 / y6 | 561.25 | 762.36 | 30.00 | 33.00 | 3.00 | 21.70 |
|  |  |  |  | 2 / y7 | 561.25 | 877.39 | 30.00 | 27.00 | -3.00 | 21.70 |
|  |  |  |  | 2 / y8 | 561.25 | 978.44 | 30.00 | 27.00 | -3.00 | 21.70 |
| Complement factor H-related protein | CFHR | Heavy | [2MC]-INHGILYDEEK[2MC] | 2 / y3 | 699.9 | 439.26 | 36.00 | 45.00 | 9.00 | 21.54 |
|  |  |  |  | 2 / y5 | 699.9 | 717.35 | 36.00 | 45.00 | 9.00 | 21.54 |
|  |  |  |  | 2 / y6 | 699.9 | 830.44 | 36.00 | 39.00 | 3.00 | 21.54 |
|  |  | Light | [2Me]-INHGILYDEEK[2Me] | 2 / y3 | 693.87 | 433.23 | 36.00 | 45.00 | 9.27 | 21.54 |
|  |  |  |  | 2 / y5 | 693.87 | 711.32 | 36.00 | 45.00 | 9.27 | 21.54 |
|  |  |  |  | 2 / y6 | 693.87 | 824.4 | 36.00 | 39.00 | 3.00 | 21.54 |
| Isoform Brain of Clathrin light chain A | CLTA | Heavy | [2MC]-LQSEPESIR | 2 / y5 | 546.81 | 601.33 | 29.00 | 38.00 | 9.00 | 15.86 |
|  |  |  |  | 2 / y7 | 546.81 | 817.41 | 29.00 | 29.00 | 0.00 | 15.86 |
|  |  |  |  | 2 / y8 | 546.81 | 945.46 | 29.00 | 26.00 | -3.00 | 15.86 |
|  |  | Light | [2Me]-LQSEPESIR | 2 / y5 | 543.79 | 601.33 | 29.00 | 38.00 | 9.00 | 15.86 |
|  |  |  |  | 2 / y7 | 543.79 | 817.41 | 29.00 | 29.00 | 0.00 | 15.86 |
|  |  |  |  | 2 / y8 | 543.79 | 945.46 | 29.00 | 26.00 | -3.00 | 15.86 |
| Isoform 4 of Collagen alpha-1(XII) chain | COL12A1 | Heavy | [2MC]-DELLAAIK[2MC] | 2 / y4 | 470.82 | 436.33 | 26.00 | 26.00 | 0.00 | 31.03 |
|  |  |  |  | 2 / y5 | 470.82 | 549.42 | 26.00 | 26.00 | 0.00 | 31.03 |
|  |  |  |  | 2 / y7 | 470.82 | 791.54 | 26.00 | 26.00 | 0.00 | 31.03 |
|  |  | Light | [2Me]-DELLAAIK[2Me] | 2 / y4 | 464.79 | 430.3 | 25.00 | 26.00 | 0.27 | 31.03 |
|  |  |  |  | 2 / y5 | 464.79 | 543.39 | 25.00 | 26.00 | 0.27 | 31.03 |
|  |  |  |  | 2 / y7 | 464.79 | 785.51 | 25.00 | 26.00 | 1.00 | 31.03 |
| Collagen alpha-2(IV) chain | COL4A2 | Heavy | [2MC]-GGVSAVPGFR | 2 / y4 | 490.79 | 476.26 | 27.00 | 24.00 | -3.00 | 24.84 |
|  |  |  |  | 2 / y7 | 490.79 | 733.4 | 27.00 | 27.00 | 0.00 | 24.84 |
|  |  |  |  | 2 / y9 | 490.79 | 889.49 | 27.00 | 24.00 | -3.00 | 24.84 |
|  |  | Light | [2Me]-GGVSAVPGFR | 2 / y4 | 487.77 | 476.26 | 26.00 | 24.00 | -2.87 | 24.84 |
|  |  |  |  | 2 / y7 | 487.77 | 733.4 | 26.00 | 27.00 | 1.00 | 24.84 |
|  |  |  |  | 2 / y9 | 487.77 | 889.49 | 26.00 | 24.00 | -2.87 | 24.84 |
| Isoform Membrane-bound of Catechol O-methyltransferase | COMT | Heavy | [2MC]-VTLVVGASQDIIPQLK[2MC] | 2 / b4 | 875.06 | 447.34 | 44.00 | 41.00 | -3.00 | 41.05 |
|  |  |  |  | 2 / b5 | 875.06 | 546.41 | 44.00 | 41.00 | -3.00 | 41.05 |
|  |  |  |  | 2 / y4 | 875.06 | 519.37 | 44.00 | 47.00 | 3.00 | 41.05 |
|  |  | Light | [2Me]-VTLVVGASQDIIPQLK[2Me] | 2 / b4 | 869.03 | 441.31 | 43.00 | 41.00 | -2.73 | 41.05 |
|  |  |  |  | 2 / b5 | 869.03 | 540.38 | 43.00 | 41.00 | -2.73 | 41.05 |
|  |  |  |  | 2 / y4 | 869.03 | 513.34 | 43.00 | 47.00 | 3.27 | 41.05 |
| Cytochrome P450 1A1 | CYP1A1 | Heavy | [2MC]-IGSTPVVVLSGLDTIR | 2 / y3 | 831.01 | 389.25 | 42.00 | 57.00 | 15.00 | 42.28 |
|  |  |  |  | 2 / y8 | 831.01 | 874.5 | 42.00 | 39.00 | -3.00 | 42.28 |
|  |  |  |  | 2 / y9 | 831.01 | 973.57 | 42.00 | 39.00 | -3.00 | 42.28 |
|  |  | Light | [2Me]-IGSTPVVVLSGLDTIR | 2 / y3 | 827.99 | 389.25 | 41.00 | 57.00 | 15.13 | 42.28 |
|  |  |  |  | 2 / y8 | 827.99 | 874.5 | 41.00 | 39.00 | -2.87 | 42.28 |
|  |  |  |  | 2 / y9 | 827.99 | 973.57 | 41.00 | 39.00 | -2.00 | 42.28 |
| dCTP pyrophosphatase 1 | DCTPP1 | Heavy | [2MC]-VDLPLAVLSK[2MC] | 2 / y7 | 561.89 | 761.53 | 30.00 | 30.00 | 0.00 | 38.46 |
|  |  |  |  | 2 / y8 | 561.89 | 874.62 | 30.00 | 27.00 | -3.00 | 38.46 |
|  |  |  |  | 2 / y9 | 561.89 | 989.65 | 30.00 | 27.00 | -3.00 | 38.46 |
|  |  | Light | [2Me]-VDLPLAVLSK[2Me] | 2 / y7 | 555.86 | 755.5 | 29.00 | 30.00 | 1.00 | 38.46 |
|  |  |  |  | 2 / y8 | 555.86 | 868.59 | 29.00 | 27.00 | -2.00 | 38.46 |
|  |  |  |  | 2 / y9 | 555.86 | 983.61 | 29.00 | 27.00 | -2.00 | 38.46 |
| D-dopachrome decarboxylase | DDT | Heavy | [2MC]-FFPLESWQIGK[2MC] | 2 / y5 | 710.42 | 665.42 | 36.00 | 45.00 | 9.00 | 43.04 |
|  |  |  |  | 2 / y6 | 710.42 | 752.45 | 36.00 | 42.00 | 6.00 | 43.04 |
|  |  |  |  | 2 / y7 | 710.42 | 881.49 | 36.00 | 39.00 | 3.00 | 43.04 |
|  |  | Light | [2Me]-FFPLESWQIGK[2Me] | 2 / y5 | 704.39 | 659.39 | 36.00 | 45.00 | 9.27 | 43.04 |
|  |  |  |  | 2 / y6 | 704.39 | 746.42 | 36.00 | 42.00 | 6.00 | 43.04 |
|  |  |  |  | 2 / y7 | 704.39 | 875.46 | 36.00 | 39.00 | 3.00 | 43.04 |
| Isoform 1 of ATP-dependent RNA helicase DDX19B | DDX19B | Heavy | [2MC]-SNLVDNTNQVEVLQR | 2 / b4 | 881.98 | 448.3 | 44.00 | 44.00 | 0.00 | 25.74 |
|  |  |  |  | 2 / y3 | 881.98 | 416.26 | 44.00 | 44.00 | 0.00 | 25.74 |
|  |  |  |  | 2 / y4 | 881.98 | 515.33 | 44.00 | 50.00 | 6.00 | 25.74 |
|  |  | Light | [2Me]-SNLVDNTNQVEVLQR | 2 / b4 | 878.96 | 442.27 | 44.00 | 44.00 | 0.13 | 25.74 |
|  |  |  |  | 2 / y3 | 878.96 | 416.26 | 44.00 | 44.00 | 0.13 | 25.74 |
|  |  |  |  | 2 / y4 | 878.96 | 515.33 | 44.00 | 50.00 | 6.13 | 25.74 |
| DnaJ homolog subfamily B member 11 | DNAJB11 | Heavy | [2MC]-TLEVEIEPGVR | 2 / b3 | 638.37 | 378.24 | 33.00 | 36.00 | 3.00 | 29.96 |
|  |  |  |  | 2 / y4 | 638.37 | 428.26 | 33.00 | 45.00 | 12.00 | 29.96 |
|  |  |  |  | 2 / y6 | 638.37 | 670.39 | 33.00 | 36.00 | 3.00 | 29.96 |
|  |  | Light | [2Me]-TLEVEIEPGVR | 2 / b3 | 635.36 | 372.21 | 33.00 | 36.00 | 3.13 | 29.96 |
|  |  |  |  | 2 / y4 | 635.36 | 428.26 | 33.00 | 45.00 | 12.13 | 29.96 |
|  |  |  |  | 2 / y6 | 635.36 | 670.39 | 33.00 | 36.00 | 3.00 | 29.96 |
| Isoform 5 of Dynamin-1-like protein | DNM1L | Heavy | [2MC]-SATLLQLITK[2MC] | 2 / y5 | 578.4 | 636.45 | 30.00 | 30.00 | 0.00 | 40.42 |
|  |  |  |  | 2 / y6 | 578.4 | 749.53 | 30.00 | 33.00 | 3.00 | 40.42 |
|  |  |  |  | 2 / y8 | 578.4 | 963.67 | 30.00 | 30.00 | 0.00 | 40.42 |
|  |  | Light | [2Me]-SATLLQLITK[2Me] | 2 / y5 | 572.37 | 630.42 | 30.00 | 30.00 | 0.27 | 40.42 |
|  |  |  |  | 2 / y6 | 572.37 | 743.5 | 30.00 | 33.00 | 3.00 | 40.42 |
|  |  |  |  | 2 / y8 | 572.37 | 957.63 | 30.00 | 30.00 | 0.00 | 40.42 |
| Dipeptidyl peptidase 2 | DPP7 | Heavy | [2MC]-SLPFGAQSTQR | 2 / y7 | 613.34 | 747.37 | 32.00 | 35.00 | 3.00 | 23.25 |
|  |  |  |  | 2 / y8 | 613.34 | 894.44 | 32.00 | 35.00 | 3.00 | 23.25 |
|  |  |  |  | 2 / y9 | 613.34 | 991.5 | 32.00 | 29.00 | -3.00 | 23.25 |
|  |  | Light | [2Me]-SLPFGAQSTQR | 2 / y7 | 610.33 | 747.37 | 32.00 | 35.00 | 3.00 | 23.25 |
|  |  |  |  | 2 / y8 | 610.33 | 894.44 | 32.00 | 35.00 | 3.00 | 23.25 |
|  |  |  |  | 2 / y9 | 610.33 | 991.5 | 32.00 | 29.00 | -3.00 | 23.25 |
| Protein dpy-30 homolog | DPY30 | Heavy | [2MC]-VDLQSLPTR | 2 / y5 | 531.82 | 573.34 | 28.00 | 28.00 | 0.00 | 23.93 |
|  |  |  |  | 2 / y7 | 531.82 | 814.48 | 28.00 | 25.00 | -3.00 | 23.93 |
|  |  |  |  | 2 / y8 | 531.82 | 929.51 | 28.00 | 25.00 | -3.00 | 23.93 |
|  |  | Light | [2Me]-VDLQSLPTR | 2 / y5 | 528.81 | 573.34 | 28.00 | 28.00 | 0.13 | 23.93 |
|  |  |  |  | 2 / y7 | 528.81 | 814.48 | 28.00 | 25.00 | -3.00 | 23.93 |
|  |  |  |  | 2 / y8 | 528.81 | 929.51 | 28.00 | 25.00 | -3.00 | 23.93 |
| Dynein light chain roadblock-type 2 | DYNLRB2 | Heavy | [2MC]-DIDPQNDLTFLR | 2 / y4 | 740.9 | 536.32 | 38.00 | 47.00 | 9.00 | 37.80 |
|  |  |  |  | 2 / y5 | 740.9 | 649.4 | 38.00 | 47.00 | 9.00 | 37.80 |
|  |  |  |  | 2 / y7 | 740.9 | 878.47 | 38.00 | 41.00 | 3.00 | 37.80 |
|  |  | Light | [2Me]-DIDPQNDLTFLR | 2 / y4 | 737.88 | 536.32 | 37.00 | 47.00 | 9.13 | 37.80 |
|  |  |  |  | 2 / y5 | 737.88 | 649.4 | 37.00 | 47.00 | 9.13 | 37.80 |
|  |  |  |  | 2 / y7 | 737.88 | 878.47 | 37.00 | 41.00 | 4.00 | 37.80 |
| 3-beta-hydroxysteroid-Delta(8),Delta(7)-isomerase | EBP | Heavy | [2MC]-AAVVPLGTWR | 2 / y6 | 552.34 | 729.4 | 29.00 | 26.00 | -3.00 | 30.95 |
|  |  |  |  | 2 / y7 | 552.34 | 828.47 | 29.00 | 29.00 | 0.00 | 30.95 |
|  |  |  |  | 2 / y8 | 552.34 | 927.54 | 29.00 | 26.00 | -3.00 | 30.95 |
|  |  | Light | [2Me]-AAVVPLGTWR | 2 / y6 | 549.33 | 729.4 | 29.00 | 26.00 | -3.00 | 30.95 |
|  |  |  |  | 2 / y7 | 549.33 | 828.47 | 29.00 | 29.00 | 0.00 | 30.95 |
|  |  |  |  | 2 / y8 | 549.33 | 927.54 | 29.00 | 26.00 | -3.00 | 30.95 |
| Isoform 1 of Epidermal growth factor receptor | EGFR | Heavy | [2MC]-NYDLSFLK[2MC] | 2 / y4 | 534.32 | 528.36 | 29.00 | 32.00 | 3.00 | 34.89 |
|  |  |  |  | 2 / y5 | 534.32 | 641.44 | 29.00 | 29.00 | 0.00 | 34.89 |
|  |  |  |  | 2 / y7 | 534.32 | 919.53 | 29.00 | 23.00 | -6.00 | 34.89 |
|  |  | Light | [2Me]-NYDLSFLK[2Me] | 2 / y4 | 528.29 | 522.33 | 28.00 | 32.00 | 3.27 | 34.89 |
|  |  |  |  | 2 / y5 | 528.29 | 635.41 | 28.00 | 29.00 | 1.00 | 34.89 |
|  |  |  |  | 2 / y7 | 528.29 | 913.5 | 28.00 | 23.00 | -5.00 | 34.89 |
| Peroxisomal bifunctional enzyme | EHHADH | Heavy | [2MC]-WSTPSGASWK[2MC] | 2 / y3 | 587.83 | 454.29 | 31.00 | 34.00 | 3.00 | 26.81 |
|  |  |  |  | 2 / y5 | 587.83 | 582.35 | 31.00 | 37.00 | 6.00 | 26.81 |
|  |  |  |  | 2 / y9 | 587.83 | 954.51 | 31.00 | 31.00 | 0.00 | 26.81 |
|  |  | Light | [2Me]-WSTPSGASWK[2Me] | 2 / y3 | 581.8 | 448.26 | 31.00 | 34.00 | 3.27 | 26.81 |
|  |  |  |  | 2 / y5 | 581.8 | 576.31 | 31.00 | 37.00 | 6.27 | 26.81 |
|  |  |  |  | 2 / y9 | 581.8 | 948.48 | 31.00 | 31.00 | 0.00 | 26.81 |
| ELMO domain-containing protein 2 | ELMOD2 | Heavy | [2MC]-ATHVVQSEVDK[2MC] | 2 / y5 | 640.88 | 611.35 | 33.00 | 39.00 | 6.00 | 16.29 |
|  |  |  |  | 2 / y7 | 640.88 | 838.47 | 33.00 | 33.00 | 0.00 | 16.29 |
|  |  |  |  | 2 / y8 | 640.88 | 937.54 | 33.00 | 36.00 | 3.00 | 16.29 |
|  |  | Light | [2Me]-ATHVVQSEVDK[2Me] | 2 / y5 | 634.85 | 605.31 | 33.00 | 39.00 | 6.27 | 16.29 |
|  |  |  |  | 2 / y7 | 634.85 | 832.44 | 33.00 | 33.00 | 0.00 | 16.29 |
|  |  |  |  | 2 / y8 | 634.85 | 931.51 | 33.00 | 36.00 | 3.00 | 16.29 |
| Isoform alpha-enolase of Alpha-enolase | ENO1 | Heavy | [2MC]-EGLELLK[2MC] | 2 / y3 | 435.3 | 407.34 | 24.00 | 27.00 | 3.00 | 27.87 |
|  |  |  |  | 2 / y4 | 435.3 | 536.39 | 24.00 | 24.00 | 0.00 | 27.87 |
|  |  |  |  | 2 / y6 | 435.3 | 706.49 | 24.00 | 21.00 | -3.00 | 27.87 |
|  |  | Light | [2Me]-EGLELLK[2Me] | 2 / y3 | 429.27 | 401.31 | 24.00 | 27.00 | 3.27 | 27.87 |
|  |  |  |  | 2 / y4 | 429.27 | 530.35 | 24.00 | 24.00 | 0.15 | 27.87 |
|  |  |  |  | 2 / y6 | 429.27 | 700.46 | 24.00 | 21.00 | -3.00 | 27.87 |
| Fatty acid synthase | FASN | Heavy | [2MC]-VFTTVGSAEK[2MC] | 2 / y5 | 553.84 | 525.31 | 29.00 | 38.00 | 9.00 | 17.80 |
|  |  |  |  | 2 / y8 | 553.84 | 826.47 | 29.00 | 26.00 | -3.00 | 17.80 |
|  |  |  |  | 2 / y9 | 553.84 | 973.54 | 29.00 | 26.00 | -3.00 | 17.80 |
|  |  | Light | [2Me]-VFTTVGSAEK[2Me] | 2 / y5 | 547.81 | 519.28 | 29.00 | 38.00 | 9.27 | 17.80 |
|  |  |  |  | 2 / y8 | 547.81 | 820.44 | 29.00 | 26.00 | -3.00 | 17.80 |
|  |  |  |  | 2 / y9 | 547.81 | 967.51 | 29.00 | 26.00 | -3.00 | 17.80 |
| Isoform B of Fibulin-1 | FBLN1_Isoform B | Heavy | [2MC]-EFTRPEEIIFLR | 2 / b4 | 792.45 | 568.33 | 40.00 | 43.00 | 3.00 | 37.85 |
|  |  |  |  | 2 / b7 | 792.45 | 923.47 | 40.00 | 55.00 | 15.00 | 37.85 |
|  |  |  |  | 2 / y5 | 792.45 | 661.44 | 40.00 | 52.00 | 12.00 | 37.85 |
|  |  | Light | [2Me]-EFTRPEEIIFLR | 2 / b4 | 789.44 | 562.3 | 40.00 | 43.00 | 3.13 | 37.85 |
|  |  |  |  | 2 / b7 | 789.44 | 917.44 | 40.00 | 55.00 | 15.13 | 37.85 |
|  |  |  |  | 2 / y5 | 789.44 | 661.44 | 40.00 | 52.00 | 12.13 | 37.85 |
| Isoform C of Fibulin-1 | FBLN1_Isoform C | Heavy | [2MC]-GYHLNEEGTR | 2 / y6 | 605.31 | 705.32 | 32.00 | 35.00 | 3.00 | 11.50 |
|  |  |  |  | 2 / y7 | 605.31 | 818.4 | 32.00 | 35.00 | 3.00 | 11.50 |
|  |  |  |  | 2 / y8 | 605.31 | 955.46 | 32.00 | 35.00 | 3.00 | 11.50 |
|  |  | Light | [2Me]-GYHLNEEGTR | 2 / y6 | 602.29 | 705.32 | 32.00 | 35.00 | 3.13 | 11.50 |
|  |  |  |  | 2 / y7 | 602.29 | 818.4 | 32.00 | 35.00 | 3.00 | 11.50 |
|  |  |  |  | 2 / y8 | 602.29 | 955.46 | 32.00 | 35.00 | 3.00 | 11.50 |
| glucan 1, 4-alpha-glucosidase | GAA | Heavy | [2MC]-WGYSSTAITR | 2 / y4 | 588.32 | 460.29 | 31.00 | 34.00 | 3.00 | 23.51 |
|  |  |  |  | 2 / y5 | 588.32 | 561.34 | 31.00 | 34.00 | 3.00 | 23.51 |
|  |  |  |  | 2 / y9 | 588.32 | 955.48 | 31.00 | 31.00 | 0.00 | 23.51 |
|  |  | Light | [2Me]-WGYSSTAITR | 2 / y4 | 585.3 | 460.29 | 31.00 | 34.00 | 3.13 | 23.51 |
|  |  |  |  | 2 / y5 | 585.3 | 561.34 | 31.00 | 34.00 | 3.13 | 23.51 |
|  |  |  |  | 2 / y9 | 585.3 | 955.48 | 31.00 | 31.00 | 0.00 | 23.51 |
| glycyl-tRNA synthetase, isoform CRA_b | GARS | Heavy | [2MC]-TVNVVQFEPSK[2MC] | 2 / b4 | 658.4 | 448.3 | 34.00 | 31.00 | -3.00 | 35.20 |
|  |  |  |  | 2 / y5 | 658.4 | 641.37 | 34.00 | 34.00 | 0.00 | 35.20 |
|  |  |  |  | 2 / y7 | 658.4 | 868.5 | 34.00 | 34.00 | 0.00 | 35.20 |
|  |  | Light | [2Me]-TVNVVQFEPSK[2Me] | 2 / b4 | 652.37 | 442.27 | 34.00 | 31.00 | -2.73 | 35.20 |
|  |  |  |  | 2 / y5 | 652.37 | 635.34 | 34.00 | 34.00 | 0.27 | 35.20 |
|  |  |  |  | 2 / y7 | 652.37 | 862.47 | 34.00 | 34.00 | 0.00 | 35.20 |
| Isoform Long of Trifunctional purine biosynthetic protein adenosine-3 | GART | Heavy | [2MC]-GDTVATLSER | 2 / y5 | 541.8 | 605.33 | 29.00 | 29.00 | 0.00 | 17.57 |
|  |  |  |  | 2 / y6 | 541.8 | 676.36 | 29.00 | 29.00 | 0.00 | 17.57 |
|  |  |  |  | 2 / y8 | 541.8 | 876.48 | 29.00 | 26.00 | -3.00 | 17.57 |
|  |  | Light | [2Me]-GDTVATLSER | 2 / y5 | 538.78 | 605.33 | 29.00 | 29.00 | 0.13 | 17.57 |
|  |  |  |  | 2 / y6 | 538.78 | 676.36 | 29.00 | 29.00 | 0.00 | 17.57 |
|  |  |  |  | 2 / y8 | 538.78 | 876.48 | 29.00 | 26.00 | -3.00 | 17.57 |
| Gamma-glutamyl hydrolase | GGH | Heavy | [2MC]-FFNVLTTNTDGK[2MC] | 2 / b3 | 712.91 | 443.25 | 36.00 | 39.00 | 3.00 | 29.72 |
|  |  |  |  | 2 / b4 | 712.91 | 542.32 | 36.00 | 36.00 | 0.00 | 29.72 |
|  |  |  |  | 2 / y8 | 712.91 | 883.49 | 36.00 | 33.00 | -3.00 | 29.72 |
|  |  | Light | [2Me]-FFNVLTTNTDGK[2Me] | 2 / b3 | 706.87 | 437.22 | 36.00 | 39.00 | 3.27 | 29.72 |
|  |  |  |  | 2 / b4 | 706.87 | 536.29 | 36.00 | 36.00 | 0.27 | 29.72 |
|  |  |  |  | 2 / y8 | 706.87 | 877.46 | 36.00 | 33.00 | -3.00 | 29.72 |
| cDNA FLJ53927, highly similar to Beta-hexosaminidase alpha chain | HEXA | Heavy | [2MC]-IQPDTIIQVWR | 2 / y5 | 701.92 | 701.41 | 36.00 | 21.00 | -15.00 | 39.59 |
|  |  |  |  | 2 / y6 | 701.92 | 814.49 | 36.00 | 42.00 | 6.00 | 39.59 |
|  |  |  |  | 2 / y7 | 701.92 | 915.54 | 36.00 | 42.00 | 6.00 | 39.59 |
|  |  | Light | [2Me]-IQPDTIIQVWR | 2 / y5 | 698.9 | 701.41 | 36.00 | 21.00 | -14.87 | 39.59 |
|  |  |  |  | 2 / y6 | 698.9 | 814.49 | 36.00 | 42.00 | 6.13 | 39.59 |
|  |  |  |  | 2 / y7 | 698.9 | 915.54 | 36.00 | 42.00 | 6.00 | 39.59 |
| HLA class I histocompatibility antigen, A-33 alpha chain | HLA-A | Heavy | [2MC]-FIAVGYVDDTQFVR | 2 / b4 | 832.45 | 465.33 | 42.00 | 39.00 | -3.00 | 45.00 |
|  |  |  |  | 2 / b5 | 832.45 | 522.35 | 42.00 | 42.00 | 0.00 | 45.00 |
|  |  |  |  | 2 / y5 | 832.45 | 650.36 | 42.00 | 48.00 | 6.00 | 45.00 |
|  |  | Light | [2Me]-FIAVGYVDDTQFVR | 2 / b4 | 829.43 | 459.3 | 41.00 | 39.00 | -2.87 | 45.00 |
|  |  |  |  | 2 / b5 | 829.43 | 516.32 | 41.00 | 42.00 | 0.13 | 45.00 |
|  |  |  |  | 2 / y5 | 829.43 | 650.36 | 41.00 | 48.00 | 6.13 | 45.00 |
| Hypoxanthine-guanine phosphoribosyltransferase | HPRT1 | Heavy | [2MC]-VIGGDDLSTLTGK[2MC] | 2 / y6 | 672.41 | 640.41 | 35.00 | 35.00 | 0.00 | 27.93 |
|  |  |  |  | 2 / y7 | 672.41 | 753.49 | 35.00 | 41.00 | 6.00 | 27.93 |
|  |  |  |  | 2 / y8 | 672.41 | 868.52 | 35.00 | 32.00 | -3.00 | 27.93 |
|  |  | Light | [2Me]-VIGGDDLSTLTGK[2Me] | 2 / y6 | 666.37 | 634.38 | 34.00 | 35.00 | 0.27 | 27.93 |
|  |  |  |  | 2 / y7 | 666.37 | 747.46 | 34.00 | 41.00 | 7.00 | 27.93 |
|  |  |  |  | 2 / y8 | 666.37 | 862.49 | 34.00 | 32.00 | -2.00 | 27.93 |
| Heat shock protein HSP 90-beta | HSP90AB1 | Heavy | [2MC]-ALLFIPR | 2 / y4 | 432.3 | 532.32 | 24.00 | 21.00 | -3.00 | 36.96 |
|  |  |  |  | 2 / y5 | 432.3 | 645.41 | 24.00 | 21.00 | -3.00 | 36.96 |
|  |  |  |  | 2 / y6 | 432.3 | 758.49 | 24.00 | 21.00 | -3.00 | 36.96 |
|  |  | Light | [2Me]-ALLFIPR | 2 / y4 | 429.28 | 532.32 | 24.00 | 21.00 | -2.98 | 36.96 |
|  |  |  |  | 2 / y5 | 429.28 | 645.41 | 24.00 | 21.00 | -3.00 | 36.96 |
|  |  |  |  | 2 / y6 | 429.28 | 758.49 | 24.00 | 21.00 | -3.00 | 36.96 |
| 60 kDa heat shock protein, mitochondrial | HSPD1 | Heavy | [2MC]-VGLQVVAVK[2MC] | 2 / y4 | 490.86 | 450.35 | 27.00 | 33.00 | 6.00 | 34.50 |
|  |  |  |  | 2 / y5 | 490.86 | 549.42 | 27.00 | 27.00 | 0.00 | 34.50 |
|  |  |  |  | 2 / y8 | 490.86 | 847.58 | 27.00 | 24.00 | -3.00 | 34.50 |
|  |  | Light | [2Me]-VGLQVVAVK[2Me] | 2 / y4 | 484.83 | 444.32 | 26.00 | 33.00 | 6.27 | 34.50 |
|  |  |  |  | 2 / y5 | 484.83 | 543.39 | 26.00 | 27.00 | 0.67 | 34.50 |
|  |  |  |  | 2 / y8 | 484.83 | 841.55 | 26.00 | 24.00 | -2.00 | 34.50 |
| 10 kDa heat shock protein, mitochondrial | HSPE1 | Heavy | [2MC]-VLQATVVAVGSGSK[2MC] | 2 / y5 | 692.45 | 469.28 | 35.00 | 32.00 | -3.00 | 22.82 |
|  |  |  |  | 2 / y7 | 692.45 | 639.39 | 35.00 | 35.00 | 0.00 | 22.82 |
|  |  |  |  | 2 / y8 | 692.45 | 738.46 | 35.00 | 32.00 | -3.00 | 22.82 |
|  |  | Light | [2Me]-VLQATVVAVGSGSK[2Me] | 2 / y5 | 686.41 | 463.25 | 35.00 | 32.00 | -2.73 | 22.82 |
|  |  |  |  | 2 / y7 | 686.41 | 633.36 | 35.00 | 35.00 | 0.27 | 22.82 |
|  |  |  |  | 2 / y8 | 686.41 | 732.43 | 35.00 | 32.00 | -2.73 | 22.82 |
| Insulin-like growth factor-binding protein 7 | IGFBP7 | Heavy | [2MC]-DNLAIQTR | 2 / y4 | 482.79 | 517.31 | 26.00 | 26.00 | 0.00 | 17.86 |
|  |  |  |  | 2 / y5 | 482.79 | 588.35 | 26.00 | 26.00 | 0.00 | 17.86 |
|  |  |  |  | 2 / y7 | 482.79 | 815.47 | 26.00 | 26.00 | 0.00 | 17.86 |
|  |  | Light | [2Me]-DNLAIQTR | 2 / y4 | 479.77 | 517.31 | 26.00 | 26.00 | 0.13 | 17.86 |
|  |  |  |  | 2 / y5 | 479.77 | 588.35 | 26.00 | 26.00 | 0.00 | 17.86 |
|  |  |  |  | 2 / y7 | 479.77 | 815.47 | 26.00 | 26.00 | 0.00 | 17.86 |
| Inosine-5'-monophosphate dehydrogenase 2 | IMPDH2 | Heavy | [2MC]-VAQGVSGAVQDK[2MC] | 2 / y6 | 613.87 | 651.39 | 32.00 | 35.00 | 3.00 | 14.23 |
|  |  |  |  | 2 / y7 | 613.87 | 738.42 | 32.00 | 29.00 | -3.00 | 14.23 |
|  |  |  |  | 2 / y9 | 613.87 | 894.51 | 32.00 | 29.00 | -3.00 | 14.23 |
|  |  | Light | [2Me]-VAQGVSGAVQDK[2Me] | 2 / y6 | 607.84 | 645.36 | 32.00 | 35.00 | 3.27 | 14.23 |
|  |  |  |  | 2 / y7 | 607.84 | 732.39 | 32.00 | 29.00 | -3.00 | 14.23 |
|  |  |  |  | 2 / y9 | 607.84 | 888.48 | 32.00 | 29.00 | -3.00 | 14.23 |
| Interferon regulatory factor 6 | IRF6 | Heavy | [2MC]-YQEGVDDPDPAK[2MC] | 2 / b6 | 701.36 | 726.35 | 36.00 | 36.00 | 0.00 | 15.63 |
|  |  |  |  | 2 / b7 | 701.36 | 841.38 | 36.00 | 33.00 | -3.00 | 15.63 |
|  |  |  |  | 2 / y5 | 701.36 | 561.35 | 36.00 | 39.00 | 3.00 | 15.63 |
|  |  | Light | [2Me]-YQEGVDDPDPAK[2Me] | 2 / b6 | 695.33 | 720.32 | 36.00 | 36.00 | 0.27 | 15.63 |
|  |  |  |  | 2 / b7 | 695.33 | 835.35 | 36.00 | 33.00 | -2.74 | 15.63 |
|  |  |  |  | 2 / y5 | 695.33 | 555.31 | 36.00 | 39.00 | 3.27 | 15.63 |
| Isoform LAMP-2A of Lysosome-associated membrane glycoprotein 2 | LAMP2 | Heavy | [2MC]-GILTVDELLAIR | 2 / y6 | 673.93 | 714.45 | 35.00 | 41.00 | 6.00 | 45.57 |
|  |  |  |  | 2 / y7 | 673.93 | 829.48 | 35.00 | 32.00 | -3.00 | 45.57 |
|  |  |  |  | 2 / y8 | 673.93 | 928.55 | 35.00 | 32.00 | -3.00 | 45.57 |
|  |  | Light | [2Me]-GILTVDELLAIR | 2 / y6 | 670.91 | 714.45 | 35.00 | 41.00 | 6.00 | 45.57 |
|  |  |  |  | 2 / y7 | 670.91 | 829.48 | 35.00 | 32.00 | -3.00 | 45.57 |
|  |  |  |  | 2 / y8 | 670.91 | 928.55 | 35.00 | 32.00 | -3.00 | 45.57 |
| Isoform 1 of Cytosol aminopeptidase | LAP3 | Heavy | [2MC]-TIQVDNTDAEGR | 2 / y4 | 676.85 | 432.22 | 35.00 | 47.00 | 12.00 | 15.89 |
|  |  |  |  | 2 / y7 | 676.85 | 762.34 | 35.00 | 38.00 | 3.00 | 15.89 |
|  |  |  |  | 2 / y8 | 676.85 | 877.36 | 35.00 | 35.00 | 0.00 | 15.89 |
|  |  | Light | [2Me]-TIQVDNTDAEGR | 2 / y4 | 673.83 | 432.22 | 35.00 | 47.00 | 12.13 | 15.89 |
|  |  |  |  | 2 / y7 | 673.83 | 762.34 | 35.00 | 38.00 | 3.00 | 15.89 |
|  |  |  |  | 2 / y8 | 673.83 | 877.36 | 35.00 | 35.00 | 0.00 | 15.89 |
| Phosphoglycerate mutase | LOC643576 | Heavy | [2MC]-HGESAWNLENR | 2 / b8 | 673.84 | 929.47 | 35.00 | 38.00 | 3.00 | 18.64 |
|  |  |  |  | 2 / y5 | 673.84 | 645.33 | 35.00 | 35.00 | 0.00 | 18.64 |
|  |  |  |  | 2 / y6 | 673.84 | 831.41 | 35.00 | 35.00 | 0.00 | 18.64 |
|  |  | Light | [2Me]-HGESAWNLENR | 2 / b8 | 670.82 | 923.44 | 35.00 | 38.00 | 3.13 | 18.64 |
|  |  |  |  | 2 / y5 | 670.82 | 645.33 | 35.00 | 35.00 | 0.13 | 18.64 |
|  |  |  |  | 2 / y6 | 670.82 | 831.41 | 35.00 | 35.00 | 0.13 | 18.64 |
| Large subunit GTPase 1 homolog | LSG1 | Heavy | [2MC]-LILTPFER | 2 / y4 | 511.83 | 548.28 | 28.00 | 28.00 | 0.00 | 35.42 |
|  |  |  |  | 2 / y6 | 511.83 | 762.41 | 28.00 | 25.00 | -3.00 | 35.42 |
|  |  |  |  | 2 / y7 | 511.83 | 875.5 | 28.00 | 25.00 | -3.00 | 35.42 |
|  |  | Light | [2Me]-LILTPFER | 2 / y4 | 508.81 | 548.28 | 27.00 | 28.00 | 1.00 | 35.42 |
|  |  |  |  | 2 / y6 | 508.81 | 762.41 | 27.00 | 25.00 | -2.00 | 35.42 |
|  |  |  |  | 2 / y7 | 508.81 | 875.5 | 27.00 | 25.00 | -2.87 | 35.42 |
| cDNA FLJ60607, highly similar to Acyl-protein thioesterase 1 | LYPLA1 | Heavy | [2MC]-TLVNPANVTFK[2MC] | 2 / b4 | 636.4 | 462.31 | 33.00 | 30.00 | -3.00 | 29.96 |
|  |  |  |  | 2 / y7 | 636.4 | 810.49 | 33.00 | 33.00 | 0.00 | 29.96 |
|  |  |  |  | 2 / y8 | 636.4 | 924.54 | 33.00 | 30.00 | -3.00 | 29.96 |
|  |  | Light | [2Me]-TLVNPANVTFK[2Me] | 2 / b4 | 630.37 | 456.28 | 33.00 | 30.00 | -2.73 | 29.96 |
|  |  |  |  | 2 / y7 | 630.37 | 804.46 | 33.00 | 33.00 | 0.00 | 29.96 |
|  |  |  |  | 2 / y8 | 630.37 | 918.5 | 33.00 | 30.00 | -3.00 | 29.96 |
| Isoform CSBP2 of Mitogen-activated protein kinase 14 | MAPK14 | Heavy | [2MC]-YIHSADIIHR | 2 / y4 | 629.86 | 538.35 | 33.00 | 42.00 | 9.00 | 18.83 |
|  |  |  |  | 2 / y7 | 629.86 | 811.44 | 33.00 | 33.00 | 0.00 | 18.83 |
|  |  |  |  | 2 / y8 | 629.86 | 948.5 | 33.00 | 30.00 | -3.00 | 18.83 |
|  |  | Light | [2Me]-YIHSADIIHR | 2 / y4 | 626.84 | 538.35 | 33.00 | 42.00 | 9.13 | 18.83 |
|  |  |  |  | 2 / y7 | 626.84 | 811.44 | 33.00 | 33.00 | 0.00 | 18.83 |
|  |  |  |  | 2 / y8 | 626.84 | 948.5 | 33.00 | 30.00 | -2.87 | 18.83 |
| Isoform 1 of Mitochondrial fission process protein 1 | MTFP1 | Heavy | [2MC]-SVDFLLDSSLR | 2 / b5 | 643.36 | 596.35 | 33.00 | 30.00 | -3.00 | 39.28 |
|  |  |  |  | 2 / y4 | 643.36 | 462.27 | 33.00 | 42.00 | 9.00 | 39.28 |
|  |  |  |  | 2 / y8 | 643.36 | 950.53 | 33.00 | 33.00 | 0.00 | 39.28 |
|  |  | Light | [2Me]-SVDFLLDSSLR | 2 / b5 | 640.35 | 590.32 | 33.00 | 30.00 | -2.87 | 39.28 |
|  |  |  |  | 2 / y4 | 640.35 | 462.27 | 33.00 | 42.00 | 9.13 | 39.28 |
|  |  |  |  | 2 / y8 | 640.35 | 950.53 | 33.00 | 33.00 | 0.00 | 39.28 |
| Protein NDRG1 | NDRG1 | Heavy | [2MC]-TASGSSVTSLDGTR | 2 / y7 | 686.86 | 749.38 | 35.00 | 35.00 | 0.00 | 18.31 |
|  |  |  |  | 2 / y8 | 686.86 | 848.45 | 35.00 | 35.00 | 0.00 | 18.31 |
|  |  |  |  | 2 / y9 | 686.86 | 935.48 | 35.00 | 38.00 | 3.00 | 18.31 |
|  |  | Light | [2Me]-TASGSSVTSLDGTR | 2 / y7 | 683.84 | 749.38 | 35.00 | 35.00 | 0.13 | 18.31 |
|  |  |  |  | 2 / y8 | 683.84 | 848.45 | 35.00 | 35.00 | 0.00 | 18.31 |
|  |  |  |  | 2 / y9 | 683.84 | 935.48 | 35.00 | 38.00 | 3.00 | 18.31 |
| Omega-amidase NIT2 | NIT2 | Heavy | [2MC]-AVDNQVYVATASPAR | 2 / b5 | 798.43 | 562.3 | 40.00 | 46.00 | 6.00 | 20.12 |
|  |  |  |  | 2 / y8 | 798.43 | 772.43 | 40.00 | 37.00 | -3.00 | 20.12 |
|  |  |  |  | 2 / y9 | 798.43 | 935.49 | 40.00 | 37.00 | -3.00 | 20.12 |
|  |  | Light | [2Me]-AVDNQVYVATASPAR | 2 / b5 | 795.42 | 556.27 | 40.00 | 46.00 | 6.13 | 20.12 |
|  |  |  |  | 2 / y8 | 795.42 | 772.43 | 40.00 | 37.00 | -2.87 | 20.12 |
|  |  |  |  | 2 / y9 | 795.42 | 935.49 | 40.00 | 37.00 | -2.87 | 20.12 |
| Cancer-related nucleoside-triphosphatase | NTPCR | Heavy | [2MC]-IGFDVVTLSGTR | 2 / b4 | 649.88 | 467.27 | 34.00 | 34.00 | 0.00 | 36.67 |
|  |  |  |  | 2 / y7 | 649.88 | 733.42 | 34.00 | 34.00 | 0.00 | 36.67 |
|  |  |  |  | 2 / y8 | 649.88 | 832.49 | 34.00 | 40.00 | 6.00 | 36.67 |
|  |  | Light | [2Me]-IGFDVVTLSGTR | 2 / b4 | 646.86 | 461.24 | 33.00 | 34.00 | 0.13 | 36.67 |
|  |  |  |  | 2 / y7 | 646.86 | 733.42 | 33.00 | 34.00 | 0.13 | 36.67 |
|  |  |  |  | 2 / y8 | 646.86 | 832.49 | 33.00 | 40.00 | 7.00 | 36.67 |
| Nuclear migration protein nudC | NUDC | Heavy | [2MC]-ELTDEEAER | 2 / y5 | 563.28 | 633.28 | 30.00 | 36.00 | 6.00 | 21.20 |
|  |  |  |  | 2 / y7 | 563.28 | 849.36 | 30.00 | 27.00 | -3.00 | 21.20 |
|  |  |  |  | 2 / y8 | 563.28 | 962.44 | 30.00 | 27.00 | -3.00 | 21.20 |
|  |  | Light | [2Me]-ELTDEEAER | 2 / y5 | 560.26 | 633.28 | 30.00 | 36.00 | 6.00 | 21.20 |
|  |  |  |  | 2 / y7 | 560.26 | 849.36 | 30.00 | 27.00 | -3.00 | 21.20 |
|  |  |  |  | 2 / y8 | 560.26 | 962.44 | 30.00 | 27.00 | -3.00 | 21.20 |
| ADP-sugar pyrophosphatase | NUDT5 | Heavy | [2MC]-QYIISEELISEGK[2MC] | 2 / b3 | 788.96 | 439.28 | 40.00 | 43.00 | 3.00 | 34.50 |
|  |  |  |  | 2 / b4 | 788.96 | 552.36 | 40.00 | 37.00 | -3.00 | 34.50 |
|  |  |  |  | 2 / y4 | 788.96 | 454.27 | 40.00 | 49.00 | 9.00 | 34.50 |
|  |  | Light | [2Me]-QYIISEELISEGK[2Me] | 2 / b3 | 782.93 | 433.24 | 39.00 | 43.00 | 3.27 | 34.50 |
|  |  |  |  | 2 / b4 | 782.93 | 546.33 | 39.00 | 37.00 | -2.74 | 34.50 |
|  |  |  |  | 2 / y4 | 782.93 | 448.24 | 39.00 | 49.00 | 9.27 | 34.50 |
| Nuclear mitotic apparatus protein 1 | NUMA1 | Heavy | [2MC]-GEVLGDVLQLETLK[2MC] | 2 / b6 | 791.49 | 605.34 | 40.00 | 43.00 | 3.00 | 43.05 |
|  |  |  |  | 2 / y7 | 791.49 | 878.58 | 40.00 | 37.00 | -3.00 | 43.05 |
|  |  |  |  | 2 / y8 | 791.49 | 977.65 | 40.00 | 40.00 | 0.00 | 43.05 |
|  |  | Light | [2Me]-GEVLGDVLQLETLK[2Me] | 2 / b6 | 785.46 | 599.3 | 40.00 | 43.00 | 3.27 | 43.05 |
|  |  |  |  | 2 / y7 | 785.46 | 872.55 | 40.00 | 37.00 | -2.73 | 43.05 |
|  |  |  |  | 2 / y8 | 785.46 | 971.61 | 40.00 | 40.00 | 0.00 | 43.05 |
| Bifunctional 3'-phosphoadenosine 5'-phosphosulfate synthase 1 | PAPSS1 | Heavy | [2MC]-TDAETLPALK[2MC] | 2 / y4 | 563.85 | 462.35 | 30.00 | 39.00 | 9.00 | 26.05 |
|  |  |  |  | 2 / y6 | 563.85 | 676.48 | 30.00 | 30.00 | 0.00 | 26.05 |
|  |  |  |  | 2 / y8 | 563.85 | 876.56 | 30.00 | 30.00 | 0.00 | 26.05 |
|  |  | Light | [2Me]-TDAETLPALK[2Me] | 2 / y4 | 557.82 | 456.32 | 30.00 | 39.00 | 9.27 | 26.05 |
|  |  |  |  | 2 / y6 | 557.82 | 670.45 | 30.00 | 30.00 | 0.00 | 26.05 |
|  |  |  |  | 2 / y8 | 557.82 | 870.53 | 30.00 | 30.00 | 0.00 | 26.05 |
| Prefoldin subunit 1 | PFDN1 | Heavy | [2MC]-AFTELQAK[2MC] | 2 / y4 | 488.31 | 493.36 | 26.00 | 32.00 | 6.00 | 29.20 |
|  |  |  |  | 2 / y6 | 488.31 | 723.45 | 26.00 | 23.00 | -3.00 | 29.20 |
|  |  |  |  | 2 / y7 | 488.31 | 870.51 | 26.00 | 23.00 | -3.00 | 29.20 |
|  |  | Light | [2Me]-AFTELQAK[2Me] | 2 / y4 | 482.28 | 487.32 | 26.00 | 32.00 | 6.00 | 29.20 |
|  |  |  |  | 2 / y6 | 482.28 | 717.41 | 26.00 | 23.00 | -3.00 | 29.20 |
|  |  |  |  | 2 / y7 | 482.28 | 864.48 | 26.00 | 23.00 | -3.00 | 29.20 |
| Prefoldin subunit 4 | PFDN4 | Heavy | [2MC]-AAAEDVNVTFEDQQK[2MC] | 2 / b5 | 866.95 | 492.25 | 43.00 | 49.00 | 6.00 | 22.78 |
|  |  |  |  | 2 / y6 | 866.95 | 828.43 | 43.00 | 40.00 | -3.00 | 22.78 |
|  |  |  |  | 2 / y7 | 866.95 | 929.48 | 43.00 | 43.00 | 0.00 | 22.78 |
|  |  | Light | [2Me]-AAAEDVNVTFEDQQK[2Me] | 2 / b5 | 860.92 | 486.22 | 43.00 | 49.00 | 6.27 | 22.78 |
|  |  |  |  | 2 / y6 | 860.92 | 822.4 | 43.00 | 40.00 | -2.74 | 22.78 |
|  |  |  |  | 2 / y7 | 860.92 | 923.45 | 43.00 | 43.00 | 0.27 | 22.78 |
| Phosphoglycerate kinase 1 | PGK1 | Heavy | [2MC]-ITLPVDFVTADK[2MC] | 2 / y4 | 693.93 | 468.29 | 36.00 | 48.00 | 12.00 | 48.10 |
|  |  |  |  | 2 / y6 | 693.93 | 714.42 | 36.00 | 42.00 | 6.00 | 48.10 |
|  |  |  |  | 2 / y7 | 693.93 | 829.45 | 36.00 | 39.00 | 3.00 | 48.10 |
|  |  | Light | [2Me]-ITLPVDFVTADK[2Me] | 2 / y4 | 687.9 | 462.26 | 35.00 | 48.00 | 12.27 | 48.10 |
|  |  |  |  | 2 / y6 | 687.9 | 708.39 | 35.00 | 42.00 | 7.00 | 48.10 |
|  |  |  |  | 2 / y7 | 687.9 | 823.42 | 35.00 | 39.00 | 4.00 | 48.10 |
| Isoform 1 of Phosphatidylinositol transfer protein beta isoform | PITPNB | Heavy | [2MC]-NETGGGEGIEVLK[2MC] | 2 / y10 | 685.89 | 992.58 | 35.00 | 35.00 | 0.00 | 32.40 |
|  |  |  |  | 2 / y4 | 685.89 | 522.37 | 35.00 | 41.00 | 6.00 | 32.40 |
|  |  |  |  | 2 / y6 | 685.89 | 692.48 | 35.00 | 41.00 | 6.00 | 32.40 |
|  |  | Light | [2Me]-NETGGGEGIEVLK[2Me] | 2 / y10 | 679.86 | 986.55 | 35.00 | 35.00 | 0.09 | 32.40 |
|  |  |  |  | 2 / y4 | 679.86 | 516.34 | 35.00 | 41.00 | 6.27 | 32.40 |
|  |  |  |  | 2 / y6 | 679.86 | 686.44 | 35.00 | 41.00 | 6.00 | 32.40 |
| Perilipin-2 | PLIN2 | Heavy | [2MC]-LGSLSTK[2MC] | 2 / y3 | 387.27 | 369.26 | 22.00 | 31.00 | 8.64 | 19.78 |
|  |  |  |  | 2 / y5 | 387.27 | 569.37 | 22.00 | 22.00 | 0.00 | 19.78 |
|  |  |  |  | 2 / y6 | 387.27 | 626.39 | 22.00 | 22.00 | 0.00 | 19.78 |
|  |  | Light | [2Me]-LGSLSTK[2Me] | 2 / y3 | 381.24 | 363.22 | 22.00 | 31.00 | 9.27 | 19.78 |
|  |  |  |  | 2 / y5 | 381.24 | 563.34 | 22.00 | 22.00 | 0.00 | 19.78 |
|  |  |  |  | 2 / y6 | 381.24 | 620.36 | 22.00 | 22.00 | 0.00 | 19.78 |
| Plastin-3 | PLS3 | Heavy | [2MC]-NEALAALLR | 2 / y4 | 502.82 | 472.32 | 27.00 | 30.00 | 3.00 | 43.50 |
|  |  |  |  | 2 / y5 | 502.82 | 543.36 | 27.00 | 30.00 | 3.00 | 43.50 |
|  |  |  |  | 2 / y7 | 502.82 | 727.48 | 27.00 | 24.00 | -3.00 | 43.50 |
|  |  | Light | [2Me]-NEALAALLR | 2 / y4 | 499.8 | 472.32 | 27.00 | 30.00 | 3.13 | 43.50 |
|  |  |  |  | 2 / y5 | 499.8 | 543.36 | 27.00 | 30.00 | 3.13 | 43.50 |
|  |  |  |  | 2 / y7 | 499.8 | 727.48 | 27.00 | 24.00 | -3.00 | 43.50 |
| Prolyl endopeptidase | PREP | Heavy | [2MC]-NILQLHDLTTGALLK[2MC] | 2 / y7 | 859.55 | 737.5 | 43.00 | 46.00 | 3.00 | 40.65 |
|  |  |  |  | 2 / y8 | 859.55 | 850.58 | 43.00 | 43.00 | 0.00 | 40.65 |
|  |  |  |  | 2 / y9 | 859.55 | 965.61 | 43.00 | 46.00 | 3.00 | 40.65 |
|  |  | Light | [2Me]-NILQLHDLTTGALLK[2Me] | 2 / y7 | 853.51 | 731.47 | 43.00 | 46.00 | 3.27 | 40.65 |
|  |  |  |  | 2 / y8 | 853.51 | 844.55 | 43.00 | 43.00 | 0.27 | 40.65 |
|  |  |  |  | 2 / y9 | 853.51 | 959.58 | 43.00 | 46.00 | 3.27 | 40.65 |
| U4/U6 small nuclear ribonucleoprotein Prp3 | PRPF3 | Heavy | [2MC]-LQAEISQAAR | 2 / y6 | 560.83 | 645.37 | 30.00 | 36.00 | 6.00 | 18.92 |
|  |  |  |  | 2 / y8 | 560.83 | 845.45 | 30.00 | 30.00 | 0.00 | 18.92 |
|  |  |  |  | 2 / y9 | 560.83 | 973.51 | 30.00 | 30.00 | 0.00 | 18.92 |
|  |  | Light | [2Me]-LQAEISQAAR | 2 / y6 | 557.81 | 645.37 | 30.00 | 36.00 | 6.00 | 18.92 |
|  |  |  |  | 2 / y8 | 557.81 | 845.45 | 30.00 | 30.00 | 0.00 | 18.92 |
|  |  |  |  | 2 / y9 | 557.81 | 973.51 | 30.00 | 30.00 | 0.13 | 18.92 |
| Ras-related protein Rab-11B | RAB11B | Heavy | [2MC]-STIGVEFATR | 2 / y7 | 557.82 | 779.4 | 30.00 | 30.00 | 0.00 | 33.80 |
|  |  |  |  | 2 / y8 | 557.82 | 892.49 | 30.00 | 27.00 | -3.00 | 33.80 |
|  |  |  |  | 2 / y9 | 557.82 | 993.54 | 30.00 | 30.00 | 0.00 | 33.80 |
|  |  | Light | [2Me]-STIGVEFATR | 2 / y7 | 554.8 | 779.4 | 29.00 | 30.00 | 1.00 | 33.80 |
|  |  |  |  | 2 / y8 | 554.8 | 892.49 | 29.00 | 27.00 | -2.00 | 33.80 |
|  |  |  |  | 2 / y9 | 554.8 | 993.54 | 29.00 | 30.00 | 1.00 | 33.80 |
| Isoform 1 of Rab3 GTPase-activating protein non-catalytic subunit | RAB3GAP2 | Heavy | [2MC]-VEPATPLAVR | 2 / y5 | 543.84 | 555.36 | 29.00 | 32.00 | 3.00 | 24.26 |
|  |  |  |  | 2 / y8 | 543.84 | 824.5 | 29.00 | 26.00 | -3.00 | 24.26 |
|  |  |  |  | 2 / y9 | 543.84 | 953.54 | 29.00 | 26.00 | -3.00 | 24.26 |
|  |  | Light | [2Me]-VEPATPLAVR | 2 / y5 | 540.82 | 555.36 | 29.00 | 32.00 | 3.00 | 24.26 |
|  |  |  |  | 2 / y8 | 540.82 | 824.5 | 29.00 | 26.00 | -3.00 | 24.26 |
|  |  |  |  | 2 / y9 | 540.82 | 953.54 | 29.00 | 26.00 | -2.87 | 24.26 |
| Isoform 1 of Ribonuclease T2 | RNASET2 | Heavy | [2MC]-ELDLNSVLLK[2MC] | 2 / y6 | 606.4 | 707.49 | 32.00 | 35.00 | 3.00 | 39.35 |
|  |  |  |  | 2 / y7 | 606.4 | 820.57 | 32.00 | 32.00 | 0.00 | 39.35 |
|  |  |  |  | 2 / y8 | 606.4 | 935.6 | 32.00 | 32.00 | 0.00 | 39.35 |
|  |  | Light | [2Me]-ELDLNSVLLK[2Me] | 2 / y6 | 600.37 | 701.46 | 31.00 | 35.00 | 3.27 | 39.35 |
|  |  |  |  | 2 / y7 | 600.37 | 814.54 | 31.00 | 32.00 | 1.00 | 39.35 |
|  |  |  |  | 2 / y8 | 600.37 | 929.57 | 31.00 | 32.00 | 1.00 | 39.35 |
| 60S ribosomal protein L7-like 1 | RPL7L1 | Heavy | [2MC]-TIPLTDNTVIEEHLGK[2MC] | 2 / y4 | 924.54 | 488.34 | 46.00 | 58.00 | 12.00 | 33.71 |
|  |  |  |  | 2 / y7 | 924.54 | 859.51 | 46.00 | 49.00 | 3.00 | 33.71 |
|  |  |  |  | 2 / y8 | 924.54 | 958.58 | 46.00 | 49.00 | 3.00 | 33.71 |
|  |  | Light | [2Me]-TIPLTDNTVIEEHLGK[2Me] | 2 / y4 | 918.51 | 482.31 | 45.00 | 58.00 | 12.27 | 33.71 |
|  |  |  |  | 2 / y7 | 918.51 | 853.48 | 45.00 | 49.00 | 3.27 | 33.71 |
|  |  |  |  | 2 / y8 | 918.51 | 952.55 | 45.00 | 49.00 | 3.27 | 33.71 |
| Isoform 1 of RuvB-like 1 | RUVBL1 | Heavy | [2MC]-AVLLAGPPGTGK[2MC] | 2 / y6 | 574.89 | 590.37 | 30.00 | 27.00 | -3.00 | 32.80 |
|  |  |  |  | 2 / y7 | 574.89 | 647.39 | 30.00 | 27.00 | -3.00 | 32.80 |
|  |  |  |  | 2 / y8 | 574.89 | 718.43 | 30.00 | 27.00 | -3.00 | 32.80 |
|  |  | Light | [2Me]-AVLLAGPPGTGK[2Me] | 2 / y6 | 568.86 | 584.34 | 30.00 | 27.00 | -3.00 | 32.80 |
|  |  |  |  | 2 / y7 | 568.86 | 641.36 | 30.00 | 27.00 | -3.00 | 32.80 |
|  |  |  |  | 2 / y8 | 568.86 | 712.4 | 30.00 | 27.00 | -3.00 | 32.80 |
| SAP domain-containing ribonucleoprotein | SARNP | Heavy | [2MC]-ITSEIPQTER | 2 / y5 | 604.34 | 630.32 | 32.00 | 32.00 | 0.00 | 17.83 |
|  |  |  |  | 2 / y6 | 604.34 | 743.4 | 32.00 | 38.00 | 6.00 | 17.83 |
|  |  |  |  | 2 / y8 | 604.34 | 959.48 | 32.00 | 29.00 | -3.00 | 17.83 |
|  |  | Light | [2Me]-ITSEIPQTER | 2 / y5 | 601.32 | 630.32 | 31.00 | 32.00 | 1.00 | 17.83 |
|  |  |  |  | 2 / y6 | 601.32 | 743.4 | 31.00 | 38.00 | 7.00 | 17.83 |
|  |  |  |  | 2 / y8 | 601.32 | 959.48 | 31.00 | 29.00 | -2.00 | 17.83 |
| cDNA FLJ35730 fis, clone TESTI2003131, highly similar to ALPHA-1-ANTICHYMOTRYPSIN | SERPINA3 | Heavy | [2MC]-ITLLSALVETR | 2 / b4 | 625.4 | 475.37 | 33.00 | 30.00 | -3.00 | 43.74 |
|  |  |  |  | 2 / y7 | 625.4 | 775.43 | 33.00 | 30.00 | -3.00 | 43.74 |
|  |  |  |  | 2 / y8 | 625.4 | 888.51 | 33.00 | 30.00 | -3.00 | 43.74 |
|  |  | Light | [2Me]-ITLLSALVETR | 2 / b4 | 622.38 | 469.34 | 32.00 | 30.00 | -2.87 | 43.74 |
|  |  |  |  | 2 / y7 | 622.38 | 775.43 | 32.00 | 30.00 | -2.00 | 43.74 |
|  |  |  |  | 2 / y8 | 622.38 | 888.51 | 32.00 | 30.00 | -2.00 | 43.74 |
| maspin | SERPINB5_maspin | Heavy | [2MC]-NIIFFGK[2MC] | 2 / y4 | 453.81 | 532.33 | 25.00 | 25.00 | 0.00 | 34.33 |
|  |  |  |  | 2 / y5 | 453.81 | 645.42 | 25.00 | 22.00 | -3.00 | 34.33 |
|  |  |  |  | 2 / y6 | 453.81 | 758.5 | 25.00 | 25.00 | 0.00 | 34.33 |
|  |  | Light | [2Me]-NIIFFGK[2Me] | 2 / y4 | 447.78 | 526.3 | 25.00 | 25.00 | 0.00 | 34.33 |
|  |  |  |  | 2 / y5 | 447.78 | 639.39 | 25.00 | 22.00 | -3.00 | 34.33 |
|  |  |  |  | 2 / y6 | 447.78 | 752.47 | 25.00 | 25.00 | 0.00 | 34.33 |
| Isoform 2 of Serpin B5 | SERPINB5-Isoform 2 | Heavy | [2MC]-ELETVDFK[2MC] | 2 / b3 | 524.81 | 406.24 | 28.00 | 28.00 | 0.00 | 33.40 |
|  |  |  |  | 2 / b6 | 524.81 | 721.38 | 28.00 | 22.00 | -6.00 | 33.40 |
|  |  |  |  | 2 / y7 | 524.81 | 885.51 | 28.00 | 25.00 | -3.00 | 33.40 |
|  |  | Light | [2Me]-ELETVDFK[2Me] | 2 / b3 | 518.78 | 400.21 | 28.00 | 28.00 | 0.27 | 33.40 |
|  |  |  |  | 2 / b6 | 518.78 | 715.35 | 28.00 | 22.00 | -6.00 | 33.40 |
|  |  |  |  | 2 / y7 | 518.78 | 879.48 | 28.00 | 25.00 | -3.00 | 33.40 |
| Serpin H1 | SERPINH1 | Heavy | [2MC]-AVAISLPK[2MC] | 2 / y4 | 433.82 | 478.34 | 24.00 | 24.00 | 0.00 | 34.60 |
|  |  |  |  | 2 / y6 | 433.82 | 662.47 | 24.00 | 21.00 | -3.00 | 34.60 |
|  |  |  |  | 2 / y7 | 433.82 | 761.53 | 24.00 | 21.00 | -3.00 | 34.60 |
|  |  | Light | [2Me]-AVAISLPK[2Me] | 2 / y4 | 427.79 | 472.31 | 24.00 | 24.00 | 0.18 | 34.60 |
|  |  |  |  | 2 / y6 | 427.79 | 656.43 | 24.00 | 21.00 | -3.00 | 34.60 |
|  |  |  |  | 2 / y7 | 427.79 | 755.5 | 24.00 | 21.00 | -3.00 | 34.60 |
| Isoform 1 of 14-3-3 protein sigma | SFN | Heavy | [2MC]-YLAEVATGDDK[2MC] | 2 / b4 | 625.35 | 511.3 | 33.00 | 33.00 | 0.00 | 17.90 |
|  |  |  |  | 2 / b5 | 625.35 | 610.37 | 33.00 | 30.00 | -3.00 | 17.90 |
|  |  |  |  | 2 / y9 | 625.35 | 939.48 | 33.00 | 30.00 | -3.00 | 17.90 |
|  |  | Light | [2Me]-YLAEVATGDDK[2Me] | 2 / b4 | 619.32 | 505.27 | 32.00 | 33.00 | 0.27 | 17.90 |
|  |  |  |  | 2 / b5 | 619.32 | 604.33 | 32.00 | 30.00 | -2.74 | 17.90 |
|  |  |  |  | 2 / y9 | 619.32 | 933.45 | 32.00 | 30.00 | -2.00 | 17.90 |
| Isoform 2 of 4F2 cell-surface antigen heavy chain | SLC3A2 | Heavy | [2MC]-EDFDSLLQSAK[2MC] | 2 / b4 | 660.87 | 541.24 | 34.00 | 34.00 | 0.00 | 32.85 |
|  |  |  |  | 2 / y5 | 660.87 | 580.39 | 34.00 | 34.00 | 0.00 | 32.85 |
|  |  |  |  | 2 / y7 | 660.87 | 780.5 | 34.00 | 34.00 | 0.00 | 32.85 |
|  |  | Light | [2Me]-EDFDSLLQSAK[2Me] | 2 / b4 | 654.84 | 535.2 | 34.00 | 34.00 | 0.27 | 32.85 |
|  |  |  |  | 2 / y5 | 654.84 | 574.36 | 34.00 | 34.00 | 0.27 | 32.85 |
|  |  |  |  | 2 / y7 | 654.84 | 774.47 | 34.00 | 34.00 | 0.00 | 32.85 |
| Isoform 1 of Spermine synthase | SMS | Heavy | [2MC]-ADGETILK[2MC] | 2 / y4 | 457.8 | 508.39 | 25.00 | 28.00 | 3.00 | 17.78 |
|  |  |  |  | 2 / y6 | 457.8 | 694.46 | 25.00 | 25.00 | 0.00 | 17.78 |
|  |  |  |  | 2 / y7 | 457.8 | 809.48 | 25.00 | 22.00 | -3.00 | 17.78 |
|  |  | Light | [2Me]-ADGETILK[2Me] | 2 / y4 | 451.76 | 502.36 | 25.00 | 28.00 | 3.00 | 17.78 |
|  |  |  |  | 2 / y6 | 451.76 | 688.42 | 25.00 | 25.00 | 0.00 | 17.78 |
|  |  |  |  | 2 / y7 | 451.76 | 803.45 | 25.00 | 22.00 | -3.00 | 17.78 |
| Isoform 2 of Proto-oncogene tyrosine-protein kinase Src | SRC | Heavy | [2MC]-WTAPEAALYGR | 2 / y4 | 634.85 | 508.29 | 33.00 | 33.00 | 0.00 | 36.50 |
|  |  |  |  | 2 / y5 | 634.85 | 579.32 | 33.00 | 33.00 | 0.00 | 36.50 |
|  |  |  |  | 2 / y6 | 634.85 | 650.36 | 33.00 | 39.00 | 6.00 | 36.50 |
|  |  | Light | [2Me]-WTAPEAALYGR | 2 / y4 | 631.83 | 508.29 | 33.00 | 33.00 | 0.13 | 36.50 |
|  |  |  |  | 2 / y5 | 631.83 | 579.32 | 33.00 | 33.00 | 0.13 | 36.50 |
|  |  |  |  | 2 / y6 | 631.83 | 650.36 | 33.00 | 39.00 | 6.00 | 36.50 |
| Serine/threonine-protein kinase 25 | STK25 | Heavy | [2MC]-ADIWSLGITAIELAK[2MC] | 2 / b4 | 835.01 | 520.3 | 42.00 | 39.00 | -3.00 | 50.92 |
|  |  |  |  | 2 / y7 | 835.01 | 779.51 | 42.00 | 39.00 | -3.00 | 50.92 |
|  |  |  |  | 2 / y9 | 835.01 | 949.61 | 42.00 | 39.00 | -3.00 | 50.92 |
|  |  | Light | [2Me]-ADIWSLGITAIELAK[2Me] | 2 / b4 | 828.98 | 514.27 | 41.00 | 39.00 | -2.73 | 50.92 |
|  |  |  |  | 2 / y7 | 828.98 | 773.48 | 41.00 | 39.00 | -2.73 | 50.92 |
|  |  |  |  | 2 / y9 | 828.98 | 943.58 | 41.00 | 39.00 | -2.00 | 50.92 |
| Tax1-binding protein 3 | TAX1BP3 | Heavy | [2MC]-VSEGGPAEIAGLQIGDK[2MC] | 2 / b9 | 854.99 | 874.47 | 43.00 | 40.00 | -3.00 | 30.61 |
|  |  |  |  | 2 / y8 | 854.99 | 835.51 | 43.00 | 46.00 | 3.00 | 30.61 |
|  |  |  |  | 2 / y9 | 854.99 | 948.59 | 43.00 | 46.00 | 3.00 | 30.61 |
|  |  | Light | [2Me]-VSEGGPAEIAGLQIGDK[2Me] | 2 / b9 | 848.96 | 868.44 | 42.00 | 40.00 | -2.73 | 30.61 |
|  |  |  |  | 2 / y8 | 848.96 | 829.48 | 42.00 | 46.00 | 3.27 | 30.61 |
|  |  |  |  | 2 / y9 | 848.96 | 942.56 | 42.00 | 46.00 | 4.00 | 30.61 |
| Protein TFG | TFG | Heavy | [2MC]-LLSNDEVTIK[2MC] | 2 / y5 | 600.38 | 623.42 | 31.00 | 37.00 | 6.00 | 25.07 |
|  |  |  |  | 2 / y6 | 600.38 | 738.45 | 31.00 | 34.00 | 3.00 | 25.07 |
|  |  |  |  | 2 / y8 | 600.38 | 939.52 | 31.00 | 31.00 | 0.00 | 25.07 |
|  |  | Light | [2Me]-LLSNDEVTIK[2Me] | 2 / y5 | 594.35 | 617.39 | 31.00 | 37.00 | 6.00 | 25.07 |
|  |  |  |  | 2 / y6 | 594.35 | 732.41 | 31.00 | 34.00 | 3.00 | 25.07 |
|  |  |  |  | 2 / y8 | 594.35 | 933.49 | 31.00 | 31.00 | 0.27 | 25.07 |
| Transferrin receptor protein 1 | TFRC | Heavy | [2MC]-DSAQNSVIIVDK[2MC] | 2 / y4 | 678.9 | 508.36 | 35.00 | 35.00 | 0.00 | 21.82 |
|  |  |  |  | 2 / y5 | 678.9 | 621.44 | 35.00 | 35.00 | 0.00 | 21.82 |
|  |  |  |  | 2 / y7 | 678.9 | 807.54 | 35.00 | 32.00 | -3.00 | 21.82 |
|  |  | Light | [2Me]-DSAQNSVIIVDK[2Me] | 2 / y4 | 672.87 | 502.32 | 35.00 | 35.00 | 0.27 | 21.82 |
|  |  |  |  | 2 / y5 | 672.87 | 615.41 | 35.00 | 35.00 | 0.27 | 21.82 |
|  |  |  |  | 2 / y7 | 672.87 | 801.51 | 35.00 | 32.00 | -3.00 | 21.82 |
| Thy-1 membrane glycoprotein | THY1 | Heavy | [2MC]-HVLFGTVGVPEHTYR | 2 / y4 | 873.48 | 576.29 | 43.00 | 46.00 | 3.00 | 27.77 |
|  |  |  |  | 2 / y6 | 873.48 | 802.38 | 43.00 | 46.00 | 3.00 | 27.77 |
|  |  |  |  | 2 / y8 | 873.48 | 958.47 | 43.00 | 43.00 | 0.00 | 27.77 |
|  |  | Light | [2Me]-HVLFGTVGVPEHTYR | 2 / y4 | 870.46 | 576.29 | 43.00 | 46.00 | 3.13 | 27.77 |
|  |  |  |  | 2 / y6 | 870.46 | 802.38 | 43.00 | 46.00 | 3.13 | 27.77 |
|  |  |  |  | 2 / y8 | 870.46 | 958.47 | 43.00 | 43.00 | 0.00 | 27.77 |
| Mitochondrial import inner membrane translocase subunit Tim13 | TIMM13 | Heavy | [2MC]-VQIAVANAQELLQR | 2 / b4 | 793.98 | 446.32 | 40.00 | 43.00 | 3.00 | 43.50 |
|  |  |  |  | 2 / b5 | 793.98 | 545.39 | 40.00 | 37.00 | -3.00 | 43.50 |
|  |  |  |  | 2 / y8 | 793.98 | 971.53 | 40.00 | 34.00 | -6.00 | 43.50 |
|  |  | Light | [2Me]-VQIAVANAQELLQR | 2 / b4 | 790.96 | 440.29 | 40.00 | 43.00 | 3.13 | 43.50 |
|  |  |  |  | 2 / b5 | 790.96 | 539.36 | 40.00 | 37.00 | -2.87 | 43.50 |
|  |  |  |  | 2 / y8 | 790.96 | 971.53 | 40.00 | 34.00 | -6.00 | 43.50 |
| Mitochondrial import inner membrane translocase subunit Tim8 A | TIMM8A | Heavy | [2MC]-FIDTSQFILNR | 2 / b7 | 694.39 | 873.46 | 36.00 | 36.00 | 0.00 | 35.36 |
|  |  |  |  | 2 / y7 | 694.39 | 877.49 | 36.00 | 36.00 | 0.00 | 35.36 |
|  |  |  |  | 2 / y8 | 694.39 | 978.54 | 36.00 | 39.00 | 3.00 | 35.36 |
|  |  | Light | [2Me]-FIDTSQFILNR | 2 / b7 | 691.38 | 867.42 | 35.00 | 36.00 | 0.13 | 35.36 |
|  |  |  |  | 2 / y7 | 691.38 | 877.49 | 35.00 | 36.00 | 1.00 | 35.36 |
|  |  |  |  | 2 / y8 | 691.38 | 978.54 | 35.00 | 39.00 | 4.00 | 35.36 |
| Isoform 1 of Tubulointerstitial nephritis antigen-like | TINAGL1 | Heavy | [2MC]-ITGWGEETLPDGR | 2 / y4 | 732.88 | 444.22 | 37.00 | 34.00 | -3.00 | 29.29 |
|  |  |  |  | 2 / y7 | 732.88 | 787.39 | 37.00 | 40.00 | 3.00 | 29.29 |
|  |  |  |  | 2 / y9 | 732.88 | 973.46 | 37.00 | 37.00 | 0.00 | 29.29 |
|  |  | Light | [2Me]-ITGWGEETLPDGR | 2 / y4 | 729.86 | 444.22 | 37.00 | 34.00 | -2.87 | 29.29 |
|  |  |  |  | 2 / y7 | 729.86 | 787.39 | 37.00 | 40.00 | 3.00 | 29.29 |
|  |  |  |  | 2 / y9 | 729.86 | 973.46 | 37.00 | 37.00 | 0.13 | 29.29 |
| Transmembrane protein 43 | TMEM43 | Heavy | [2MC]-EYTEDGQVK[2MC] | 2 / y4 | 568.81 | 465.32 | 30.00 | 36.00 | 6.00 | 21.00 |
|  |  |  |  | 2 / y7 | 568.81 | 810.44 | 30.00 | 30.00 | 0.00 | 21.00 |
|  |  |  |  | 2 / y8 | 568.81 | 973.5 | 30.00 | 27.00 | -3.00 | 21.00 |
|  |  | Light | [2Me]-EYTEDGQVK[2Me] | 2 / y4 | 562.78 | 459.29 | 30.00 | 36.00 | 6.27 | 21.00 |
|  |  |  |  | 2 / y7 | 562.78 | 804.41 | 30.00 | 30.00 | 0.00 | 21.00 |
|  |  |  |  | 2 / y8 | 562.78 | 967.47 | 30.00 | 27.00 | -3.00 | 21.00 |
| Transmembrane protein 97 | TMEM97 | Heavy | [2MC]-ELYPVEFR | 2 / y5 | 543.81 | 647.35 | 29.00 | 26.00 | -3.00 | 29.02 |
|  |  |  |  | 2 / y6 | 543.81 | 810.41 | 29.00 | 23.00 | -6.00 | 29.02 |
|  |  |  |  | 2 / y7 | 543.81 | 923.5 | 29.00 | 23.00 | -6.00 | 29.02 |
|  |  | Light | [2Me]-ELYPVEFR | 2 / y5 | 540.79 | 647.35 | 29.00 | 26.00 | -3.00 | 29.02 |
|  |  |  |  | 2 / y6 | 540.79 | 810.41 | 29.00 | 23.00 | -6.00 | 29.02 |
|  |  |  |  | 2 / y7 | 540.79 | 923.5 | 29.00 | 23.00 | -5.87 | 29.02 |
| triosephosphate isomerase isoform 2 | TPI1 | Heavy | [2MC]-SNVSDAVAQSTR | 2 / y5 | 634.84 | 562.29 | 33.00 | 33.00 | 0.00 | 15.40 |
|  |  |  |  | 2 / y7 | 634.84 | 732.4 | 33.00 | 36.00 | 3.00 | 15.40 |
|  |  |  |  | 2 / y9 | 634.84 | 934.46 | 33.00 | 33.00 | 0.00 | 15.40 |
|  |  | Light | [2Me]-SNVSDAVAQSTR | 2 / y5 | 631.82 | 562.29 | 33.00 | 33.00 | 0.13 | 15.40 |
|  |  |  |  | 2 / y7 | 631.82 | 732.4 | 33.00 | 36.00 | 3.00 | 15.40 |
|  |  |  |  | 2 / y9 | 631.82 | 934.46 | 33.00 | 33.00 | 0.00 | 15.40 |
| Isoform 2 of Cdc42-interacting protein 4 | TRIP10 | Heavy | [2MC]-ELVAENLSVR | 2 / y5 | 582.35 | 588.35 | 31.00 | 37.00 | 6.00 | 25.21 |
|  |  |  |  | 2 / y7 | 582.35 | 788.43 | 31.00 | 31.00 | 0.00 | 25.21 |
|  |  |  |  | 2 / y8 | 582.35 | 887.49 | 31.00 | 31.00 | 0.00 | 25.21 |
|  |  | Light | [2Me]-ELVAENLSVR | 2 / y5 | 579.33 | 588.35 | 30.00 | 37.00 | 7.00 | 25.21 |
|  |  |  |  | 2 / y7 | 579.33 | 788.43 | 30.00 | 31.00 | 1.00 | 25.21 |
|  |  |  |  | 2 / y8 | 579.33 | 887.49 | 30.00 | 31.00 | 1.00 | 25.21 |
| Translin | TSN | Heavy | [2MC]-VVQSLEQTAR | 2 / y4 | 582.84 | 475.26 | 31.00 | 40.00 | 9.00 | 15.93 |
|  |  |  |  | 2 / y7 | 582.84 | 804.42 | 31.00 | 31.00 | 0.00 | 15.93 |
|  |  |  |  | 2 / y8 | 582.84 | 932.48 | 31.00 | 28.00 | -3.00 | 15.93 |
|  |  | Light | [2Me]-VVQSLEQTAR | 2 / y4 | 579.83 | 475.26 | 31.00 | 40.00 | 9.13 | 15.93 |
|  |  |  |  | 2 / y7 | 579.83 | 804.42 | 31.00 | 31.00 | 0.00 | 15.93 |
|  |  |  |  | 2 / y8 | 579.83 | 932.48 | 31.00 | 28.00 | -3.00 | 15.93 |
| Tubulin beta chain | TUBB | Heavy | [2MC]-NSSYFVEWIPNNVK[2MC] | 2 / y5 | 882.98 | 605.38 | 44.00 | 50.00 | 6.00 | 40.33 |
|  |  |  |  | 2 / y6 | 882.98 | 718.47 | 44.00 | 44.00 | 0.00 | 40.33 |
|  |  |  |  | 2 / y7 | 882.98 | 904.55 | 44.00 | 41.00 | -3.00 | 40.33 |
|  |  | Light | [2Me]-NSSYFVEWIPNNVK[2Me] | 2 / y5 | 876.95 | 599.35 | 44.00 | 50.00 | 6.27 | 40.33 |
|  |  |  |  | 2 / y6 | 876.95 | 712.44 | 44.00 | 44.00 | 0.27 | 40.33 |
|  |  |  |  | 2 / y7 | 876.95 | 898.51 | 44.00 | 41.00 | -3.00 | 40.33 |
| Thioredoxin | TXN | Heavy | [2MC]-TAFQEALDAAGDK[2MC] | 2 / y5 | 702.89 | 495.3 | 36.00 | 42.00 | 6.00 | 25.87 |
|  |  |  |  | 2 / y6 | 702.89 | 610.33 | 36.00 | 36.00 | 0.00 | 25.87 |
|  |  |  |  | 2 / y8 | 702.89 | 794.45 | 36.00 | 36.00 | 0.00 | 25.87 |
|  |  | Light | [2Me]-TAFQEALDAAGDK[2Me] | 2 / y5 | 696.85 | 489.27 | 36.00 | 42.00 | 6.27 | 25.87 |
|  |  |  |  | 2 / y6 | 696.85 | 604.29 | 36.00 | 36.00 | 0.27 | 25.87 |
|  |  |  |  | 2 / y8 | 696.85 | 788.41 | 36.00 | 36.00 | 0.00 | 25.87 |
| Ubiquitin-like protein 4A | UBL4A | Heavy | [2MC]-LSDYSIGPNSK[2MC] | 2 / y4 | 624.86 | 479.3 | 32.00 | 41.00 | 9.00 | 21.82 |
|  |  |  |  | 2 / y5 | 624.86 | 536.33 | 32.00 | 41.00 | 9.00 | 21.82 |
|  |  |  |  | 2 / y8 | 624.86 | 899.5 | 32.00 | 35.00 | 3.00 | 21.82 |
|  |  | Light | [2Me]-LSDYSIGPNSK[2Me] | 2 / y4 | 618.83 | 473.27 | 32.00 | 41.00 | 9.27 | 21.82 |
|  |  |  |  | 2 / y5 | 618.83 | 530.29 | 32.00 | 41.00 | 9.27 | 21.82 |
|  |  |  |  | 2 / y8 | 618.83 | 893.47 | 32.00 | 35.00 | 3.00 | 21.82 |
| Isoform 1 of Vesicle-associated membrane protein-associated protein B/C | VAPB | Heavy | [2MC]-TVQSNSPISALAPTGK[2MC] | 2 / y10 | 819.99 | 988.62 | 41.00 | 38.00 | -3.00 | 35.60 |
|  |  |  |  | 2 / y4 | 819.99 | 436.3 | 41.00 | 38.00 | -3.00 | 35.60 |
|  |  |  |  | 2 / y5 | 819.99 | 507.33 | 41.00 | 50.00 | 9.00 | 35.60 |
|  |  | Light | [2Me]-TVQSNSPISALAPTGK[2Me] | 2 / y10 | 813.96 | 982.59 | 41.00 | 38.00 | -3.00 | 35.60 |
|  |  |  |  | 2 / y4 | 813.96 | 430.27 | 41.00 | 38.00 | -2.74 | 35.60 |
|  |  |  |  | 2 / y5 | 813.96 | 501.3 | 41.00 | 50.00 | 9.27 | 35.60 |
| Isoform 1 of Vacuolar protein sorting-associated protein 29 | VPS29 | Heavy | [2MC]-GDFDENLNYPEQK[2MC] | 2 / b4 | 818.91 | 469.21 | 41.00 | 44.00 | 3.00 | 25.44 |
|  |  |  |  | 2 / y4 | 818.91 | 535.33 | 41.00 | 41.00 | 0.00 | 25.44 |
|  |  |  |  | 2 / y6 | 818.91 | 812.44 | 41.00 | 41.00 | 0.00 | 25.44 |
|  |  | Light | [2Me]-GDFDENLNYPEQK[2Me] | 2 / b4 | 812.88 | 463.18 | 41.00 | 44.00 | 3.26 | 25.44 |
|  |  |  |  | 2 / y4 | 812.88 | 529.3 | 41.00 | 41.00 | 0.26 | 25.44 |
|  |  |  |  | 2 / y6 | 812.88 | 806.4 | 41.00 | 41.00 | 0.26 | 25.44 |
| Vitronectin | VTN | Heavy | [2MC]-FEDGVLDPDYPR | 2 / b7 | 728.86 | 810.41 | 37.00 | 31.00 | -6.00 | 29.88 |
|  |  |  |  | 2 / y3 | 728.86 | 435.24 | 37.00 | 46.00 | 9.00 | 29.88 |
|  |  |  |  | 2 / y5 | 728.86 | 647.31 | 37.00 | 40.00 | 3.00 | 29.88 |
|  |  | Light | [2Me]-FEDGVLDPDYPR | 2 / b7 | 725.85 | 804.38 | 37.00 | 31.00 | -5.87 | 29.88 |
|  |  |  |  | 2 / y3 | 725.85 | 435.24 | 37.00 | 46.00 | 9.13 | 29.88 |
|  |  |  |  | 2 / y5 | 725.85 | 647.31 | 37.00 | 40.00 | 3.13 | 29.88 |
| Wiskott-Aldrich syndrome protein family member 2 | WASF2 | Heavy | [2MC]-SDLLSAIR | 2 / y5 | 454.78 | 559.36 | 25.00 | 25.00 | 0.00 | 29.04 |
|  |  |  |  | 2 / y6 | 454.78 | 672.44 | 25.00 | 25.00 | 0.00 | 29.04 |
|  |  |  |  | 2 / y7 | 454.78 | 787.47 | 25.00 | 25.00 | 0.00 | 29.04 |
|  |  | Light | [2Me]-SDLLSAIR | 2 / y5 | 451.77 | 559.36 | 25.00 | 25.00 | 0.00 | 29.04 |
|  |  |  |  | 2 / y6 | 451.77 | 672.44 | 25.00 | 25.00 | 0.00 | 29.04 |
|  |  |  |  | 2 / y7 | 451.77 | 787.47 | 25.00 | 25.00 | 0.00 | 29.04 |
| 14-3-3 protein theta | YWHAQ | Heavy | [2MC]-AVTEQGAELSNEER | 2 / b6 | 783.89 | 620.35 | 39.00 | 39.00 | 0.00 | 16.25 |
|  |  |  |  | 2 / y5 | 783.89 | 634.28 | 39.00 | 39.00 | 0.00 | 16.25 |
|  |  |  |  | 2 / y6 | 783.89 | 747.36 | 39.00 | 36.00 | -3.00 | 16.25 |
|  |  | Light | [2Me]-AVTEQGAELSNEER | 2 / b6 | 780.88 | 614.31 | 39.00 | 39.00 | 0.13 | 16.25 |
|  |  |  |  | 2 / y5 | 780.88 | 634.28 | 39.00 | 39.00 | 0.13 | 16.25 |
|  |  |  |  | 2 / y6 | 780.88 | 747.36 | 39.00 | 36.00 | -2.87 | 16.25 |
| Carbonic anhydrase 2 | CA2 | Heavy | [2MC]-SADFTNFDPR | 2 / b4 | 602.3 | 455.23 | 32.00 | 32.00 | 0.00 | 27.60 |
|  |  |  |  | 2 / b7 | 602.3 | 817.39 | 32.00 | 29.00 | -3.00 | 27.60 |
|  |  |  |  | 2 / y7 | 602.3 | 896.43 | 32.00 | 29.00 | -3.00 | 27.60 |
|  |  | Light | [2Me]-SADFTNFDPR | 2 / b4 | 599.28 | 449.2 | 32.00 | 32.00 | 0.00 | 27.60 |
|  |  |  |  | 2 / b7 | 599.28 | 811.36 | 32.00 | 29.00 | -3.00 | 27.60 |
|  |  |  |  | 2 / y7 | 599.28 | 896.43 | 32.00 | 29.00 | -3.00 | 27.60 |
| Isoform 1 of Lactoylglutathione lyase | GLO1 | Heavy | [2MC]-FEELGVK[2MC] | 2 / y4 | 445.29 | 450.35 | 25.00 | 19.00 | -6.00 | 22.00 |
|  |  |  |  | 2 / y5 | 445.29 | 579.39 | 25.00 | 22.00 | -3.00 | 22.00 |
|  |  |  |  | 2 / y6 | 445.29 | 708.44 | 25.00 | 22.00 | -3.00 | 22.00 |
|  |  | Light | [2Me]-FEELGVK[2Me] | 2 / y4 | 439.26 | 444.32 | 25.00 | 19.00 | -6.00 | 22.00 |
|  |  |  |  | 2 / y5 | 439.26 | 573.36 | 25.00 | 22.00 | -3.00 | 22.00 |
|  |  |  |  | 2 / y6 | 439.26 | 702.4 | 25.00 | 22.00 | -3.00 | 22.00 |
| Ubiquitin-like protein ISG15 | ISG15 | Heavy | [2MC]-LAVHPSGVALQDR | 2 / b4 | 698.91 | 455.32 | 36.00 | 33.00 | -3.00 | 22.90 |
|  |  |  |  | 2 / y7 | 698.91 | 758.42 | 36.00 | 42.00 | 6.00 | 22.90 |
|  |  |  |  | 2 / y9 | 698.91 | 942.5 | 36.00 | 36.00 | 0.00 | 22.90 |
|  |  | Light | [2Me]-LAVHPSGVALQDR | 2 / b4 | 695.89 | 449.29 | 36.00 | 33.00 | -3.00 | 22.90 |
|  |  |  |  | 2 / y7 | 695.89 | 758.42 | 36.00 | 42.00 | 6.00 | 22.90 |
|  |  |  |  | 2 / y9 | 695.89 | 942.5 | 36.00 | 36.00 | 0.00 | 22.90 |
| Importin subunit alpha-4 | KPNA4 | Heavy | [2MC]-GDFGTQK[2MC] | 2 / y4 | 410.75 | 467.3 | 23.00 | 23.00 | 0.00 | 27.20 |
|  |  |  |  | 2 / y5 | 410.75 | 614.37 | 23.00 | 23.00 | 0.00 | 27.20 |
|  |  |  |  | 2 / y6 | 410.75 | 729.4 | 23.00 | 20.00 | -3.00 | 27.20 |
|  |  | Light | [2Me]-GDFGTQK[2Me] | 2 / y4 | 404.71 | 461.27 | 23.00 | 23.00 | 0.00 | 27.20 |
|  |  |  |  | 2 / y5 | 404.71 | 608.34 | 23.00 | 23.00 | 0.00 | 27.20 |
|  |  |  |  | 2 / y6 | 404.71 | 723.37 | 23.00 | 20.00 | -3.00 | 27.20 |
| Isoform 1 of La-related protein 1 | LARP1 | Heavy | [2MC]-SVQPQSHK[2MC]PQPTR | 3 / y3 | 519.98 | 373.22 | 30.00 | 33.00 | 3.00 | 16.70 |
|  |  |  |  | 3 / y5 | 519.98 | 598.33 | 30.00 | 33.00 | 3.00 | 16.70 |
|  |  |  |  | 3 / y6 | 519.98 | 760.49 | 30.00 | 30.00 | 0.00 | 16.70 |
|  |  | Light | [2Me]-SVQPQSHK[2Me]PQPTR | 3 / y3 | 515.95 | 373.22 | 30.00 | 33.00 | 3.00 | 16.70 |
|  |  |  |  | 3 / y5 | 515.95 | 598.33 | 30.00 | 33.00 | 3.00 | 16.70 |
|  |  |  |  | 3 / y6 | 515.95 | 754.46 | 30.00 | 30.00 | 0.00 | 16.70 |
| Interstitial collagenase | MMP1 | Heavy | [2MC]-DIYSSFGFPR | 2 / y6 | 611.82 | 710.36 | 32.00 | 29.00 | -3.00 | 39.90 |
|  |  |  |  | 2 / y7 | 611.82 | 797.39 | 32.00 | 29.00 | -3.00 | 39.90 |
|  |  |  |  | 2 / y8 | 611.82 | 960.46 | 32.00 | 29.00 | -3.00 | 39.90 |
|  |  | Light | [2Me]-DIYSSFGFPR | 2 / y6 | 608.8 | 710.36 | 32.00 | 29.00 | -3.00 | 39.90 |
|  |  |  |  | 2 / y7 | 608.8 | 797.39 | 32.00 | 29.00 | -3.00 | 39.90 |
|  |  |  |  | 2 / y8 | 608.8 | 960.46 | 32.00 | 29.00 | -3.00 | 39.90 |
| Phosphoserine phosphatase | PSPH | Heavy | [2MC]-SIVEHVASK[2MC] | 2 / y5 | 519.33 | 575.37 | 28.00 | 31.00 | 3.00 | 12.40 |
|  |  |  |  | 2 / y7 | 519.33 | 803.48 | 28.00 | 25.00 | -3.00 | 12.40 |
|  |  |  |  | 2 / y8 | 519.33 | 916.57 | 28.00 | 25.00 | -3.00 | 12.40 |
|  |  | Light | [2Me]-SIVEHVASK[2Me] | 2 / y5 | 513.3 | 569.34 | 28.00 | 31.00 | 3.00 | 12.40 |
|  |  |  |  | 2 / y7 | 513.3 | 797.45 | 28.00 | 25.00 | -3.00 | 12.40 |
|  |  |  |  | 2 / y8 | 513.3 | 910.54 | 28.00 | 25.00 | -3.00 | 12.40 |
| Sodium-dependent phosphate transporter 2 | SLC20A2 | Heavy | [2MC]-AADSSAPEDSEK[2MC] | 2 / y3 | 637.82 | 397.25 | 33.00 | 42.00 | 9.00 | 7.50 |
|  |  |  |  | 2 / y6 | 637.82 | 738.37 | 33.00 | 33.00 | 0.00 | 7.50 |
|  |  |  |  | 2 / y9 | 637.82 | 983.47 | 33.00 | 33.00 | 0.00 | 7.50 |
|  |  | Light | [2Me]-AADSSAPEDSEK[2Me] | 2 / y3 | 631.79 | 391.22 | 33.00 | 42.00 | 9.00 | 7.50 |
|  |  |  |  | 2 / y6 | 631.79 | 732.34 | 33.00 | 33.00 | 0.00 | 7.50 |
|  |  |  |  | 2 / y9 | 631.79 | 977.44 | 33.00 | 33.00 | 0.00 | 7.50 |
| Isoform 1 of Stathmin | STMN1 | Heavy | [2MC]-ESVPEFPLSPPK[2MC] | 2 / y3 | 697.91 | 375.28 | 36.00 | 45.00 | 9.00 | 35.70 |
|  |  |  |  | 2 / y6 | 697.91 | 672.45 | 36.00 | 36.00 | 0.00 | 35.70 |
|  |  |  |  | 2 / y7 | 697.91 | 819.52 | 36.00 | 33.00 | -3.00 | 35.70 |
|  |  | Light | [2Me]-ESVPEFPLSPPK[2Me] | 2 / y3 | 691.88 | 369.25 | 36.00 | 45.00 | 9.00 | 35.70 |
|  |  |  |  | 2 / y6 | 691.88 | 666.42 | 36.00 | 36.00 | 0.00 | 35.70 |
|  |  |  |  | 2 / y7 | 691.88 | 813.49 | 36.00 | 33.00 | -3.00 | 35.70 |
| human tenascin-C | TNC | Heavy | [2MC]-YAPISGGDHAEVDVPK[2MC] | 3 / y3 | 574.98 | 377.3 | 43.00 | 33.00 | -10.00 | 24.00 |
|  |  |  |  | 3 / y6 | 574.98 | 720.44 | 43.00 | 30.00 | -13.00 | 24.00 |
|  |  |  |  | 3 / y7 | 574.98 | 791.47 | 43.00 | 30.00 | -13.00 | 24.00 |
|  |  | Light | [2Me]-YAPISGGDHAEVDVPK[2Me] | 3 / y3 | 570.96 | 371.27 | 43.00 | 33.00 | -10.00 | 24.00 |
|  |  |  |  | 3 / y6 | 570.96 | 714.4 | 43.00 | 30.00 | -13.00 | 24.00 |
|  |  |  |  | 3 / y7 | 570.96 | 785.44 | 43.00 | 30.00 | -13.00 | 24.00 |
| Transgelin-2 | TAGLN2 | Heavy | [2MC]-GPAYGLSR | 2 / y4 | 427.75 | 432.26 | 24.00 | 24.00 | 0.00 | 25.30 |
|  |  |  |  | 2 / y6 | 427.75 | 666.36 | 24.00 | 24.00 | 0.00 | 25.30 |
|  |  |  |  | 2 / y7 | 427.75 | 763.41 | 24.00 | 24.00 | 0.00 | 25.30 |
|  |  | Light | [2Me]-GPAYGLSR | 2 / y4 | 424.73 | 432.26 | 24.00 | 24.00 | 0.00 | 25.30 |
|  |  |  |  | 2 / y6 | 424.73 | 666.36 | 24.00 | 24.00 | 0.00 | 25.30 |
|  |  |  |  | 2 / y7 | 424.73 | 763.41 | 24.00 | 24.00 | 0.00 | 25.30 |
