# Supplementary material for: Systematic verification of bladder cancer-associated tissue protein biomarker candidates in clinical urine specimens
Source: Oncotarget. 2018 Jul 20;9(56):30731–47. doi: 10.18632/oncotarget.24578 (PMC6089400; doi:10.18632/oncotarget.24578)
Supplement: Supplementary file 5 [file oncotarget-09-30731-s005.docx]

**Supplementary Table 4: The CV values for quantifying 122 bladder cancer associated tissue proteins in clinical urine specimens**

| Targets |  | Total 4 group (n=119) | | |  | Hernia (n=31) | | |  | Bladder cancer (n=30) | | |  | Hematuria (n=30) | | |  | Urinary tract infection (n=28) | | |
| --- | --- | --- | --- | --- | --- | --- | --- | --- | --- | --- | --- | --- | --- | --- | --- | --- | --- | --- | --- | --- |
|  |  | Average | Median | SD |  | Average | Median | SD |  | Average | Median | SD |  | Average | Median | SD |  | Average | Median | SD |
| ACTB |  | 22.7% | 13.3% | 30.9% |  | 15.0% | 14.3% | 9.2% |  | 27.9% | 17.1% | 29.3% |  | 27.2% | 12.2% | 46.5% |  | 20.7% | 12.0% | 26.7% |
| ANXA3 |  | 80.2% | 86.8% | 71.3% |  | N/A | N/A | N/A |  | 37.2% | 20.8% | 45.7% |  | 172.1% | 172.1% | 1.5% |  | 52.6% | 52.6% | 48.3% |
| BAIAP2 |  | 54.1% | 54.1% | 52.2% |  | 17.2% | 17.2% | N/A |  | 90.9% | 90.9% | N/A |  | N/A | N/A | N/A |  | N/A | N/A | N/A |
| CA2 |  | 13.1% | 13.1% | N/A |  | N/A | N/A | N/A |  | 13.1% | 13.1% | N/A |  | N/A | N/A | N/A |  | N/A | N/A | N/A |
| CLTA |  | 24.7% | 24.7% | 6.2% |  | N/A | N/A | N/A |  | 24.7% | 24.7% | 6.2% |  | N/A | N/A | N/A |  | N/A | N/A | N/A |
| GAA |  | 48.5% | 17.7% | 55.1% |  | 30.0% | 15.7% | 39.9% |  | 25.7% | 14.6% | 28.9% |  | 83.9% | 86.6% | 69.3% |  | 54.5% | 21.9% | 53.7% |
| GLO1 |  | 20.1% | 20.1% | 2.2% |  | N/A | N/A | N/A |  | 20.1% | 20.1% | 2.2% |  | N/A | N/A | N/A |  | N/A | N/A | N/A |
| HSP90AB1 |  | 28.8% | 16.4% | 34.0% |  | 15.9% | 13.3% | 9.9% |  | 30.0% | 21.4% | 24.6% |  | 37.6% | 17.0% | 47.5% |  | 27.9% | 14.3% | 37.2% |
| HSPE1 |  | 26.8% | 27.8% | 10.9% |  | N/A | N/A | N/A |  | 21.7% | 21.7% | 8.7% |  | N/A | N/A | N/A |  | 37.2% | 37.2% | N/A |
| LAMP2 |  | 36.1% | 22.7% | 38.7% |  | 27.3% | 24.0% | 25.7% |  | 53.6% | 36.9% | 50.1% |  | 39.1% | 19.9% | 43.6% |  | 21.0% | 19.1% | 13.2% |
| LOC643576 |  | 25.0% | 25.0% | N/A |  | N/A | N/A | N/A |  | N/A | N/A | N/A |  | 25.0% | 25.0% | N/A |  | N/A | N/A | N/A |
| PSPH |  | 34.0% | 26.6% | 27.5% |  | 23.9% | 24.7% | 15.7% |  | 41.9% | 35.3% | 31.8% |  | N/A | N/A | N/A |  | 13.9% | 13.9% | N/A |
| RAB11B |  | 65.6% | 86.9% | 48.7% |  | 70.5% | 86.9% | 32.1% |  | 64.4% | 89.4% | 39.9% |  | 65.2% | 30.9% | 68.8% |  | 59.8% | 19.7% | 73.7% |
| SFN |  | 7.0% | 7.0% | N/A |  | N/A | N/A | N/A |  | 7.0% | 7.0% | N/A |  | N/A | N/A | N/A |  | N/A | N/A | N/A |
| TAGLN2 |  | 9.3% | 9.3% | 1.1% |  | N/A | N/A | N/A |  | 9.3% | 9.3% | 1.1% |  | N/A | N/A | N/A |  | N/A | N/A | N/A |
| TIMM13 |  | 121.2% | 118.9% | 44.4% |  | 173.2% | 173.2% | N/A |  | 140.4% | 140.4% | N/A |  | 97.4% | 97.4% | N/A |  | 73.6% | 73.6% | N/A |
| TPI1 |  | 22.9% | 17.1% | 18.8% |  | 22.4% | 16.1% | 17.7% |  | 18.7% | 16.4% | 13.5% |  | 27.2% | 20.0% | 22.3% |  | 23.7% | 17.8% | 21.7% |
| VPS29 |  | 130.5% | 130.5% | 60.4% |  | N/A | N/A | N/A |  | 130.5% | 130.5% | 60.4% |  | N/A | N/A | N/A |  | N/A | N/A | N/A |
| VTN |  | 56.6% | 22.1% | 58.9% |  | 50.7% | 21.5% | 61.0% |  | 59.6% | 44.6% | 54.7% |  | 77.2% | 86.7% | 67.3% |  | 36.4% | 12.5% | 44.5% |
| WASF2 |  | 134.6% | 134.6% | 54.6% |  | 173.2% | 173.2% | N/A |  | N/A | N/A | N/A |  | N/A | N/A | N/A |  | 95.9% | 95.9% | N/A |
| YWHAQ |  | 77.0% | 36.9% | 83.7% |  | N/A | N/A | N/A |  | 77.0% | 36.9% | 83.7% |  | N/A | N/A | N/A |  | N/A | N/A | N/A |
| DPP7 |  | 60.8% | 32.4% | 58.3% |  | 56.1% | 21.4% | 59.6% |  | 60.8% | 38.8% | 59.3% |  | 57.7% | 16.9% | 63.5% |  | 68.2% | 86.7% | 54.8% |
| ENO1 |  | 64.6% | 70.2% | 58.4% |  | N/A | N/A | N/A |  | 114.7% | 133.3% | 74.0% |  | 56.4% | 18.2% | 62.5% |  | 48.8% | 53.8% | 38.8% |
| IGFBP7 |  | 40.3% | 16.0% | 48.3% |  | 33.4% | 13.3% | 44.9% |  | 52.1% | 23.4% | 62.3% |  | 37.8% | 15.7% | 41.5% |  | 37.6% | 15.8% | 41.3% |
| PRPF3 |  | 114.9% | 120.9% | 44.3% |  | 136.4% | 136.4% | N/A |  | 132.8% | 134.8% | 36.4% |  | 81.4% | 93.2% | 49.9% |  | 128.6% | 120.6% | 41.2% |
| RNASET2 |  | 26.3% | 16.2% | 31.2% |  | 28.4% | 17.2% | 35.8% |  | 24.0% | 15.5% | 27.7% |  | 26.5% | 17.2% | 27.9% |  | 25.3% | 13.2% | 32.9% |
| SERPINA3 |  | 14.0% | 8.3% | 19.2% |  | 15.3% | 8.5% | 18.3% |  | 11.2% | 8.4% | 7.0% |  | 12.9% | 7.8% | 14.7% |  | 16.8% | 6.6% | 31.1% |
| SERPINB5_maspin |  | 23.1% | 23.1% | N/A |  | N/A | N/A | N/A |  | 23.1% | 23.1% | N/A |  | N/A | N/A | N/A |  | N/A | N/A | N/A |
| SLC3A2 |  | 119.8% | 119.8% | 46.9% |  | N/A | N/A | N/A |  | 119.8% | 119.8% | 46.9% |  | N/A | N/A | N/A |  | N/A | N/A | N/A |
