# Supplementary material for: Systematic verification of bladder cancer-associated tissue protein biomarker candidates in clinical urine specimens
Source: Oncotarget. 2018 Jul 20;9(56):30731–47. doi: 10.18632/oncotarget.24578 (PMC6089400; doi:10.18632/oncotarget.24578)
Supplement: Supplementary file 6 [file oncotarget-09-30731-s006.docx]

**Supplementary Table 7: The IHC scores of expressions of HSPE1 in clinical tissue slides of BC patients**

| Slide No. | Normal | | | Tumor | | |
| --- | --- | --- | --- | --- | --- | --- |
|  | Intensity | % | score | Intensity | % | score |
| 1 | + | 10 | 10 | +++ | 30 | 90 |
| 2 | + | 10 | 10 | +++ | 70 | 210 |
| 3 | + | 10 | 10 | +++ | 30 | 90 |
| 4 | + | 20 | 20 | +++ | 85 | 255 |
| 5 | + | 20 | 20 | ++ | 60 | 120 |
| 6 | ++ | 10 | 20 | +++ | 50 | 150 |
| 7 | + | 20 | 20 | + | 40 | 40 |
| 8 | + | 50 | 50 | +++ | 60 | 180 |
| 9 | + | 50 | 50 | ++ | 60 | 120 |
| 10 | + | 60 | 60 | +++ | 95 | 285 |
| 11 | + | 60 | 60 | +++ | 70 | 210 |
| 12 | + | 95 | 95 | ++ | 95 | 190 |
| 13 | ++ | 50 | 100 | +++ | 80 | 240 |
| 14 | ++ | 50 | 100 | +++ | 95 | 285 |
| 15 | ++ | 50 | 100 | +++ | 90 | 270 |
| 16 | ++ | 70 | 140 | ++ | 90 | 180 |
| 17 | ++ | 90 | 180 | +++ | 60 | 180 |
| 18 | ++ | 95 | 190 | +++ | 95 | 285 |
| 19 | ++ | 100 | 200 | ++ | 90 | 180 |
| 20 |  |  |  | +++ | 95 | 285 |
| 21 |  |  |  | +++ | 95 | 285 |
| 22 |  |  |  | +++ | 80 | 240 |
| 23 |  |  |  | +++ | 90 | 270 |
| 24 |  |  |  | +++ | 95 | 285 |
| 25 |  |  |  | +++ | 80 | 240 |
| 26 |  |  |  | +++ | 50 | 150 |
| 27 |  |  |  | +++ | 40 | 120 |
| 28 |  |  |  | +++ | 20 | 60 |
| 29 |  |  |  | +++ | 30 | 90 |
| 30 |  |  |  | +++ | 60 | 180 |
| 31 |  |  |  | ++ | 70 | 140 |
| 32 |  |  |  | +++ | 95 | 285 |
| 33 |  |  |  | +++ | 90 | 270 |
| 34 |  |  |  | +++ | 95 | 285 |
| 35 |  |  |  | +++ | 95 | 285 |
| 36 |  |  |  | +++ | 50 | 150 |
| 37 |  |  |  | ++ | 70 | 140 |
| 38 |  |  |  | ++ | 10 | 20 |
| 39 |  |  |  | +++ | 80 | 240 |
| 40 |  |  |  | +++ | 40 | 120 |
| 41 |  |  |  | +++ | 95 | 285 |
| 42 |  |  |  | +++ | 90 | 270 |
| 43 |  |  |  | +++ | 80 | 240 |
| 44 |  |  |  | +++ | 95 | 285 |
| 45 |  |  |  | +++ | 95 | 285 |
| 46 |  |  |  | +++ | 95 | 285 |
| 47 |  |  |  | +++ | 95 | 285 |
| 48 |  |  |  | +++ | 40 | 120 |
| 49 |  |  |  | ++ | 75 | 150 |
| 50 |  |  |  | +++ | 30 | 90 |
| 51 |  |  |  | +++ | 60 | 180 |
| 52 |  |  |  | ++ | 80 | 160 |
| 53 |  |  |  | +++ | 90 | 270 |
| 54 |  |  |  | +++ | 80 | 240 |
| 55 |  |  |  | +++ | 95 | 285 |
| 56 |  |  |  | +++ | 95 | 285 |
| 57 |  |  |  | +++ | 80 | 240 |
| 58 |  |  |  | +++ | 95 | 285 |
| 59 |  |  |  | +++ | 95 | 285 |
| 60 |  |  |  | +++ | 95 | 285 |
| 61 |  |  |  | +++ | 95 | 285 |
| 62 |  |  |  | +++ | 95 | 285 |
| 63 |  |  |  | ++ | 80 | 160 |
| 64 |  |  |  | +++ | 70 | 210 |
| 65 |  |  |  | +++ | 95 | 285 |
| 66 |  |  |  | +++ | 95 | 285 |
| 67 |  |  |  | +++ | 95 | 285 |
| 68 |  |  |  | +++ | 90 | 270 |
| 69 |  |  |  | +++ | 60 | 180 |
| 70 |  |  |  | +++ | 90 | 270 |
| 71 |  |  |  | +++ | 90 | 270 |
| 72 |  |  |  | +++ | 90 | 270 |
| 73 |  |  |  | +++ | 95 | 285 |
| 74 |  |  |  | ++ | 90 | 180 |
| 75 |  |  |  | +++ | 95 | 285 |
| 76 |  |  |  | +++ | 90 | 270 |
| 77 |  |  |  | +++ | 95 | 285 |
| 78 |  |  |  | ++ | 80 | 160 |
| 79 |  |  |  | +++ | 80 | 240 |
| 80 |  |  |  | +++ | 80 | 240 |
| 81 |  |  |  | ++ | 70 | 140 |
| 82 |  |  |  | +++ | 95 | 285 |
| 83 |  |  |  | ++ | 60 | 120 |
| 84 |  |  |  | ++ | 95 | 190 |
| 85 |  |  |  | +++ | 90 | 270 |
| 86 |  |  |  | +++ | 90 | 270 |
| 87 |  |  |  | +++ | 70 | 210 |
| 88 |  |  |  | +++ | 90 | 270 |
| 89 |  |  |  | +++ | 90 | 270 |
| 90 |  |  |  | +++ | 95 | 285 |
| 91 |  |  |  | +++ | 95 | 285 |
| 92 |  |  |  | +++ | 80 | 240 |
